# Survival trade-offs in plant roots during colonization by closely related beneficial and pathogenic fungi

**DOI:** 10.1038/ncomms11362

**Published:** 2016-05-06

**Authors:** Stéphane Hacquard, Barbara Kracher, Kei Hiruma, Philipp C. Münch, Ruben Garrido-Oter, Michael R. Thon, Aaron Weimann, Ulrike Damm, Jean-Félix Dallery, Matthieu Hainaut, Bernard Henrissat, Olivier Lespinet, Soledad Sacristán, Emiel Ver Loren van Themaat, Eric Kemen, Alice C. McHardy, Paul Schulze-Lefert, Richard J. O'Connell

**Affiliations:** 1 Department of Plant Microbe Interactions, Max Planck Institute for Plant Breeding Research, 50829 Cologne, Germany; 2 German Center for Infection Research (DZIF), Partner Site Hannover-Braunschweig, 38124 Braunschweig, Germany; 3 Computational Biology of Infection Research, Helmholtz Center for Infection Research, 38124 Braunschweig, Germany; 4 Max-von-Pettenkofer Institute, LMU Munich, German Center for Infection Research (DZIF), Partner Site LMU Munich, 80336 Munich, Germany; 5 Department of Algorithmic Bioinformatics, Heinrich Heine University Duesseldorf, 40225 Duesseldorf, Germany; 6 Cluster of Excellence on Plant Sciences (CEPLAS), Max Planck Institute for Plant Breeding Research, 50829 Cologne, Germany; 7 Instituto Hispano-Luso de Investigaciones Agrarias (CIALE), Departamento de Microbiología y Genética, Universidad de Salamanca, 37185 Villamayor, Spain; 8 CBS-KNAW Fungal Biodiversity Centre, 3584 CT Utrecht, The Netherlands; 9 UMR BIOGER, INRA, AgroParisTech, Université Paris-Saclay, 78850 Thiverval-Grignon, France; 10 CNRS UMR 7257, Aix-Marseille University, 13288 Marseille, France; 11 INRA, USC 1408 AFMB, 13288 Marseille, France; 12 Department of Biological Sciences, King Abdulaziz University, 21589 Jeddah, Saudi Arabia; 13 Institute for Integrative Biology of the Cell (I2BC), CEA, CNRS, Université Paris-Sud, 91405 Orsay, France; 14 Laboratoire de Recherche en Informatique, CNRS, Université Paris-Sud, 91405 Orsay, France; 15 Centro de Biotecnología y Genómica de Plantas (UPM-INIA) and E.T.S.I. Agrónomos, Universidad Politécnica de Madrid Campus de Montegancedo, 28223 Madrid, Spain

## Abstract

The sessile nature of plants forced them to evolve mechanisms to prioritize their responses to simultaneous stresses, including colonization by microbes or nutrient starvation. Here, we compare the genomes of a beneficial root endophyte, *Colletotrichum tofieldiae* and its pathogenic relative *C. incanum*, and examine the transcriptomes of both fungi and their plant host *Arabidopsis* during phosphate starvation. Although the two species diverged only 8.8 million years ago and have similar gene arsenals, we identify genomic signatures indicative of an evolutionary transition from pathogenic to beneficial lifestyles, including a narrowed repertoire of secreted effector proteins, expanded families of chitin-binding and secondary metabolism-related proteins, and limited activation of pathogenicity-related genes *in planta*. We show that beneficial responses are prioritized in *C. tofieldiae*-colonized roots under phosphate-deficient conditions, whereas defense responses are activated under phosphate-sufficient conditions. These immune responses are retained in phosphate-starved roots colonized by pathogenic *C. incanum*, illustrating the ability of plants to maximize survival in response to conflicting stresses.

## Abstract (web summary)

*Colletotrichum tofieldiae* is a beneficial root endophyte, whereas the closely related *C. incanum* is pathogenic. Here the authors compare the genomes and transcriptomes during host plant interaction and demonstrate that the host plant can respond differently to the beneficial endophyte according to phosphate status.

## Graphical abstract

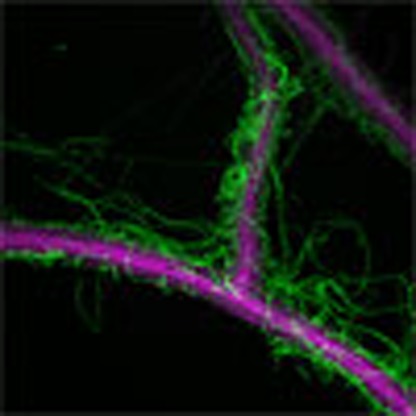

Fungal endophytes are a ubiquitous and phylogenetically diverse group of organisms that establish stable associations with living plants, but in most cases their ecophysiological significance is poorly understood^1^. Species of the fungal genus *Colletotrichum* are best known as destructive pathogens on >3,000 species of dicot and monocot plants worldwide, causing anthracnose diseases and blights on leaves, stems, flowers and fruits^2^. However *Colletotrichum* species can also grow benignly as endophytes on symptomless plants^3^, and although only few pathogenic members of the genus attack plant roots^4^, *Colletotrichum* endophytes are frequently isolated from the roots of healthy plants^5^,^6^. Moreover, although the genome sequences and *in planta* transcriptomes were recently described for four species pathogenic on above-ground plant parts^2^,^7^, such information is not available for any root-associated *Colletotrichum* pathogens or endophytes.

We found recently that *C. tofieldiae* (*Ct*) is an endophyte in natural populations of *Arabidopsis thaliana* growing in central Spain^8^. The fungus initially penetrates the rhizoderm by means of undifferentiated hyphae, which then ramify through the root cortex both inter- and intracellularly, occasionally spreading systemically into shoots via the root central cylinder without causing visible symptoms. Under phosphate-deficient conditions (50 μM KH_2_PO_4_), colonization by *Ct* promoted plant growth and fertility and mediated the translocation of phosphate into shoots, as shown by ^33^P radiotracer experiments^8^. However, neither the plant growth promotion nor phosphate translocation activities were detectable under phosphate-sufficient conditions (625 μM KH_2_PO_4_), indicating that plant fitness benefits conferred by *Ct* are strictly regulated by phosphate availability. In striking contrast, colonization of *A. thaliana* roots by the closely related pathogenic species *C. incanum* (*Ci*), which attacks members of the Brassicaceae, Fabaceae and Solanaceae, severely inhibited *Arabidopsis* growth and mediated only low levels of ^33^P translocation into shoots^8^. These findings raise the possibility that in low-phosphate soils, root colonization by the *Ct* endophyte compensates for the absence of key genetic components required for mycorrhizal symbiosis in the Brassicaceae lineage, which is otherwise conserved in ∼80–90% of terrestrial plants^9^.

In the present study, we report the genomes of five isolates of beneficial *Ct* and one isolate of pathogenic *Ci*, and analyse the transcriptomes of each species during their colonization of *Arabidopsis* roots under phosphate-deficient and phosphate-sufficient conditions. Comparison of the two species allows us to identify fungal adaptations to the endophytic lifestyle at the level of both gene repertoire and gene regulation, and provides insights into the evolutionary transition from parasitism to endophytism within a single fungal genus. On the host side, transcriptional responses of *Arabidopsis* roots to colonization by beneficial *Ct* are modulated by the phosphate status, providing evidence that trade-offs between defense and nutrition control the outcome of the interaction between *Arabidopsis* and *Ct*. Our findings also shed light on the ability of plants to maximize survival by prioritizing their responses to simultaneous biotic and abiotic stresses.

## Results

### Genome sequencing and evolution of *Ct* and *Ci* lifestyles

**Figure 1:**
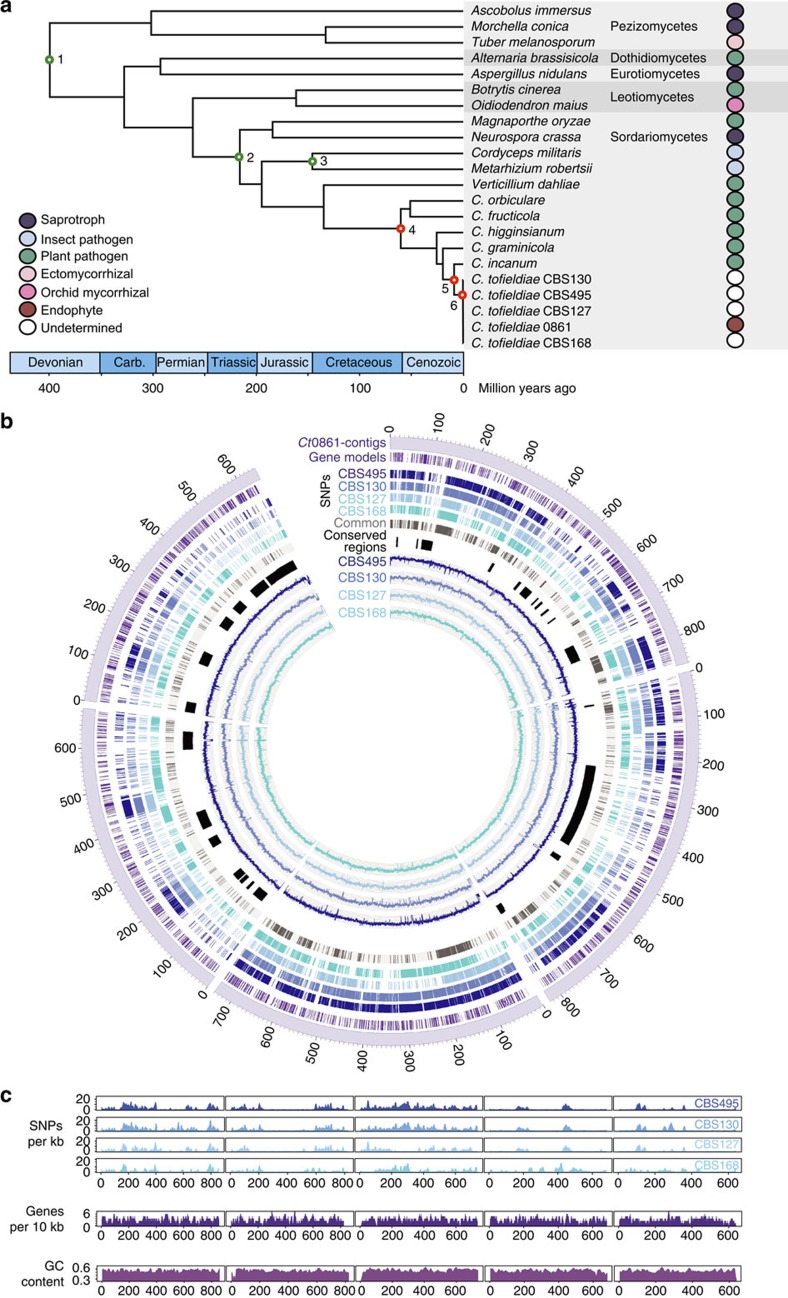
*Colletotrichum* evolutionary divergence dates and SNP distribution in *C. tofieldiae* isolates. (**a**) Phylogeny of *Colletotrichum* species inferred from analysing 20 single-copy gene families using PhyML and r8s. Nodes 1–3 (green) are calibration points and nodes 4, 5 and 6 (red) represent estimated divergence dates (see Supplementary Note 3). (**b**) Circular visualization of the alignment of genome sequencing reads and SNP locations of four *C. tofieldiae* isolates with respect to the *Ct*0861 reference assembly. Tracks represent (from the outside) the five largest *Ct*0861 contigs (scale: kb); locations of predicted genes; locations of SNPs versus *Ct*0861 in CBS495, CBS130, CBS127, CBS168 (see Supplementary Table 1 for full culture IDs) and SNPs common to these four isolates; conserved regions with low SNP density between all the five isolates; mean read coverage (per 100 bases) for isolates CBS495, CBS130, CBS127 and CBS168. Coverage plot scales are 0 to 1,000 (CBS495) or 0 to 500 (CBS130, 127, 168). (**c**) SNP density (per 1 kb) in isolates CBS495, CBS130, CBS127 and CBS168 versus *Ct*0861, compared with gene density (per 10 kb) and GC content (%) on the five largest *Ct*0861 contigs.

We sequenced the genome of the plant growth-promoting fungus *Ct* isolate 0861, a root endophyte isolated from natural populations of *A. thaliana* in Spain^8^,^10^, and those of four other *Ct* isolates isolated from diverse dicot and monocot hosts in Europe (Supplementary Note 1). We also sequenced the broad host-range pathogen *Ci*, isolated from radish (*Raphanus sativus*) leaves in Japan, that strongly impairs plant growth when inoculated onto *Arabidopsis* roots^8^,^11^ (Supplementary Fig. 1, Supplementary Table 1 and Supplementary Note 1). Illumina short reads were used to build high-quality genome assemblies of similar size for all isolates, ranging from 52.8 to 53.6 Mb (Supplementary Table 2 and Supplementary Note 2). Molecular phylogeny, whole-genome alignment and divergence date estimates indicate that *Ct* and *Ci* are closely related taxa within the *Colletotrichum spaethianum* species complex and diverged only ∼8.8 million years ago (Fig. 1a, Supplementary Figs 2 and 3, Supplementary Table 3 and Supplementary Note 3). Our phylogenetic analysis suggests that evolution from pathogenic ancestors towards the beneficial endophytic lifestyle in *Ct* is a recent adaptation in *Colletotrichum* fungi.

### SNP distribution and reproductive mode of *Ct* isolates

Although the five *Ct* isolates originate from widely separated geographical areas and distantly related plant hosts, they diverged only ∼0.29 million years ago and the aligned fractions (>93%) of their genomes share >99% sequence identity (Fig. 1a and Supplementary Tables 1,3 and 4). The overall frequency of single-nucleotide polymorphisms (SNPs) between isolates was similar (2.22–3.04 SNPs per kb) but the SNP distribution within each genome was uneven, with alternating tracts of low (0.22–0.32 SNPs per kb) and high (4.25–5.12 SNPs per kb) SNP density (Fig. 1b, Supplementary Fig. 4 and Supplementary Table 5). This peculiar SNP distribution, also visible in the genomes of other plant-interacting fungi^12^,^13^, is consistent with chromosome recombination events. However, the SNP density profiles are remarkably similar between isolates and large haplotype blocks are conserved between all (21%), four (19%), three (18%) or two (17%) of them, with only 22% being isolate specific (Fig. 1b,c, Supplementary Fig. 4, Supplementary Table 6 and Supplementary Note 4). These conserved SNP signatures in the genomes of geographically distant isolates were likely generated by rare or ancestral sexual/parasexual reproduction and maintained by frequent clonal propagation.

### Evolutionary dynamics of multigene families in *Colletotrichum*

**Figure 2:**
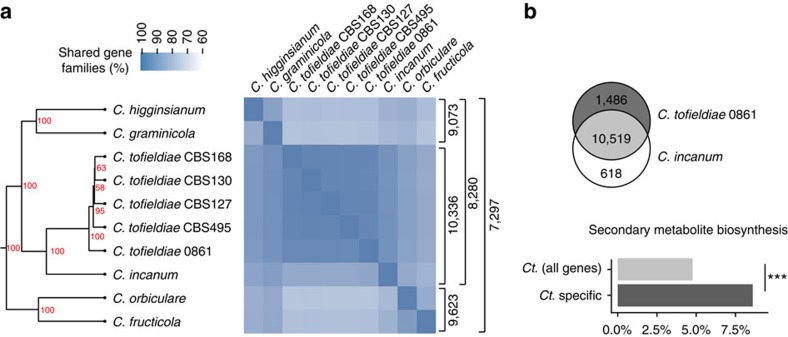
Conservation of orthoMCL gene families within the proteomes of *Colletotrichum* species. (**a**) Heatmap and hierarchical clustering dendrogram depicting the percentage of gene families shared between 10 *Colletotrichum* genomes. Node labels in the tree indicate bootstrap support after 100 iterations. Brackets (right-hand side) indicate the number of gene families shared between the groups of genomes. (**b**) Upper panel: Venn diagram of gene families shared between the beneficial *C. tofieldiae* 0861 and its close pathogenic relative, C. *incanum*. Lower panel: Barplot showing the over-abundance of proteins related to secondary metabolite biosynthesis among gene families unique to *Ct*0861 compared with all *C. tofieldiae* gene families (Fisher's exact test; ***P=3.31E−08).

Similar numbers of protein-coding genes were predicted in *Ct*0861 and *Ci* (∼13,000; Supplementary Table 2), with >11,300 orthologous genes shared between both species. By clustering protein-coding sequences into sets of orthologous genes using OrthoMCL, we identified 7,297 gene families conserved across all six analysed *Colletotrichum* species and 10,519 shared between *Ct*0861 and *Ci* (Fig. 2a,b and Supplementary Note 5). Using a maximum-likelihood approach, we also reconstructed ancestral genomes for each *Colletotrichum* lineage and predicted the number of gene families that were likely gained or lost in each species compared with its corresponding ancestor (Supplementary Fig. 5 and Supplementary Note 6). We found significantly more gene families gained (1,009) than lost (198) on the branch leading to *Ct* compared with other branches of the tree (Fisher's exact test*, P*=3.98 × 10^−136^; Supplementary Fig. 5 and Supplementary Data 1). Functional enrichment analysis among the 1,009 gene families gained (Supplementary Fig. 5) and the 1,486 *Ct*-specific gene families (Fig. 2b) revealed a significant enrichment for genes encoding secondary metabolite biosynthesis-related proteins in *Ct* (Fisher's exact test*, P*=5.89 × 10^−3^ and 3.31 × 10^−8^, respectively). This result contrasts with the very low number of secondary metabolite-related genes detected in the genomes of other root-associated fungal endophytes and mycorrhizal fungi^14^ and suggests that either fungal secondary metabolites have roles in establishing a beneficial endophytic interaction with host plants or in limiting the colonization of microbial competitors inside roots. Evaluation of the selective forces (*d*_N_/*d*_S_ ratio) acting on all the protein families in the *Ct* genome revealed that genes involved in ‘signal transduction mechanisms', ‘RNA processing and modification' and ‘lipid transport and metabolism' showed the strongest evidence of adaptive evolution (false discovery rate (FDR)<0.05, Fisher's test). This contrasts with pathogenic *Colletotrichum* species for which gene families belonging to the categories ‘defense mechanisms', ‘cell wall/membrane/envelope biogenesis' and ‘RNA processing and modification' show the highest *d*_N_/*d*_S_ ratios (Supplementary Figs 6 and 7, Supplementary Data 2 and Supplementary Note 7).

### Genomic signatures of the pathogenic to beneficial transition

*Ct* encodes large repertoires of transporters, secreted proteins, proteases, carbohydrate-active enzymes (CAZymes) and secondary metabolism key enzymes, very similar to *Ci* and four other pathogenic *Colletotrichum* species (Supplementary Figs 8–12 and Supplementary Note 8). By comparing the *Ct* gene repertoires to those of five other plant-associated fungal endophytes from both ascomycete and basidiomycete lineages, we found no obvious common genomic signatures to indicate the convergent evolution of an endophyte ‘toolkit' (Supplementary Figs 8–12). Furthermore, the convergent loss of decay mechanisms characteristic of ectomycorrhizal fungi^15^,^16^ is not a hallmark shared by the non-mycorrhizal root endophytes (Supplementary Fig. 12), suggesting that these fungi have followed different evolutionary trajectories to acquire the ability for intimate growth in living root tissues^14^,^17^.

**Figure 3:**
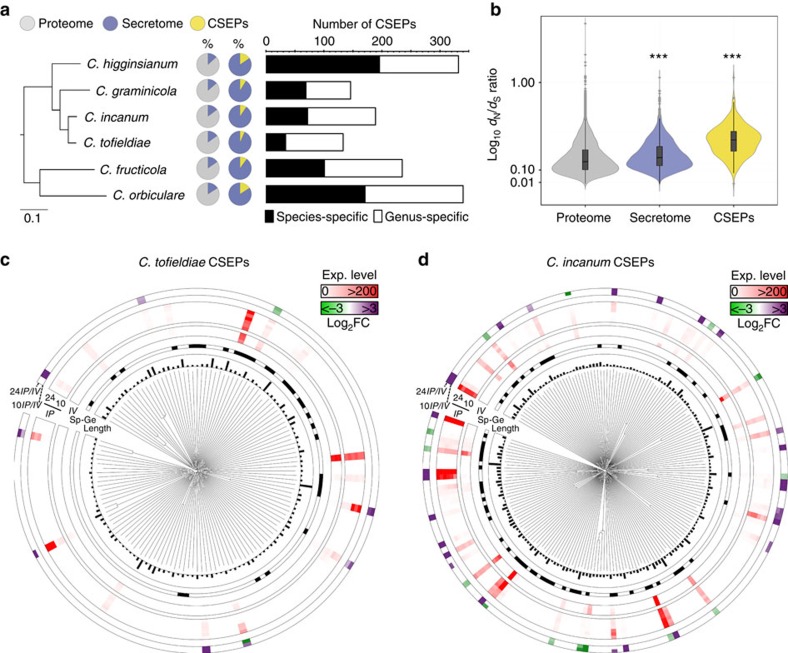
Conservation and expression of genes encoding candidate secreted effector proteins in *C. tofieldiae* and *C. incanum.* (**a**) Proportions of predicted secreted proteins (circles, violet sectors) and candidate secreted effector proteins CSEPs (circles, yellow sectors) in the proteomes and secretomes of *Colletotrichum* species, respectively. The number of genus- and species-specific CSEPs detected for each species is indicated in the barplot. (**b**) Boxplot with a rotated kernel density on each side showing *d*_N_/*d*_S_ ratio (log_10_) measured in the proteome, the secretome and the CSEP repertoires of 10 *Colletotrichum* isolates using the gene families defined by MCL clustering (see Fig. 2). The overall *d*_N_/*d*_S_ ratio is significantly higher for gene families encoding secreted proteins and CSEPs compared with the remaining gene families (One-sided Fisher's test, ***P<0.001). (**c**,**d**) Expression and regulation of *CSEPs* in *C. tofieldiae* 0861 (**c**) and *C. incanum* (**d**). The circular plots show (from the inside): dendrograms of the CSEPs based on protein sequence alignments, CSEP length (0–500 amino acids), species-specific (Sp, black) and genus-specific (Ge, white) CSEPs, normalized gene expression (Exp.) levels *in vitro* (*IV*) and *in planta* (*IP*) at 10 and 24 days post inoculation, *CSEPs* significantly up- (violet) and downregulated (green) at 10 days post inoculation versus *in vitro* (10*IP*/*IV*) and 24 days post inoculation versus *in vitro* (24*IP*/*IV*) (|log_2_FC|⩾1, FDR<0.05).

Despite the overall similar secretome size of all analysed *Colletotrichum* species (13.3–15.9% of the total proteome), the proportion of genes encoding candidate secreted effector proteins (CSEPs), which may promote fungal infection^18^, varied considerably between species (6.6–15.8% of the total secretome; Fig. 3a and Supplementary Table 7). The smaller CSEP repertoire in *Ct*0861 (133 versus 189 in *Ci*) is largely explained by the reduction of species-specific CSEPs (34 versus 72 in *Ci*; Fig. 3a, Supplementary Fig. 13, Supplementary Table 7 and Supplementary Data 3). As expected, calculation of *d*_N_/*d*_S_ ratios among 331 *CSEP* families derived from all the 10 analysed *Colletotrichum* genomes indicates they are under diversifying selection (median 0.35, interquartile range 0.21–0.49) relative to non-CSEP families (median 0.20, interquartile range 0.07–0.33; Fisher's exact test, *P*<2.2 × 10^−16^; Fig. 3b). Genomes from additional *Ci* isolates are now needed to determine whether there is differential host-selective pressure on the CSEP repertoires of endophytic *Ct* and pathogenic *Ci* that reflect their contrasting lifestyles. Similar to other *Colletotrichum* species^2^, *CSEPs* in *Ct* and *Ci* are not organized into large multigene families, possibly due to a low frequency of duplication events in their respective genomes (Fig. 3c,d and Supplementary Table 2).

**Figure 4:**
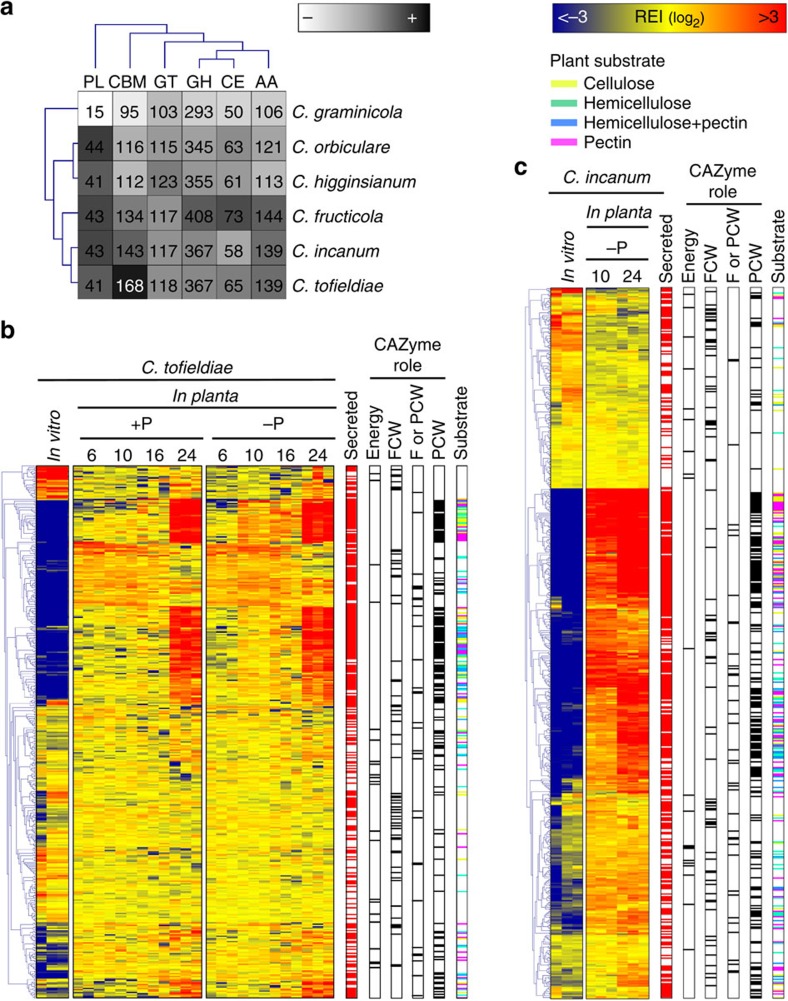
*Colletotrichum* CAZyme repertoires and their transcriptional regulation in *C. tofieldiae* and *C. incanum*. (**a**) Hierarchical clustering of CAZyme classes from the genomes of four *Colletotrichum* species. AA, auxiliary activities; CBM, carbohydrate-binding module CE, carbohydrate esterase; GH, glycoside hydrolase; GT, glycosyltransferase; PL, polysaccharide lyase. The numbers of enzyme modules in each genome are shown. Overrepresented (dark grey to black) and underrepresented (pale grey to white) modules are depicted as log_2_ (fold changes) relative to the class mean. (**b**) Transcript profiling of *C. tofieldiae* CAZyme genes *in vitro* and during colonization of *Arabidopsis* roots at 6, 10, 16 and 24 days post inoculation (d.p.i.) under phosphate sufficient (+P: [625 μM]) and deficient (−P: [50 μM]) conditions. (**c**) Transcript profiling of *C. incanum* CAZyme genes *in vitro* and during colonization of *Arabidopsis* roots at 10 and 24 d.p.i. under phosphate-deficient conditions (−P: [50 μM]). (**b**,**c**) Overrepresented (yellow to red) and underrepresented transcripts (yellow to blue) are shown as log_2_ (fold changes) relative to the mean expression across all the stages. The red marks represent secreted CAZymes and the black marks indicate involvement in metabolic activities linked to energy storage and exchange (Energy), or degradation of fungal cell walls (FCW), plant cell walls (PCW) or both (F or PCW). For CAZymes acting on PCW, the corresponding plant substrates (cellulose, hemicellulose, hemicellulose and pectin, pectin) are indicated by a colour code. REI, relative expression index.

Both *Ct* and *Ci* genomes encode a very broad range of CAZymes, including large arsenals of pectate lyases, carbohydrate esterases and glycoside hydrolases acting on all major plant cell wall constituents (Fig. 4a, Supplementary Fig. 12 and Supplementary Data 4). However, the number of predicted carbohydrate-binding modules is inflated in *Ct* compared with pathogenic *Colletotrichum* species, especially chitin-binding CBM18 (48 versus 28–40) and CBM50 (57 versus 30–54) modules (Fig. 4a, Supplementary Data 4), though few of the corresponding *Ct* genes were induced *in planta* (Supplementary Fig. 14). These two chitin-binding modules are similarly highly enriched in the genomes of two other non-mycorrhizal root symbionts^19^,^20^ (*Piriformospora indica* and *Harpophora oryzae*; Supplementary Data 4), suggesting this is a genomic signature common to independently evolving root-associated fungal endophytes.

### Dual RNAseq of *Arabidopsis* roots and fungal partners

We report elsewhere that *Ct* promotes *Arabidopsis* growth under phosphate-deficient (−P) but not phosphate-sufficient (+P) conditions and that transfer of radioactive ^33^P from *Ct* hyphae to host plants is strictly regulated by Pi (inorganic phosphate) availability^8^. To compare the transcriptional dynamics of beneficial *Ct* and pathogenic *Ci* during colonization of *Arabidopsis* roots and study the corresponding host responses, we extensively re-analysed the previously created RNA-seq data for the *Ct*-*Arabidopsis* interaction (6, 10, 16 and 24 days post inoculation (d.p.i.), +P: 625 μM, −P: 50 μM; ref. 8) and included new samples for the *Ci*-Arabidopsis interaction (10 and 24 d.p.i., −P: 50 μM) (Supplementary Figs 15 and 16). After mapping Illumina reads to their respective genomes, we obtained expression data for >20,000 *Arabidopsis* genes, 8,613 *Ci* genes and 6,693 *Ct* genes (Supplementary Fig. 17, Supplementary Table 8 and Supplementary Note 9). The expression data were validated using quantitative PCR with reverse transcription (RT–qPCR) with a subset of *Arabidopsis* and *Ct* genes (Supplementary Fig. 18, Supplementary Table 9 and Supplementary Note 10).

### Transcriptional shutdown of pathogenicity genes in *Ct*

Among the 3,885 *Ct* genes significantly regulated (moderated *t*-test, |log_2_FC|⩾1, FDR<0.05), only few (61) were impacted by phosphate status (described in ref. 8) or the fungal developmental stage *in planta* (845; Supplementary Data 5 and Supplementary Fig. 19). In contrast, ∼80% were induced upon host contact and particularly those encoding CAZymes, for which a dynamic expression pattern was observed (Fig. 4b and Supplementary Figs 19 and 20). A first wave of activation (6–16 d.p.i.) involved few plant cell wall-degrading enzymes (PCWDEs) acting mostly on hemicellulose, while a second wave (24 d.p.i.) involved induction of numerous PCWDEs acting on all major wall polymers, including cellulose, hemicellulose and pectin (Fig. 4b). Thus, at later infection stages, *Ct* displays significant saprotrophic capabilities. However, genes encoding CSEPs, secreted proteases, secondary metabolism key enzymes and transporters showed no clear activation (Supplementary Fig. 21), in contrast to the highly stage-specific deployment of such genes by *C. higginsianum* during infection of *Arabidopsis* leaves^2^. Surprisingly, the activation of *Ct CSEP*s was almost non-existent *in planta*, with only 18/133 expressed during colonization, 8/133 induced *in planta* (log_2_FC⩾1) and 4/133 ranking among the 1,000 most highly expressed genes (Fig. 3c). These few expressed *CSEP* genes showed similar *d*_N_/*d*_S_ ratios compared with *CSEP*s that were silent *in planta* (Supplementary Data 3). The contracted repertoire and small number of *CSEP*s activated *in planta* suggests *Ct* requires extremely few effectors for host invasion and maintenance of the beneficial relationship.

### Gene deployment *in planta* reflects fungal lifestyles

To uncover transcriptional adaptations associated with the evolutionary transition from the ancestral pathogenic lifestyle to beneficial endophytism, we compared the normalized expression levels of 6,804 *Ct* and *Ci* orthologous gene pairs that are expressed *in planta* (10, 24 d.p.i.; −P) (Supplementary Data 6). More than twice as many gene pairs were differentially expressed at 10 d.p.i. (621 up, 842 down) than at 24 d.p.i. (306 up, 273 down; moderated *t*-test, |log_2_FC|⩾1, FDR<0.05), suggesting that early colonization events are critical for determining the outcome of the interaction. GO term enrichment analysis showed that processes related to melanin biosynthesis were significantly enriched in *Ct*, consistent with the formation of melanized microsclerotia in *Ct* but not *Ci*^8^ (Supplementary Table 10). We also found major differences between *Ct* and *Ci* in the expression of gene categories typically associated with fungal pathogenicity. *In planta* activation of *CSEPs* was more pronounced in *Ci* compared with *Ct*, with seven times more *CSEPs* highly expressed (top 1,000 expressed genes) and three times more upregulated *in planta* at 10 d.p.i. (Fig. 3c,d and Supplementary Data 5 and 7). Likewise genes encoding CAZymes and secondary metabolism enzymes displayed earlier and stronger transcriptional activation *in planta* and broader diversity in *Ci* (Fig. 4b,c and Supplementary Fig. 22). Consistent with this, we observed a reduced number of living cells and a depletion of beta-linked polysaccharides (including cellulose) from host cell walls in *Ci*-colonized roots at 10 d.p.i., but not in *Ct*-colonized roots (Supplementary Fig. 1). This finding suggests that pathogenic *Ci* harvests carbon from plant cell walls more aggressively than *Ct*. Thus, despite their phylogenetic proximity and similar gene arsenals, gene deployment during infection was strikingly different between *Ct* and *Ci*. The *in planta* transcriptome of *Ci* resembles that of other pathogenic *Colletotrichum* species^2^, whereas the less dynamic transcriptome of *Ct* might contribute to, or be a consequence of, the beneficial relationship. Overall, our results suggest that the recent transition from pathogenic to beneficial lifestyles might be partly controlled through transcriptional downregulation of pathogenicity-related genes in *Ct*.

### Host responses to *Ct* are phosphate-status dependent

**Figure 5:**
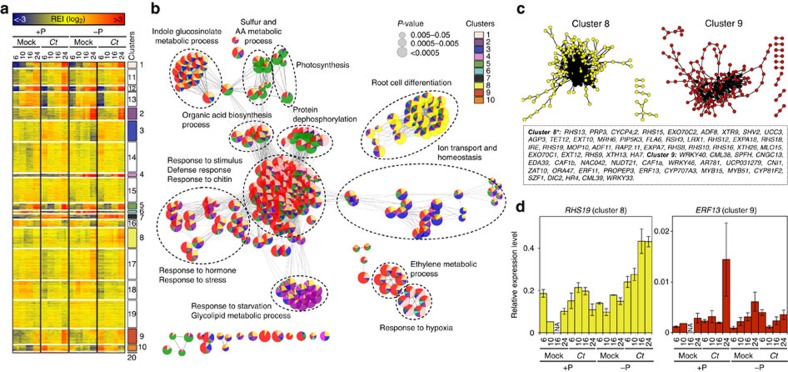
Transcriptional reprogramming of Pi-starved and non-starved *Arabidopsis* roots in response to *C. tofieldiae*. (**a**) Transcript profiling of 5,561 *Arabidopsis* genes significantly regulated (moderated *t*-test, |log_2_FC|⩾1, FDR<0.05) between colonized versus mock-treated roots and phosphate-starved (−P: [50 μM]) versus non-starved roots (+P: [625 μM]) at 6, 10, 16 and 24 days post inoculation. Overrepresented (yellow to red) and underrepresented transcripts (yellow to blue) are shown as log_2_ (fold changes) relative to the mean expression across all stages. Using *k*-means partitioning, the gene set was split into 20 major gene expression clusters. (**b**) Gene Ontology term enrichment network analysis among the 10 clusters highlighted in **a**. Each significantly enriched GO term (*P*<0.05, hypergeometric test, Bonferroni step-down correction) is represented with a circle and the contribution (%) of each cluster to the overall GO term enrichment is represented using the same colour code as in **a**. As tightly connected GO terms are functionally linked, only the major host responses outputs are indicated (dotted line). (**c**) For cluster 8 and cluster 9, gene relationships based on co-regulation were assessed using other *Arabidopsis* expression data sets (see Supplementary Note 9). The genes within each cluster that show strong expression relationships in other expression data sets are likely to encode key regulatory hubs. Hub genes (cluster 9: ⩾5 connections, *cluster 8: ⩾10 connections) are highlighted in black. The corresponding characterized *Arabidopsis* genes are indicated below the co-expression networks. (**d**) Validation of the expression profiles of the hub genes *RHS19* (cluster 8) and *ERF13* (cluster 9) using RT–qPCR (see Supplementary Note 10). Error bars indicate standard error (*n*=3 biological replicates), NA, data not available; REI, Relative Expression Index.

To disentangle how Pi-starved and non-starved *Arabidopsis* roots respond to *Ct* colonization over time, we compared *Ct*-colonized and mock-inoculated roots under +P and −P conditions. In total, 5,661 *Arabidopsis* genes were differentially expressed in at least one of the 16 pair-wise comparisons (moderated *t*-test, |log_2_FC|⩾1, FDR<0.05) and grouped into 20 major gene expression clusters (Fig. 5a and Supplementary Data 8). GO term enrichment analysis among these clusters indicated that the phosphate level used in our study (50 μM) was sufficient to provoke a phosphate starvation response in *Arabidopsis* roots (clusters 2 and 4; Fig. 5b). Furthermore, our analysis indicates that ‘response to stimulus', ‘indole glucosinolate metabolic process', ‘defense response' and ‘ethylene metabolic process' are activated in *Ct*-colonized roots under +P but not −P conditions (cluster 9) (Fig. 5b and Supplementary Data 9). In contrast, the genes related to ‘root cell differentiation' (cluster 8, Fig. 5b) and phosphate uptake^8^ were preferentially activated in Pi-starved *Arabidopsis* roots during *Ct* colonization, similar to mycorrhizal symbiont–host interactions^21^. To identify key regulatory genes (hub genes) that might orchestrate transcriptional reprogramming in the contrasting directions seen in clusters 8 and 9, we checked which of these genes are often co-regulated in other expression data sets using the ATTED-II gene co-expression database (Fig. 5c). Among the hub genes that showed high connectivity within cluster 8 (highlighted with black dots), many encode proteins involved in cell wall remodelling and root hair development. Particularly, genes encoding the root hair-specific proteins RHS8, RHS12, RHS13, RHS15 and RHS19 (ref. 22) are upregulated (moderated *t*-test, |log_2_FC|⩾1, FDR<0.05) in *Ct*-colonized versus mock-treated roots under −P conditions, which was validated by RT–qPCR (Fig. 5d and Supplementary Fig. 18). This expression pattern suggests that *Ct*-dependent remodelling of root architecture might play a key role to enhance phosphate uptake during starvation (Supplementary Note 9). Similarly, we identified 27 hub genes within cluster 9 (Fig. 5c, black dots), encoding well-characterized defense-related proteins such as the transcription factors WRKY33 and WRKY40 (ref. 23), the ethylene-responsive factors ERF11 and ERF13 (ref. 24), as well as MYB51 (ref. 25), a transcription factor regulating Tryptophan (Trp)-derived indole glucosinolate metabolism. Four other genes involved in indole glucosinolate metabolism were also highly differentially regulated in cluster 9, including the myrosinase *PEN2* and the P450 monooxygenase *CYP81F2* required for the biosynthesis of 4-methoxy-indol-3-ylmethylglucosinolate, the substrate of PEN2 myrosinase^26^,^27^ (Supplementary Data 9). The PEN2-dependent metabolism of Trp-derived indole glucosinolates in *A. thaliana* is activated upon perception of pathogen-associated molecular patterns by receptors of the innate immune system and is needed for broad-spectrum defence to restrict the growth of fungal pathogens^26^,^27^. Notably, in *Arabidopsis* mutants that cannot activate PEN2-mediated antifungal defense, the promotion of plant growth by *Ct* is impaired, while the depletion of all Trp-derived secondary metabolites renders *Ct* a pathogen on *Arabidopsis*^8^. These findings strongly suggest that the phosphate starvation response and Trp-derived indole glucosinolate metabolism are interconnected to control fungal colonization of *Arabidopsis* roots^28^. Phosphate status-dependent activation of defense responses was also observed among the 411 expressed *Arabidopsis* genes annotated as ‘chitin-responsive' (Supplementary Fig. 23), based on GO term enrichment among all significantly regulated genes (Supplementary Fig. 24) and this was validated by RT–qPCR (Fig. 5d and Supplementary Fig. 18). These data reveal a remarkable capacity of *Arabidopsis* roots to prioritize different transcriptional outputs in response to *Ct*, favouring either defense responses under +P conditions or root growth and phosphate metabolism under −P conditions.

### Phosphate-starved roots activate defense responses to *Ci*

**Figure 6:**
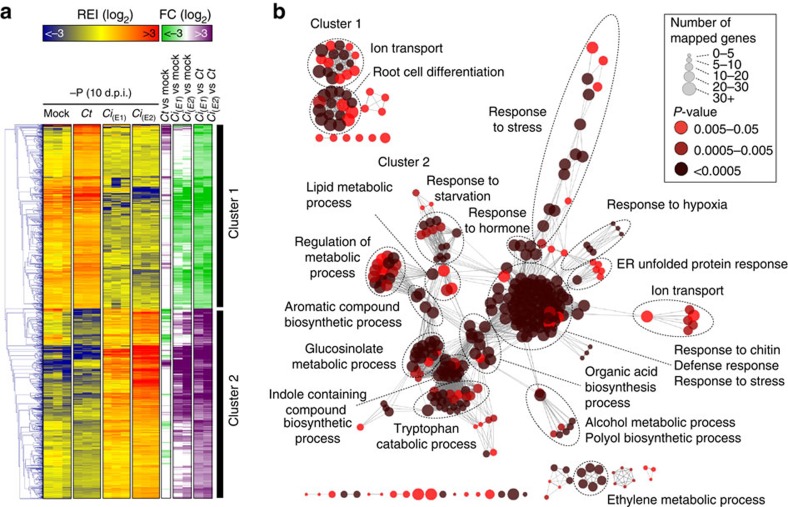
Comparative transcriptome analysis of *Arabidopsis* roots in response to beneficial *C. tofieldiae* and pathogenic *C. incanum.* (**a**) Transcript profiling of 2,009 *Arabidopsis* genes significantly regulated (moderated *t*-test, |log_2_FC|⩾1, FDR<0.05) between *C. incanum*- versus (vs) *C. tofieldiae*-colonized roots at 10 days post inoculation (d.p.i.) under phosphate-deficient conditions (−P: 50 μM). Overrepresented (yellow to red) and underrepresented transcripts (yellow to blue) are shown as log_2_ (fold changes) relative to the mean expression across all stages. E1 and E2 correspond to two fully independent experiments (see Supplementary Note 9). Gene expression fold changes (green: downregulated; violet: upregulated) were calculated between *C. tofieldiae*-colonized versus mock-treated roots, *C. incanum*-colonized versus mock-treated roots or *C. incanum*-colonized versus *C. tofieldiae*-colonized roots. (**b**) GO term enrichment analysis of *Arabidopsis* genes preferentially expressed in response to *C. tofieldiae* (Cluster 1) or in response to *C. incanum* (cluster 2). Each circle corresponds to a significantly enriched GO term (*P*<0.05, hypergeometric test, Bonferroni step-down correction). The colour code reflects *P* values and the circle size the number of genes associated to each GO term. Similar to Fig. 5b, the GO terms that are tightly connected are functionally linked and therefore only the major host-response outputs are indicated (dotted line). REI, Relative Expression Index.

To clarify whether the reduced activation of defense responses observed in *Ct*-colonized roots under −P conditions is not simply due to phosphate deficiency, we compared the transcriptomes of Pi-starved *Arabidopsis* roots in response to either *Ci* or *Ct* at 10 d.p.i. In total, 2,009 differentially expressed genes were identified (moderated *t*-test, |log_2_FC|⩾1, FDR<0.05), including 988 genes induced in *Ct*-colonized roots (cluster 1) and 1,021 genes in *Ci*-colonized roots (cluster 2; Fig. 6a and Supplementary Data 10). GO term enrichment analysis revealed that ion transport and root cell differentiation mechanisms were activated in *Ct*-colonized roots, whereas strong defense responses were triggered in *Ci*-colonized roots (Fig. 6b). Thus, although Pi-starved *Arabidopsis* roots remain able to mount immune responses against pathogenic *Ci*, transport and root growth are instead prioritized during interaction with beneficial *Ct*.

## Discussion

Deciphering the genetic basis of the transition from pathogenic to beneficial plant-fungal interactions is crucial for a better understanding of the evolutionary history of fungal lifestyles^20^,^29^. It was recently shown that the ectomycorrhizal lifestyle arose independently multiple times during evolution and that the transition was associated with (1) convergent loss of genes encoding PCWDEs present in their saprotrophic ancestors and (2) the repeated evolution of lineage-specific ‘toolkits' of mycorrhiza-induced genes^15^. However in striking contrast with ectomycorrhizal fungi, this transition in *Ct*, *P. indica* and *H. oryzae* was not accompanied by contraction of their PCWDE repertoires^19^,^20^. In our study, the close phylogenetic relatedness of beneficial *Ct* and pathogenic *Ci*, and their ability to infect the same plant host, allowed us to resolve both genomic and transcriptomic signatures associated with this evolutionary transition. The overall high genomic similarity between *Ct* and *Ci* suggests that this transition involved only subtle remodelling of the gene repertoire (that is, a reduced set of CSEPs and expansion of chitin-binding and secondary metabolism-related protein families). The retention of abundant pathogenicity- or saprotrophy-related genes implies that they are still needed by *Ct*, perhaps for exploitation of other plant hosts or during plant senescence when *Arabidopsis* leaves are extensively colonized by *Ct* mycelium^8^. Our results also suggest that changes in fungal gene expression patterns during host colonization, rather than extensive remodelling of the gene repertoire, provides an alternative and probably transient adaptation to a beneficial endophytic lifestyle. This may reflect the relatively recent transition from pathogenic to non-pathogenic lifestyles in *Ct* and, consequently, a latent capacity to revert to a pathogenic lifestyle.

During the last decade, the molecular mechanisms by which plants respond to colonization by pathogenic or mutualistic fungi have been extensively studied^30^. However, it remains unclear how plants discriminate and respond appropriately to closely related fungal partners with different lifestyles. The sedentary nature of plants suggests they have evolved regulatory systems to integrate exposure to conflicting biotic and abiotic stresses and balance their resource allocation strategically to maximize growth and survival. A recent report showed that plant responses to multiple stresses are not cumulative and suggested that prioritization of stress responses does take place^31^. For plant–mycorrhizal associations, an inverse correlation was observed between phosphate levels and the number of arbuscules formed in roots^32^. Although the detailed molecular mechanism remains unclear, this suggests that the nutritional status of the plant impacts fungal colonization efficiency. Here, we show that host transcriptional responses to *Ct* are dependent on phosphate availability, with defense responses activated or suppressed under high- or low-phosphate conditions, respectively. The fact that immune responses are retained in phosphate-starved roots colonized by *Ci* makes it unlikely that metabolic competition between phosphate starvation and defense response systems attenuates defense gene activation during interactions with *Ct* under P-limiting conditions. Recently, a metabolic link between the phosphate starvation response and glucosinolate biosynthesis was described^28^ and the functional relevance of this link is supported by our observation that *Ct*-mediated plant growth promotion is impaired in *Arabidopsis* mutants lacking regulatory components of indole glucosinolate metabolism or the phosphate starvation response^8^. Therefore, we hypothesize that connectivity between nutrient sensing and innate immunity systems in the host, combined with subtle genomic adaptations in *Ct*, has enabled the transition from pathogenic to beneficial *Arabidopsis*–*Colletotrichum* interactions (Supplementary Fig. 25). Consequently, the interaction with beneficial *Ct*, but not with pathogenic *Ci*, is tightly controlled in plant roots by trade-offs between nutrition and defense. Whether phosphate stress-dependent defense attenuation renders *Ct*-colonized plants super-susceptible to other microbial pathogens remains to be tested. Our results are consistent with the fact that transfer of Pi from ramifying fungal hyphae to roots, and subsequent allocation to shoots for plant growth, occurs only under phosphate-deficient conditions^8^. Notably, where *Ct* naturally associates with *Arabidopsis* in central Spain, the level of bioavailable phosphate in soil at those locations is very low (5.5 to 17 p.p.m., Supplementary Table 11). Our findings suggest that both innate immune responses (that is, indole glucosinolate metabolism) and soil phosphate availability are important selective forces driving fungal adaptation and contributing to the evolutionary transition from parasitic to beneficial *Arabidopsis*–fungal associations.

## Methods

### Genome sequencing and assembly

*C. incanum* and the five *C. tofiediae* isolates were grown in liquid Mathur's medium (2.8 g glucose, 1.22 g MgSO_4_.7H_2_O, 2.72 g KH_2_PO_4_ and 2.18 g Oxoid mycological peptone in 1 l deionized water) supplemented with 100 μg ml^−1^ rifampicin and 125 μg ml^−1^ streptomycin. Genomic DNA was isolated using the DNeasy Plant Mini Kit (Qiagen) from 100 mg of fungal mycelium. Library construction, quality control and DNA sequencing for 454 GFLX+ or Illumina Hiseq sequencing were performed at the Max Planck Genome Centre Cologne (http://mpgc.mpipz.mpg.de) using 1 μg genomic DNA. After the preparation of genomic DNA libraries, 454 reads (557 bp on average) and Illumina paired-end reads (100 bp) were obtained from Roche 454 FLX+ and Illumina HiSeq2500 sequencers, respectively. For the *Ct*0861 reference genome, a hybrid assembly strategy was used combining 454 and Illumina data. Unpaired 454 reads were first assembled using MIRA 4.0 (ref. 33) and filtered MIRA-contigs (>5,000 bp) were further used for scaffolding of Illumina paired read assemblies from SPAdes 3.0 (ref. 34). The established SPAdes 3.0 pipeline was used in ‘careful' mode providing 454 MIRA assemblies as untrusted-contigs for scaffolding only and a kmer scan using 21, 31, 41, 61, 75 and 81. All other assemblies were constructed only from Illumina data using a combination of VELVET 1.2.1 (ref. 35) and SPAdes^34^. Using BLASTN searches, contigs were identified that were missing from combined SPAdes assemblies but present in VELVET assemblies. To integrate those contigs and extend further where possible, SPAdes was re-run as described above but in ‘trusted-contigs' mode where trusted contigs were provided as fasta files with absent contigs only. All the assemblies were generated using ‘careful' mode in SPAdes to avoid miss-pairing of contigs by scaffolding and for further analyses, contigs <100 bp were removed. To identify and remove potential contaminating sequences, assemblies were aligned to the genomes of *A. thaliana*, *H. sapiens* and PhiX (sequencing spike-in control) using MUMmer^36^ with default parameter settings. Contigs that aligned with more than 50% of their sequence (coverage; ‘COV') and at least 85% sequence identity (‘IDY') to any of the tested contaminants were removed from the assemblies. In addition, contigs that aligned with 75–85% identity (and >50% coverage) or with 10–50% coverage (and >85% identity) were also removed, if the judgment of the sequence being non-fungal was confirmed through BLASTN searches in the NCBI nr database (with default settings). For the *Ct*0861 assembly, RNA-sequencing data were used for further clean-up. Finally, assembly quality was assessed on the basis of L50/75/90 and N50/75/90 values, percentage of error-free bases estimated with REAPR^37^ (version 1.0.16, default settings) and gene space coverage estimated with CEGMA^38^ (version 2.0, default settings).

### Repetitive DNA analysis

We identified repetitive DNA in the genome assemblies using either *de novo* or homology approaches. For *de novo* searches, we used PILER and PALS^39^ to identify repetitive sequences and classify them into families. The resulting libraries of consensus sequences were then used to scan the genome sequences using RepeatMasker^40^ (version 4.0.3) to identify individual repetitive elements. For homology-based searches, we used RepeatMasker using a library of all fungal elements in the Repbase database^41^ (version 20140131).

### Phylogeny and divergence date estimation

All phylogenetic analyses performed in this study are described in the Supplementary Note 11. For evolutionary divergence date estimation, clustering, protein family selection and phylogenetic analyses were performed with scripts in the Mirlo package (https://github.com/mthon/mirlo). The phylogeny was calibrated using the penalized-likelihood method implemented in r8s (ref. 42) using one primary and two secondary calibration points (Supplementary Note 11).

### Short-read alignment and SNP analysis

To compare the genome sequences of *Ct* isolates, Illumina short reads of the four other isolates were mapped onto the genome assembly of *Ct*0861 using Bowtie2 (ref. 43) (default settings for paired-end data). Subsequently, duplicate reads were removed using the rmdups function from the SAMtools toolkit^44^ (default settings). On the basis of the mapped genome sequencing reads, single-nucleotide polymorphisms (SNPs) were identified using the mpileup function in SAMtools^44^ (version 0.1.18; with option -u) The obtained SNP sets were filtered by applying the bcftools script vcfutils.pl varFilter (SAMtools) with adjusted read depth settings according to the respective sequencing read coverage to -d 80 and -D 800 for CBS495 and to -d 40 and -D 400 for CBS130, CBS127 and CBS168. The SNP locations, read coverage for each isolate and locations of conserved regions were visualized using the Circos software package^45^ (version 0.62.1). In addition, we also calculated SNP densities (SNPs per kb) relative to *Ct*0861 for each isolate as a function of the genomic location on all *Ct*0861 contigs larger than 50 kb, using a 10-kb sliding window that moved 1 kb at each step. For visualization of the SNP densities, these windows were sorted in the increasing order by contig number and position on the contig. To identify windows with a low SNP density, that is, a common haplogroup, between isolates we classified the SNP density in each window as either ‘low' or ‘high' using a two-state hidden Markov model (HMM). This HMM was created and fitted on the observed 10 kb SNP densities by the expectation-maximization algorithm using functions ‘depmix' and ‘fit' (R package depmixS4), and subsequently the posterior state sequence (with states ‘low' and ‘high'), computed via the Viterbi algorithm, was extracted with function ‘posterior' (R package depmixS4).

### Gene annotation

The prediction of *Ct* and *Ci* gene models was performed using the MAKER pipeline^46^ (version 2.28) , which integrates different *ab initio* gene prediction tools together with evidence from EST and protein alignments. In a first step, for each genome, the pipeline was run using Augustus^47^ (with species model *Fusarium graminearum*) and GeneMark-ES^48^ for *ab initio* gene prediction together with transcript and protein alignment evidence. The resulting gene models from this first run were used as training set for a third *ab initio* prediction tool, SNAP^49^, and subsequently the annotation pipeline was re-run, this time including all three *ab initio* prediction tools together with the transcript and protein alignment evidence to yield the final gene models. The alignment evidence was created from BLAST and Exonerate^50^ alignments of both protein and transcript sequences of each respective fungus (*Ct*/*Ci*) and protein sequences of *C. higginsianum* and *C. graminicola*. *Ct* (isolate 0861) and *Ci* transcript and protein sequences were obtained from the corresponding RNA-seq data via a transcriptome *de novo* assembly. For this purpose, we extracted all RNA-seq read pairs that did not align to the host plant genome from four (*Ct*) to nine (*Ci*) *in planta* samples and combined these with the read pairs from one *in vitro* sample of the respective fungus. The combined RNA-seq reads were then used as input for Trinity^51^ (with default parameter settings for paired-end reads) to assemble transcripts and extract peptide sequences of the best-scoring ORFs (using the Perl script ‘transcripts_to_best_scoring_ORFs.pl' provided with the Trinity software). General functional annotations for the predicted gene models were obtained using Blast2GO (ref. 52). To perform Blast2GO searches and ensure stable databases over time for multiple genome annotations, the NCBI nr database was downloaded locally (version: 8 January 2015). In addition, a local b2gdb mysql database was generated (version 201402) and connected to the Blast2GO java tool. For each genome annotation, BLASTP was performed against the local NCBI nr database (−e 1E−3, -v 10 −b 10) and tabular BLAST output was loaded into Blast2GO using graphical java interface. Further analyses were performed according to the Blast2GO user manual.

### MCL analysis

Gene families and clusters of orthologous genes were inferred using OrthoMCL^53^ (version 2.0) with standard parameters and granularity 1.5 for the MCL clustering step. Functional enrichment and overrepresentation analyses were performed using a Fisher's exact test, adjusting for FDR. For each gene family inferred with orthoMCL, a multiple sequence alignment of the protein sequences was obtained using Clustal Omega^54^ and an HMM model was generated with the hhmake program of the HHSuite toolkit^55^. Sequences from the fungal database fuNOG^56^ were similarly aligned and HMM models generated. To annotate whole gene families, the hhsearch program was used to obtain matches between the gene family and the fuNOG HMMs and only hits with a probability equal to or higher than 0.99 were considered. To annotate whole gene families, the hhsearch program was used to obtain matches between the gene family and the fuNOG HMMs and only hits with a probability ⩾0.99 were considered.

### Ancestral genome reconstruction

Gene families inferred with OrthoMCL were used to reconstruct the ancestral genomes of each *Colletotrichum* lineage. GLOOME^57^ (maximum-likelihood approach) was used to infer ancestral gene gains and losses (GGLs) and to reconstruct the ancestral GGLs of gene families on the species tree of *Ct*0861 and the other five genomes available for this genus. Evolution of the GGLs along the branches of a phylogenetic tree was modelled as a continuous time Markov process using a binary character alphabet corresponding to gene family presence or absence. Default parameters were used, corresponding to a mixture model that allows varying GGL rates across gene families. We approximated the total number of gene families that were gained or lost on a branch by summing up the individual posterior probabilities for each gene family to be gained or lost on that branch and rounding this number to the closest integer. The number of genes either gained or lost (annotated with one specific category) was compared with the respective numbers detected for all other branches of the tree. The significance was assessed using Fisher's exact test and FDR corrected.

### *d*_N_/*d*_S_ analysis

A multiple-sequence alignment (MSA) of orthologous groups of coding sequences (CDSs) was created with Clearcut^58^. Based on the MSA and the CDSs, a codon alignment was constructed for each protein family with pal2nal (ref. 59; version 14) using default parameters. Because of the data set size and the shorter runtime of neighbour joining algorithms compared with maximum-likelihood methods, Clearcut, a relaxed neighbour joining algorithm^58^, was chosen for reconstructing phylogenetic trees from the MSA of each protein family with slightly modified additive pairwise distances whereby gaps are not counted as mismatches. Gaps in this alignment were mostly of technical origin due to the alignment of short contigs to longer reference sequences. Using an in-house tool (phylorecon), CDSs and amino acid sequences were reconstructed for the internal nodes of each phylogenetic tree using maximum parsimony as a criterion^60^, and the synonymous and non-synonymous substitution rates per site were inferred with correction for multiple substitutions. The average *d*_N_/*d*_S_ ratio was calculated for each protein family and a one-sided Fisher's test (FDR corrected) was performed to identify protein families with a significant enrichment of synonymous mutations per synonymous site versus non-synonymous mutations per non-synonymous site.

### Annotation of specific gene categories

Secretomes of all species were predicted using WoLF-PSORT^61^ with default settings. *Colletotrichum* CSEPs were defined as extracellular proteins with no significant BLAST homology (*E*-value <1 × 10^−3^) to sequences outside the genus *Colletotrichum* in the UniProt database (SwissProt and TrEMBL components). To identify secreted proteases, sequences of predicted extracellular proteins were subjected to a MEROPS Batch BLAST analysis^62^. Membrane transporters were identified and classified through BLAST searches against the Transporter Collection Database (http://www.tcdb.org/). To predict the repertoire of carbohydrate-active enzymes encoded by *Colletotrichum* species, we scanned their genomes using the CAZy annotation pipeline^63^ (http://www.cazy.org). For annotating genes encoding secondary metabolism key enzymes in *Colletotrichum* species, we used an in-house bioinformatics pipeline that was developed as described in Supplementary Note 12.

### RNA sequencing

The RNA-seq samples presented in Hiruma *et al*.^8^ and the new samples presented here were prepared as follows. Fungal cultures were maintained on Mathur's agar medium at 25°C, and conidia were harvested from 7- to 10-day-old cultures. For sample preparation, *Arabidopsis* Col-0 seeds were surface sterilized in 70% ethanol and subsequently in 2% hypochlorous acid (v/v) containing 0.05% (v/v) Triton. We inoculated *A. thaliana* Col-0 seeds with spores (5 × 10^4^ spores ml^−1^) of *Ct* 0861 or *Ci* and transferred the inoculated seeds onto solid half-strength Murashige and Skoog medium (pH=5.1) either in normal [625 μM] or low phosphate [50 μM] conditions. For each biological replicate (*n*=3), the entire root system of at least 10 plants was collected at time intervals (6, 10, 16 or 24 d.p.i.) and pooled before RNA extraction. In addition, we grew *Ct* and *Ci* in liquid Mathur's medium (*in vitro* samples) for 2 days at 24 °C with shaking at 50 r.p.m. and collected the hyphae by filtration. Total RNA was purified with the NucleoSpin RNA plant kit (Macherey-Nagel) according to the manufacturer's protocol. RNA-seq libraries were prepared from an input of 1 μg total RNA using the Illumina TruSeq stranded RNA sample preparation kit. Libraries were subjected to paired-end sequencing (100 bp reads) using the Illumina HiSeq2500 Sequencing System. To make sure the sequenced reads were of sufficiently high quality, an initial quality check was performed using the FastQC suite (http://www.bioinformatics.babraham.ac.uk/projects/fastqc/). Subsequently, the RNA-seq reads were mapped to the assembled and annotated genomes of either *Ct* 0861 or *Ci*, and in parallel to the annotated genome of the host plant *A. thaliana* (TAIR10) using Tophat2 (ref. 64; *a*=10, *g*=10, *r*=100, mate-std-dev=40). The mapped RNA-seq reads were then transformed into a fragment count per gene per sample using the htseq-count script (s=reverse, t=exon) in the package HTSeq^65^. The complete RNA-Seq data presented by Hiruma *et al*.^8^ and in this manuscript have been deposited under the GEO series accession number GSE70094.

### Statistical analysis of differential gene expression

All statistical analyses of plant and fungal gene expression were performed in R (codes are available upon request). For the analyses of plant gene expression, genes with less than 100 mapped fragments in total (that is, across all the analysed samples) were rated as ‘not expressed' and therefore excluded. For analyses of fungal gene expression, we excluded genes that were not sufficiently expressed in the *in planta* samples, that is, genes with less than 100 (*Ct*, 24 samples) or less than 50 (*Ci*, 6 samples) mapped fragments across all the analysed samples. Subsequently, the count data for all expressed genes was TMM-normalized and log-transformed using the functions ‘calcNormFactors' (R package EdgeR^66^) and ‘voom' (R package limma^67^) to yield log_2_ counts per million (log_2_cpm). To analyse the aspects of differential gene expression in *Ct*0861, *Ci* and their host plant *Arabidopsis*, we fitted for each analysis a distinct linear model to the respective log_2_-transformed count data using the function lmFit (R package limma^67^) and subsequently performed moderated *t*-tests for specific comparisons of interest. Resulting *P* values were adjusted for false discoveries due to multiple hypotheses testing via the Benjamini–Hochberg procedure (FDR). To extract genes with significant expression differences, a cutoff of FDR<0.05 and |log_2_FC|⩾1 was applied. Heatmaps of gene expression profiles were generated with the Genesis expression analysis package^68^ and interactive Tree Of Life^69^ was used to visualize CSEP gene expression data. To derive *Arabidopsis*, *Ct* and *Ci* gene expression profiles during the time-course experiment, log_2_ expression ratios were calculated between the normalized number of reads detected for a given gene at a given developmental stage and the geometrical mean of the number of reads calculated across all developmental stages. This log_2_ ratio is referred as the ‘Relative Expression Index'. The Cytoscape plug-in ClueGO+CluePedia^70^ was used to construct GO term enrichment networks and to visualize functionally grouped terms among significantly regulated genes. Significant enrichments were determined using the hypergeometric test and Bonferroni step-down corrected *P* values are represented. Co-regulated genes that were also co-expressed in other *Arabidopsis* expression data sets were identified using ATTED-II (http://atted.jp/) and co-expression networks were generated using Cytoscape^71^ (version 3.1.1).

### RT–qPCR analysis

First-strand cDNA was synthesized from 1 μg DNase-treated total RNA using the iScript cDNA synthesis kit (Bio-Rad) and PCR amplification was performed using the iQ5 real-time PCR detection system (Bio-Rad). For each gene, specific primers were designed with the Primer 3 and AmplifX programs. BLASTN searches against the *Ct* and *A. thaliana* genomes were performed to rule out cross-annealing artefacts. Gene expression levels were normalized using the reference gene actin (*ACT2*, AT3G18780) for *A. thaliana* and the reference gene tubulin beta-1 chain (CT04_12898) for *Ct*. These genes were used to normalize gene expression levels using the Pfaffl calculation method^72^.

### Microscopy methods

For cytology experiments, surface-sterilized *A. thaliana* Col-0 seeds were inoculated with either *Ct* or *Ci* conidia (5 × 10^4^ spores ml^−1^). The seeds were then transferred to half-strength Murashige and Skoog agarose medium without sucrose and low-phosphate content (50 μM). Inoculated plants were grown at 22 °C with a 10-h photoperiod (80 μE m^−2^ s^−1^) for 1 to 24 days. The roots were either mounted in water for viewing GFP or first stained with Calcofluor white (0.01 %, Sigma) or fluorescein diacetate (10 μg ml^−1^, Sigma). For visualizing GFP and FDA fluorescence, we used an Olympus FV1000 confocal microscope equipped with dry × 20 and × 40 objectives, using the 488-nm line of an Argon laser for excitation and fluorescence was collected at 490–520 nm. For imaging Calcofluor fluorescence, we used a Zeiss Axiophot epifluorescence microscope (filter set BP 365, FT 395, LP 397).

## Additional information

**Accession codes:** The genome assemblies have been deposited at DDBJ/EMBL/GenBank with accession numbers LFIW01000000 (*Ci*), LFIV01000000 (*Ct*0861), LFHR01000000 (CBS127), LFHS01000000 (CBS130), LFHP01000000 (CBS495), LFHRQ01000000 (CBS168). The RNA-Seq data have been deposited in the NCBI Gene Expression Omnibus under GEO Series accession number GSE70094.

**How to cite this article:** Hacquard, S. *et al*. Survival trade-offs in plant roots during colonization by closely related beneficial and pathogenic fungi. *Nat. Commun.* 7:11362 doi: 10.1038/ncomms11362 (2016).

## Supplementary Material

Supplementary Information Supplementary Figures 1-25, Supplementary Tables 1-11, Supplementary Notes 1-12 and Supplementary References

Supplementary Data Set 1 Gene families that were predicted to be either gained (blue) or lost (red) on the branch leading to C. *incanum* or gained (green) or lost (yellow) on the branch leading to C. *tofieldiae*. The color coding is according to the most probable scenario.

Supplementary Data Set 2 Protein families that were significantly enriched in nonsynonymous substitutions (p-value < 0.001) in the five pathogenic *Colletotrichum* species (left) and in the five C. *tofieldiae* isolates (right) grouped in fuNOG categories on level 3 (lvl3). If one description was present multiple times, the median of the p-values was taken.

Supplementary Data Set 3 Expression, regulation and evolution of C. *tofieldiae* and C. *incanum* genes encoding candidate secreted effector proteins.

Supplementary Data Set 4 Number of Carbohydrate-Active Enzyme modules of *Colletotrichum* *tofieldiae*, C. *incanum*, C. *graminicola*, C. *higginsianum*, C. *fructicola*, C. *orbiculare* and of other mutualistic or endophytic fungal species determined according to the CAZy database.

Supplementary Data Set 5 Summary of differential gene expression in C. *tofieldiae* isolate 0861 between in vitro and in planta and during colonization of Arabidopsis roots under phosphate sufficient and deficient conditions.

Supplementary Data Set 6 Differential expression between C. *tofieldiae* (0861) and C. *incanum* orthologous genes

Supplementary Data Set 7 Summary of C. *incanum* differential gene expression between in vitro and in planta (during colonization of Arabidopsis roots under phosphate deficient conditions) samples

Supplementary Data Set 8 Summary of A. thaliana differential gene expression during C. *tofieldiae* colonization under phosphate sufficient and deficient conditions

Supplementary Data Set 9 GOterm enrichment network among 10 clusters of A. thaliana genes significantly regulated during phosphate starvation and/or C. *tofieldiae* colonization. The 15 GO terms that showed the higher enrichment in Cluster 9 are highlighted in grey.

Supplementary Data Set 10 A. thaliana genes differentially regulated in response to C. *incanum* (Ci) or C. *tofieldiae* (Ct) at 10 dpi. The 2,009 genes that show consistant differential transcripts accumulation between Ci-colonized and Ct-colonized roots in two independant experiments (E1 and E2, 3 biological replicate each) are presented .
