# Erratum: Survival trade-offs in plant roots during colonization by closely related beneficial and pathogenic fungi

**DOI:** 10.1038/ncomms13072

**Published:** 2016-09-29

**Authors:** Stéphane Hacquard, Barbara Kracher, Kei Hiruma, Philipp C. Münch, Ruben Garrido-Oter, Michael R. Thon, Aaron Weimann, Ulrike Damm, Jean-Félix Dallery, Matthieu Hainaut, Bernard Henrissat, Olivier Lespinet, Soledad Sacristán, Emiel Ver Loren van Themaat, Eric Kemen, Alice C. McHardy, Paul Schulze-Lefert, Richard J. O'Connell

Nature Communications 7 Article number:11362 10.1038/ncomms11362 (2016); Published 05 06 2016; Updated 09 29 2016.

The HTML version of this Article previously published omitted Supplementary Data 8. This has now been corrected in the HTML version of the Article; the PDF was correct from the time of publication.

