# Supplementary material for: Survival trade-offs in plant roots during colonization by closely related beneficial and pathogenic fungi
Source: Nat Commun. 2016 May 6;7:11362. doi: 10.1038/ncomms11362 (PMC4859067; doi:10.1038/ncomms11362)
Supplement: Supplementary Information — Supplementary Figures 1-25, Supplementary Tables 1-11, Supplementary Notes 1-12 and Supplementary References [file ncomms11362-s1.pdf]

Supplementary Fig. 1: Colonization of *Arabidopsis* roots by *C. tofieldiae* and *C. incanum* and their impact on plant growth.

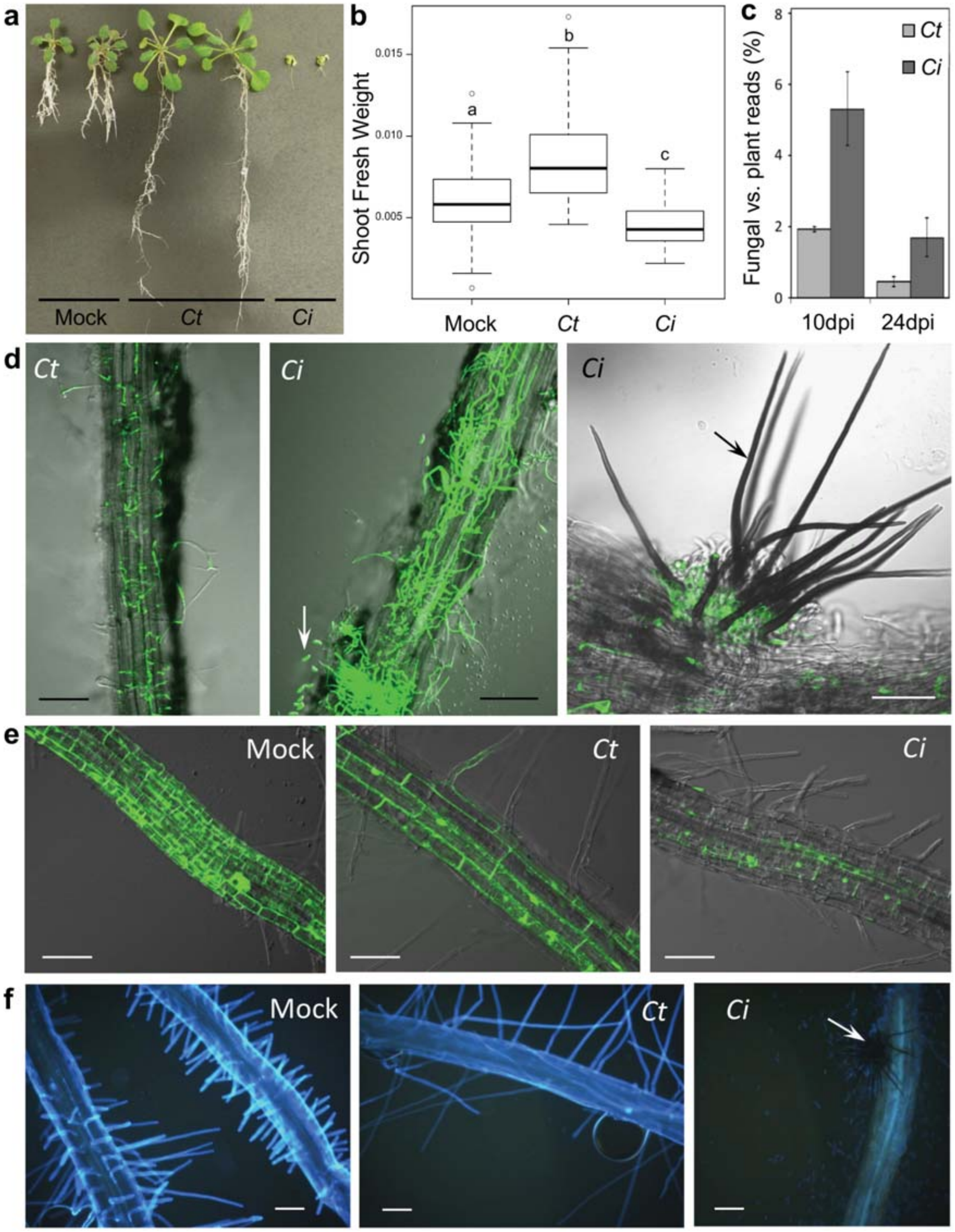

(a) Representative pictures of mock-inoculated *Arabidopsis* plants (mock) and plants colonized by *C. tofieldiae* (*Ct*) and *C. incanum* (*Ci*) grown under low phosphate conditions (50 µM) at 24 days post inoculation. (b) Impact of *Ct* and *Ci* on *Arabidopsis* growth 24 days post-inoculation (dpi). Surface sterilized *Arabidopsis* seeds (Col-0) were treated with spore suspension (5×10^4 spores/ml) and

transferred to solid half-strength Murashige and Skoog medium (MS, pH=5.1) with low phosphate concentration (50 µM). Mock: Mock-treated plants. The shoot fresh weight is presented. Alphabetical letters indicate significant differences (tukey HSD test, $p<0.01$). Similar results are shown in Hiruma *et al*.[1]. (**c**) Percentage of fungal vs. plant RNAseq reads in *Ct*- and *Ci*-colonized roots at 10 and 24 dpi. The higher percentage of *Ci* reads *in planta* suggests that the *in planta* biomass of pathogenic *Ci* is three times higher than the biomass of the beneficial *Ct*. Note that the proportion of fungal vs. plant reads decreases at 24 dpi compared to 10dpi, suggesting that newly formed roots are nor extensively colonized by *Ct* and *Ci.* (**d**) Confocal microscope images of *Arabidopsis* roots colonized by *Ct* or *Ci*, both expressing cytoplasmic GFP, grown under low phosphate conditions (50 µM) at 7 days post inoculation. Note the much more extensive colonization of root tissues by *Ci* at this time-point. White arrow indicates conidia produced by *Ci*. Bars = 100 µm. Right: Asexual sporulation by *Ci* on root surface, forming an acervulus with long melanized setae (black arrow). Bar = 30 µm. (**e**) Confocal microscope images of mock-inoculated *Arabidopsis* roots and roots colonized by *Ct* and *Ci* grown under low phosphate conditions (50 µM) at 10 days post inoculation. Roots were stained with fluorescein diacetate (10 µg ml^{-1}) to label living plant cells green. Note only a small number of host cells remain viable in *Ci*-colonized roots at this time-point. Bars = 100 µm. (**f**) Epi-fluorescence microscope images of mock-inoculated *Arabidopsis* roots and roots colonized by *Ct* and *Ci* grown under low phosphate conditions (50 µM) at 10 days post inoculation. Roots were stained with Calcofluor White (0.01 %) to label beta-linked polysaccharide (including cellulose) in plant cell walls blue. Note the massive removal of cellulose from the walls of *Ci*-colonized roots at this time-point. Identical camera exposure settings were used to capture all images. Right: White arrow indicates an acervulus formed on *Ci*-colonized root. Bars = 100 µm.

Supplementary Fig. 2: Phylogeny of *Colletotrichum* fungi

a

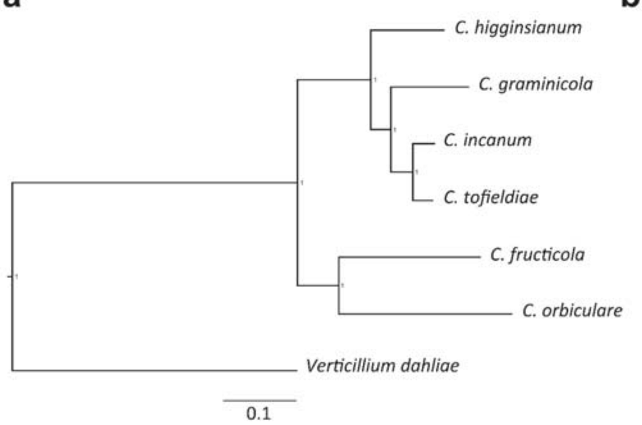

b

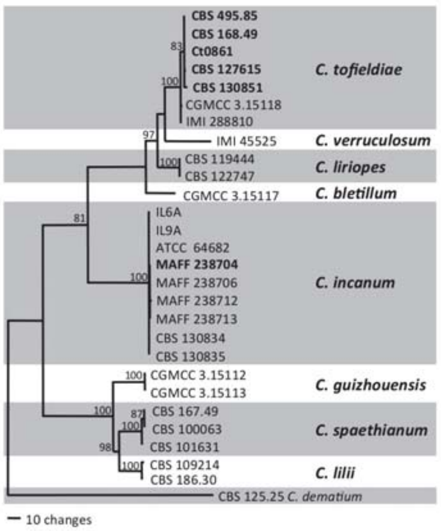

c

Species	Accession number#	Host plant	Country	GenBank accession numbers						Reference
				ITS	TUB2	ACT	HIS3	CHS-1	GAPDH	
C. bletillum	CGMCC 3.15117*	*Bletilla ochracea*	China	JX625178	JX625207	KC843542	-	-	KC843506	Tao et al. (2013)
C. dematium	CBS 125.25*	*Eryngium campestre*	France	GU227819	GU228113	GU227917	GU228015	GU228309	GU228211	Damm et al. (2009)
C. guizhouensis	CGMCC 3.15112*	*Bletilla ochracea*	China	JX625158	JX625185	KC843536	-	-	KC843507	Tao et al. (2013)
C. guizhouensis	CGMCC 3.15113	*Bletilla ochracea*	China	JX625164	JX625192	KC843537	-	-	KC843508	Tao et al. (2013)
C. incanum	IL6a, NRRL 62592, CBS 133485*	*Glycine max*	USA	KC110787	KC110814	KC110823	KC110796	-	KC110805	Yang et al. (2014)
C. incanum	IL9A	*Glycine max*	USA	KC110788	KC110815	KC110824	KC110797	-	KC110806	Yang et al. (2014)
C. incanum	ATCC 64682	*Phaseolus vulgaris*	Canada	KC110789	KC110816	KC110825	KC110798	-	KC110807	Yang et al. (2014)
C. incanum	**MAFF 238704**	*Raphanus sativus*	Japan	sequences retrieved from NIAS Genebank (www.gene.affrc.go.jp)						Sato et al. (2005)
C. incanum	MAFF 238706	*Raphanus sativus*	Japan	sequences retrieved from NIAS Genebank (www.gene.affrc.go.jp)						Sato et al. (2005)
C. incanum	MAFF 238712	*Raphanus sativus*	Japan	sequences retrieved from NIAS Genebank (www.gene.affrc.go.jp)						Sato et al. (2005)
C. incanum	MAFF 238713	*Raphanus sativus*	Japan	sequences retrieved from NIAS Genebank (www.gene.affrc.go.jp)						Sato et al. (2005)
C. incanum	CBS 130834, Ft 12	*Solanum lycopersicum*	USA	KR003336	KR003341	KR003346	KR003351	KR003356	KR003361	This study
C. incanum	CBS 130835, Ft 26	*Solanum lycopersicum*	USA	KR003337	KR003342	KR003347	KR003352	KR003357	KR003362	This study
C. lilii	CBS 109214, BBA 62147	*Lilium* sp.	Japan	GU227810	GU228104	GU227908	GU228006	GU228300	GU228202	Damm et al. (2009)
C. lilii	CBS 186.30	*Lilium* sp.	Netherlands	GU227811	GU228105	GU227909	GU228007	GU228301	GU228203	Damm et al. (2009)
C. liriopes	CBS 119444*	*Lirope muscari*	Mexico	GU227804	GU228098	GU227902	GU228000	GU228294	GU228196	Damm et al. (2009)
C. liriopes	CBS 122747	*Lirope muscari*	Mexico	GU227805	GU228099	GU227903	GU228001	GU228295	GU228197	Damm et al. (2009)
C. spaethianum	CBS 167.49, BBA 4804*	*Hosta sieboldiana*	Germany	GU227807	GU228101	GU227905	GU228003	GU228297	GU228199	Damm et al. (2009)
C. spaethianum	CBS 100063	*Lilium* sp.	South Korea	GU227808	GU228102	GU227906	GU228004	GU228298	GU228200	Damm et al. (2009)
C. spaethianum	CBS 101631	*Hemerocallis sp.*	New Zealand	GU227809	GU228103	GU227907	GU228005	GU228299	GU228201	Damm et al. (2009)
C. tofieldiae	**CBS 127615**	*Agapanthus* sp.	Portugal	KR003334	KR003339	KR003344	KR003349	KR003354	KR003359	This study
C. tofieldiae	**Ct0861**	*Arabidopsis thaliana*	Spain	KR003333	KR003338	KR003343	KR003348	KR003353	KR003358	This study
C. tofieldiae	CGMCC 3.15118	*Bletilla ochracea*	China	JX625176	JX625205	KC843541	-	-	KC843504	Tao et al. (2013)
C. tofieldiae	IMI 288810	*Dianthus* sp.	UK	GU227803	GU228097	GU227901	GU227999	GU228293	GU228195	Damm et al. (2009)
C. tofieldiae	**CBS 168.49**	*Lupinus polyphyllus*	Germany	GU227802	GU228096	GU227900	GU227998	GU228292	GU228194	Damm et al. (2009)
C. tofieldiae	**CBS 130851**	*Semele gayae*	Germany	KR003335	KR003340	KR003345	KR003350	KR003355	KR003360	This study
C. tofieldiae	**CBS 495.85**	*Tofieldia calyculata*	Switzerland	GU227801	GU228095	GU227899	GU227997	GU228291	GU228193	Damm et al. (2009)
C. verruculosum	IMI 45525*	*Crotalaria juncea*	Zimbabwe	GU227806	GU228100	GU227904	GU228002	GU228296	GU228198	Damm et al. (2009)

(a) Whole-genome phylogeny, inferred from the number of substitutions per site detected across single copy genes that are conserved across six genome-sequenced *Colletotrichum* species (b) Phylogeny of eight *Colletotrichum* species in the *C. spaethianum* species complex obtained from a maximum parsimony analysis based on a multilocus alignment (ITS, TUB2, ACT, HIS3, CHS-1, GAPDH). The strains studied here are highlighted in bold. Bootstrap support values (500 replicates) above 70 % are shown at the nodes. *Colletotrichum dematium* was used as an outgroup. (c) List of *Colletotrichum* strains used for the phylogeny shown in (b), indicating the culture collection accession numbers, host plants, country of origin and Genbank accession numbers of the sequences included. #ATCC: American Type Culture Collection, Manassas, Virginia, USA; CBS: Culture Collection of the Centraalbureau voor Schimmelcultures, Fungal Biodiversity Centre, Utrecht, The Netherlands; CGMCC: China General Microbiological Culture Collection Center, Beijing, China; IMI: Culture Collection of CABI Europe UK Centre, Egham, UK; MAFF: MAFF Genebank Project, Ministry of Agriculture, Forestry and Fisheries, Tsukuba, Japan; *ex-holotype or ex-epitype cultures; accession numbers of strains studied here are bold; - no sequence available.

Supplementary Fig. 3: Whole genome alignment of five *Colletotrichum* species against *C. graminicola* chromosomes

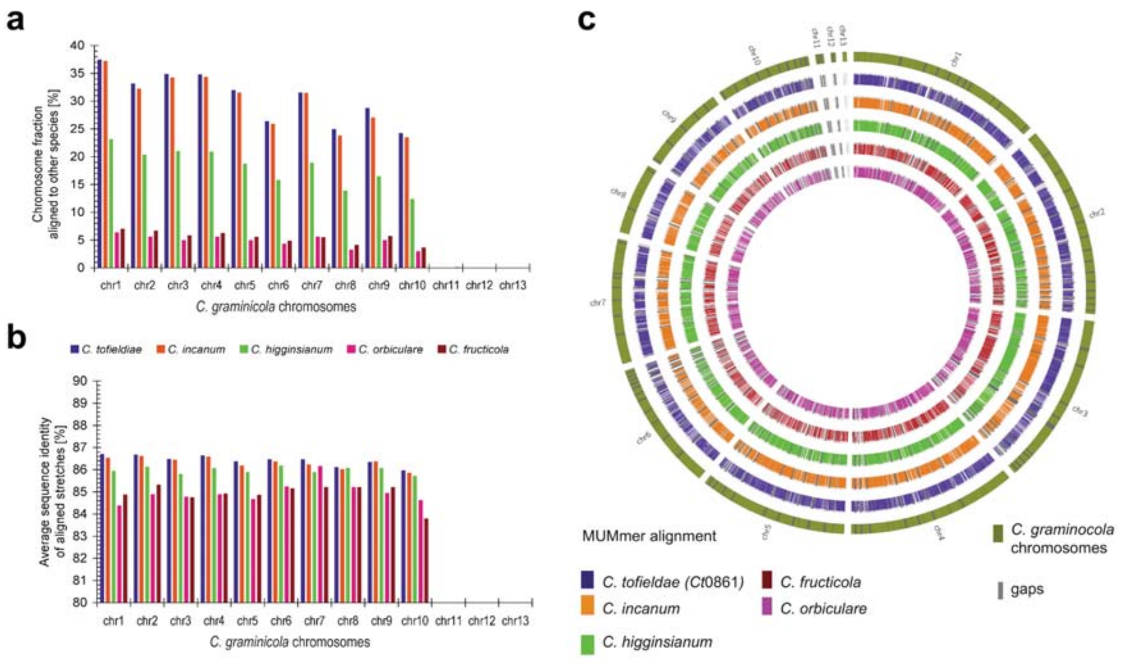

(a) Summary of the fraction of *C. graminicola* chromosomes that were mapped in pair-wise whole genome alignments to the assemblies of each the other five species. The pair-wise whole genome alignments were performed using MUMmer with default parameter settings. (b) Summary of the average sequence identity of the aligned stretches. (c) Circular plot showing that the aligned stretches are distributed more or less evenly along the *C. graminicola* chromosomes with exception of the three minichromosomes. The sequence identity was at least 74% in all alignments and the average length of the aligned stretches was ~500 bp for *C. orbiculare* and *C. fructicola*, ~800 bp for *C. higginsianum* and ~1200 bp for *C. tofieldiae* and *C. incanum*. The distribution of the aligned stretches along the *C. graminicola* chromosomes was visualized using the Circos software package.

Supplementary Fig. 4: Distribution of SNPs, sequence conservation and haplotype structure among five *C. tofieldiae* isolates

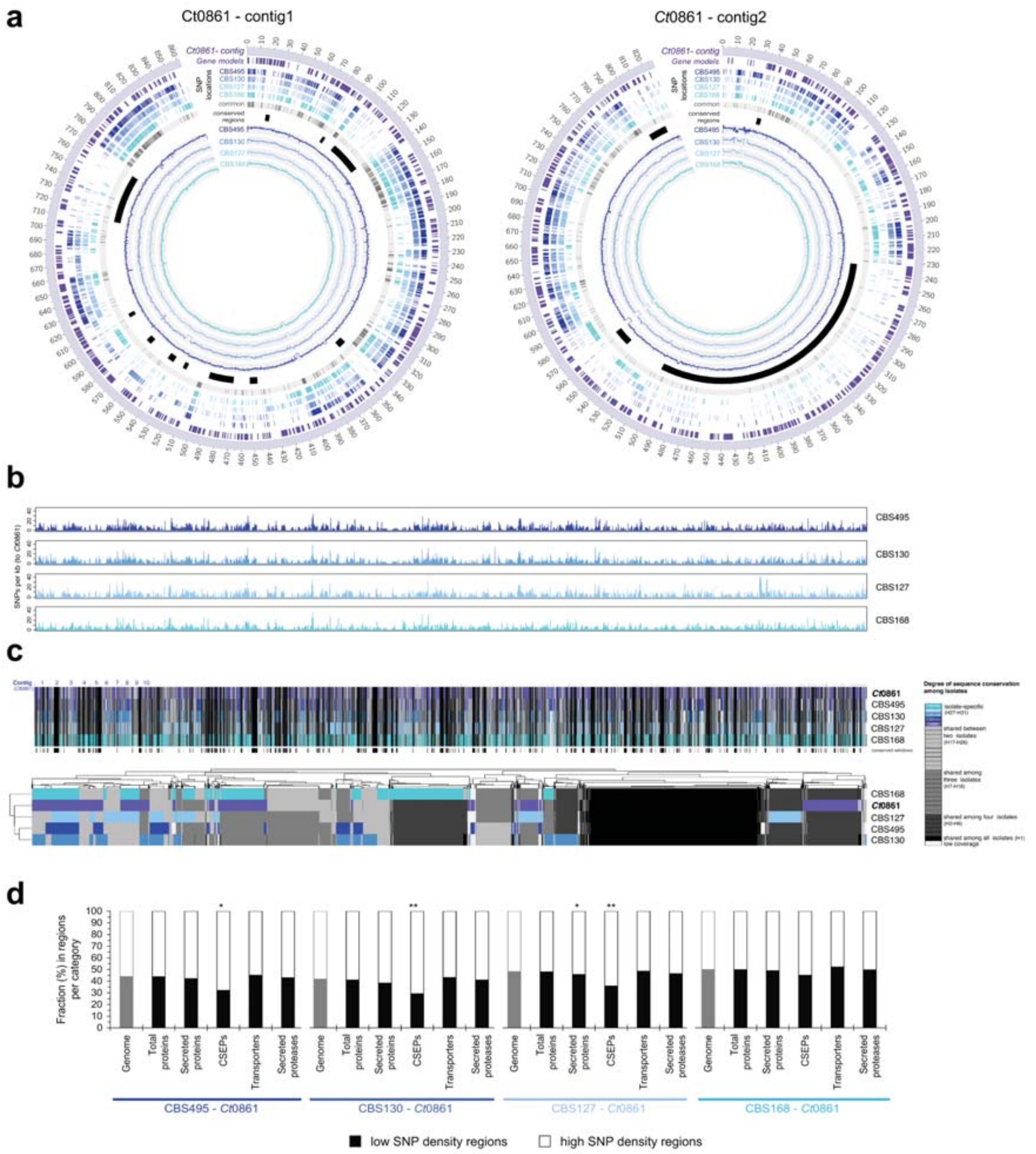

(a) Circular visualization of the alignment of *C. tofieldiae* isolates CBS495, CBS130, CBS127 and CBS168 genome sequencing reads and SNP locations with respect to the two largest contigs, contig 1 (left panel) and contig 2 (right panel), of the *C. tofieldiae* isolate 0861 reference genome. The tracks in the circle represent from the outside: the respective contig of the *Ct*0861 genome; locations of annotated *Ct*0861 gene models; locations of genomic SNPs to *Ct*0861 in CBS495, CBS130, CBS127, CBS168 (see Supplementary Table 1 for full culture IDs) and common SNPs between these four isolates; conserved regions with a low SNP density among all five isolates as identified through a hidden Markov model; genome sequencing read coverage (average per 100 bases) for isolates CBS495, CBS130, CBS127 and CBS168. The scale of the coverage plots ranges from 0 to 1000

(CBS495) and 0 to 500 (CBS130, 127, 168), respectively. (**b**) Distribution of SNP density (per kb) to *Ct*0861 in *C. tofieldiae* isolates CBS495, CBS130, CBS127 and CBS168 along all *Ct*0861 contigs larger than 50 kb. The SNP density was calculated in 10 kb sliding windows (moving by 1 kb) and windows were sorted for visualization in increasing order by contig number and position on the contig. Only windows with a sufficient sequencing coverage, calculated as average per nucleotide coverage in that window, in all isolates (≥ 100 reads for CBS130, CBS127, CBS168 and ≥ 200 reads for CBS495) were included in the analysis. (**c**) Heatmap visualization of sequence conservation and haplotype structure among the five *C. tofieldiae* isolates. To assess sequence conservation between isolates, all *Ct*0861 contigs larger than 50 kb were split in sliding windows of 10 kb (moving by 1 kb). The degree of conservation in each window was judged based on the SNP density in this window to each of the other isolates. The sequence in a window was assumed to be shared with another isolate (i.e. the isolates share the same haplotype), if the SNP density to the other isolate was classified as "low" in this window (using a two-component mixture model in combination with a hidden Markov model for prediction of low vs. high SNP density). If the SNP density to all of the other isolates was classified as high, the sequence was assumed to be isolate-specific. Depending on with which other isolates the sequence is shared, each window could be assigned one of 31 possible haplotypes (H1-H31) for each isolate (see also Supplementary Table 6) If the average read coverage per nucleotide in this window was below 50 (CBS130, CBS127, CBS168) or 100 (CBS495) the window was termed low coverage (LC) in the respective isolate. The color code in the heatmaps visualizes the degree of sequence conservation among isolates, by detailing for each isolate with how many other isolates the sequence is shared in each window. For the heatmap in the upper panel, the sequence windows were sorted in increasing order by contig number and position to show the arrangement of haplotype blocks within the *Ct*0861 genome. For the heatmap in the lower panel, the data was clustered by isolates and sequence windows (using complete linkage hierarchical clustering with the Euclidean distance as distance measure), to group together windows with similar haplotypes across isolates and isolates with a similar distribution of haplotype blocks. (**d**) Gene distribution between high and low SNP density regions between *Ct*0861 and each of the other four isolates. The regions were obtained from the 10 kb sliding windows by merging adjacent windows of either high or low SNP density. For each pairwise comparison the fraction of the *Ct*0861 genome with high or low SNP density was calculated. Subsequently, we extracted all *Ct*0861 genes that could be assigned unequivocally to either a high or low SNP density region (with >50% of the gene length located in the region) and calculated for each pairwise comparison the fraction of genes in high and low SNP density regions. In the same way, we also analyzed the distribution of four specific functional gene categories encoding secreted proteins, candidate secreted effector proteins (CSEPs), transporters and secreted proteases. To test for any significant association with either high or low SNP density regions, we compared the observed gene proportions with the underlying genome proportions using a Chi-square goodness-of-fit test. Cases where the gene distribution deviates significantly from the underlying genome distribution are marked with asterisks in the plot (* $p<0.05$; ** $p<0.01$).

Supplementary Fig. 5: Reconstruction of ancestral genomes, prediction of gene loss/gain and associated functional enrichment analysis.

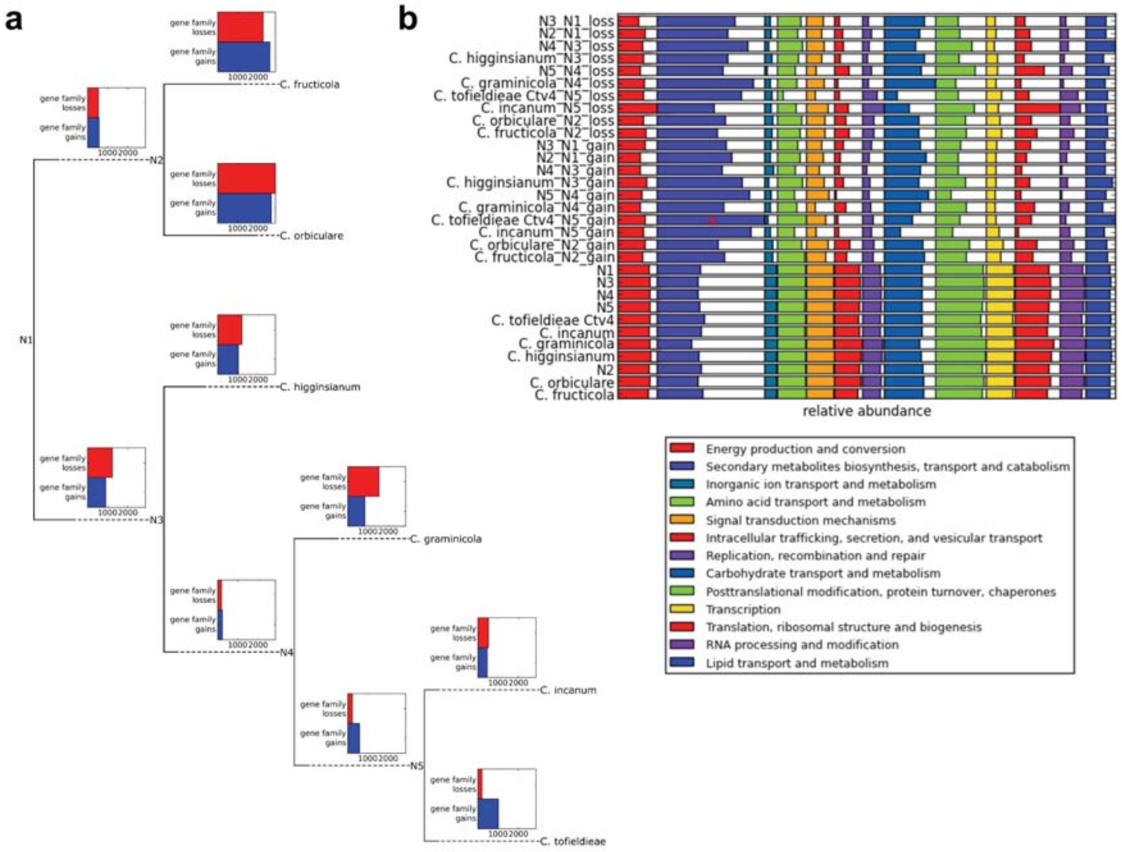

(a) Evolutionary dynamics of gene gains and losses (GGL) on the branches of the *Colletotrichum* species tree. GLOOME[2] reports the posterior probability for a gene family to be gained or lost for each branch of the tree (exceeding an insignificance threshold of 0.05). The number of genes families gained or lost for each branch was approximated by summing up the individual posterior probabilities for each gene family to be gained or lost on that branch and rounding this number to the closest integer. Dashed lines extend the tree branches to provide the extra space the GGL bar plots need. (b) Relative abundances for the 13 most abundant COG (Clusters of Orthologous Groups) functional categories within the genomes of the ancestral and extant *Colletotrichum* species, and the fraction of genes families from these categories that were gained or lost, for the individual branches of the species tree. Gene families were linked to the COG functional categories via the fuNOG annotation. The significantly increased number of gene families from the 'Secondary metabolites biosynthesis, transport and catabolism' category on the branch leading to *C. tofieldiae* is indicated by a red star. The COG categories 'Function Unknown' and 'General Functional Prediction only' were omitted from the plot.

Supplementary Fig. 6: d_n/d_S ratios of protein families from the ten *Colletotrichum* genomes grouped by fuNOG annotation on the second level (lvl2) and ordered by descending median d_n/d_S ratio.

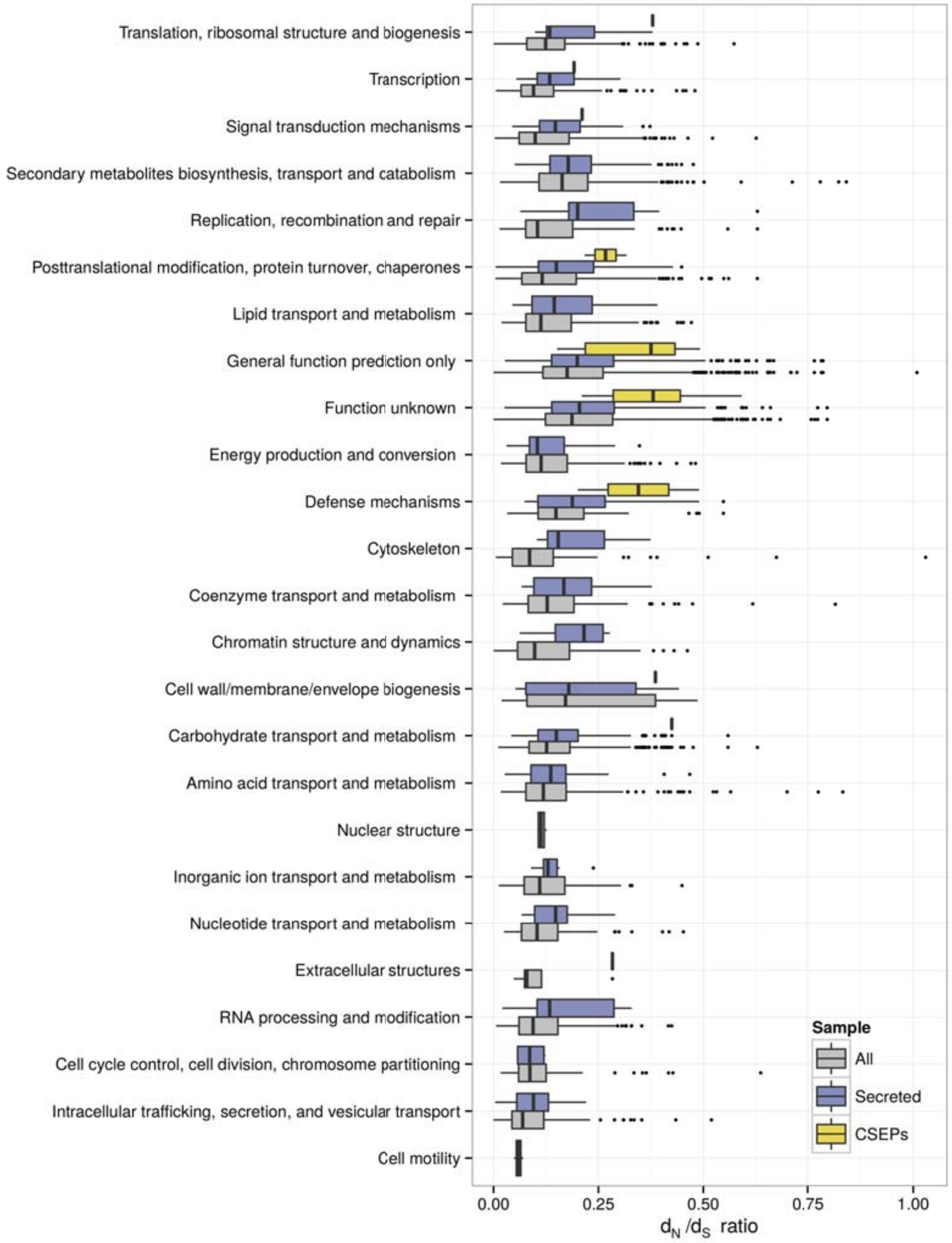

Ratio of synonymous mutations per synonymous sites versus non-synonymous mutations per non-synonymous sites (d_n/d_S) was evaluated for 13,458 protein families (OrthoMCL) identified in the genomes of ten *Colletotrichum* fungi, including five *C. tofieldiae* isolates, *C. incanum*, *C. graminicola*, *C. higginsianum*, *C. orbiculare* and *C. fructicola*. These protein families were grouped into 25 fuNOG categories. A high ratio indicates evidence for positive selection. Secreted: secreted protein. CSEP: candidate secreted effector protein. All: all genes categories.

Supplementary Fig. 7: Second-level fuNOG functional categories (lvl2) showing significant evidence of positive selection in the genomes of pathogenic *Colletotrichum* vs. *C. tofieldiae* isolates (a) and *vice versa* (b).

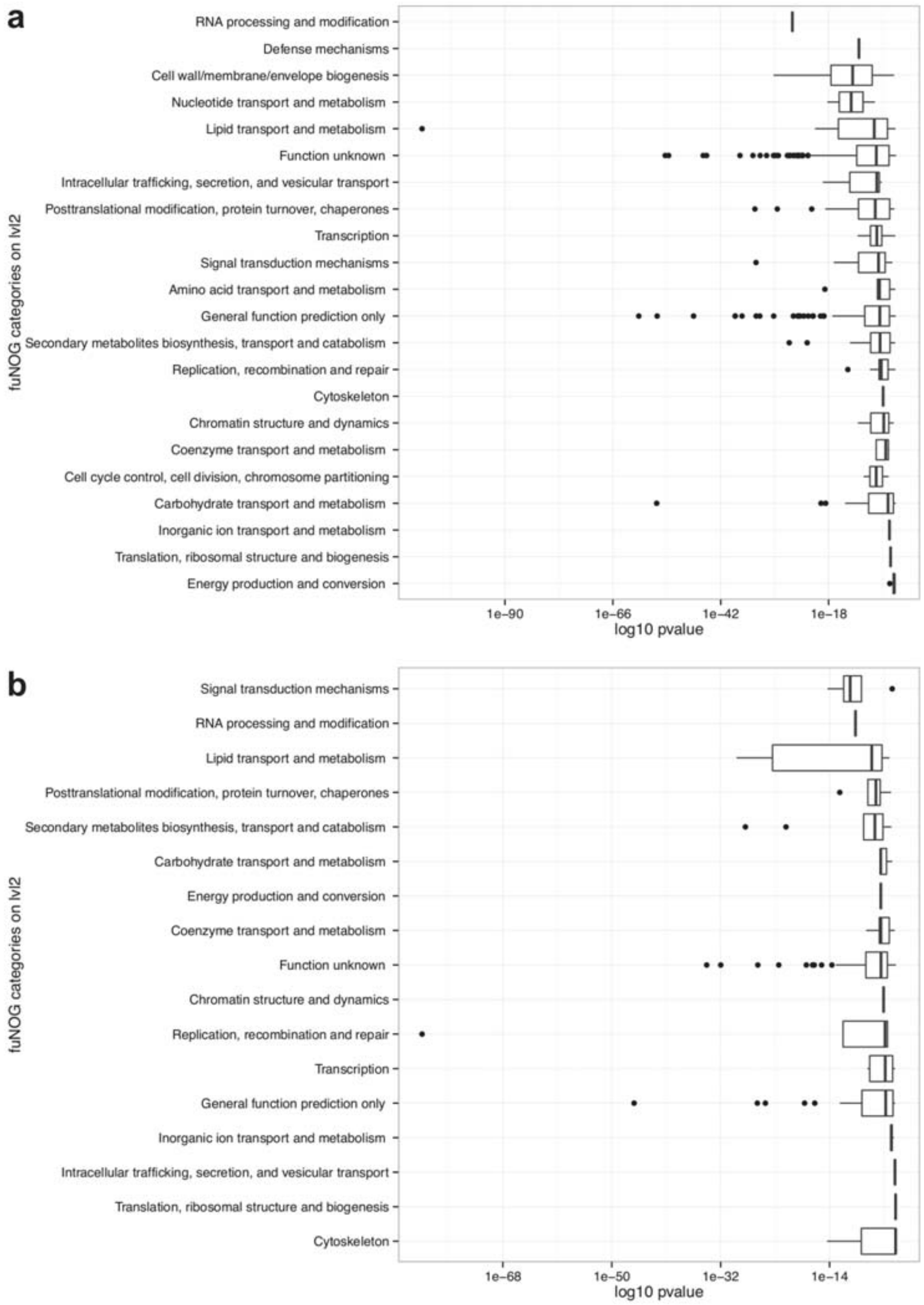

(a) Functional categories showing significant evidence of positive selection in the genomes of five pathogenic *Colletotrichum* species (*C. incanum*, *C. graminicola*, *C. higginsianum*, *C. orbiculare* and *C. fructicola*) vs. the five *C. tofieldiae* isolates. (b) Functional categories showing significant evidence of positive selection in the genomes of the five *C. tofieldiae* isolates vs. pathogenic *Colletotrichum* species. A significant enrichment (*p*-value<0.001) of large d_N/d_S values per gene family was tested using the one-sided Fisher's exact and the FDR for multiple testing correction (alpha=0.5).

Supplementary Fig. 8: Fraction of *C. tofieldiae* proteins conserved in the genomes of mycorrhizal fungi and plant-associated fungal endophytes

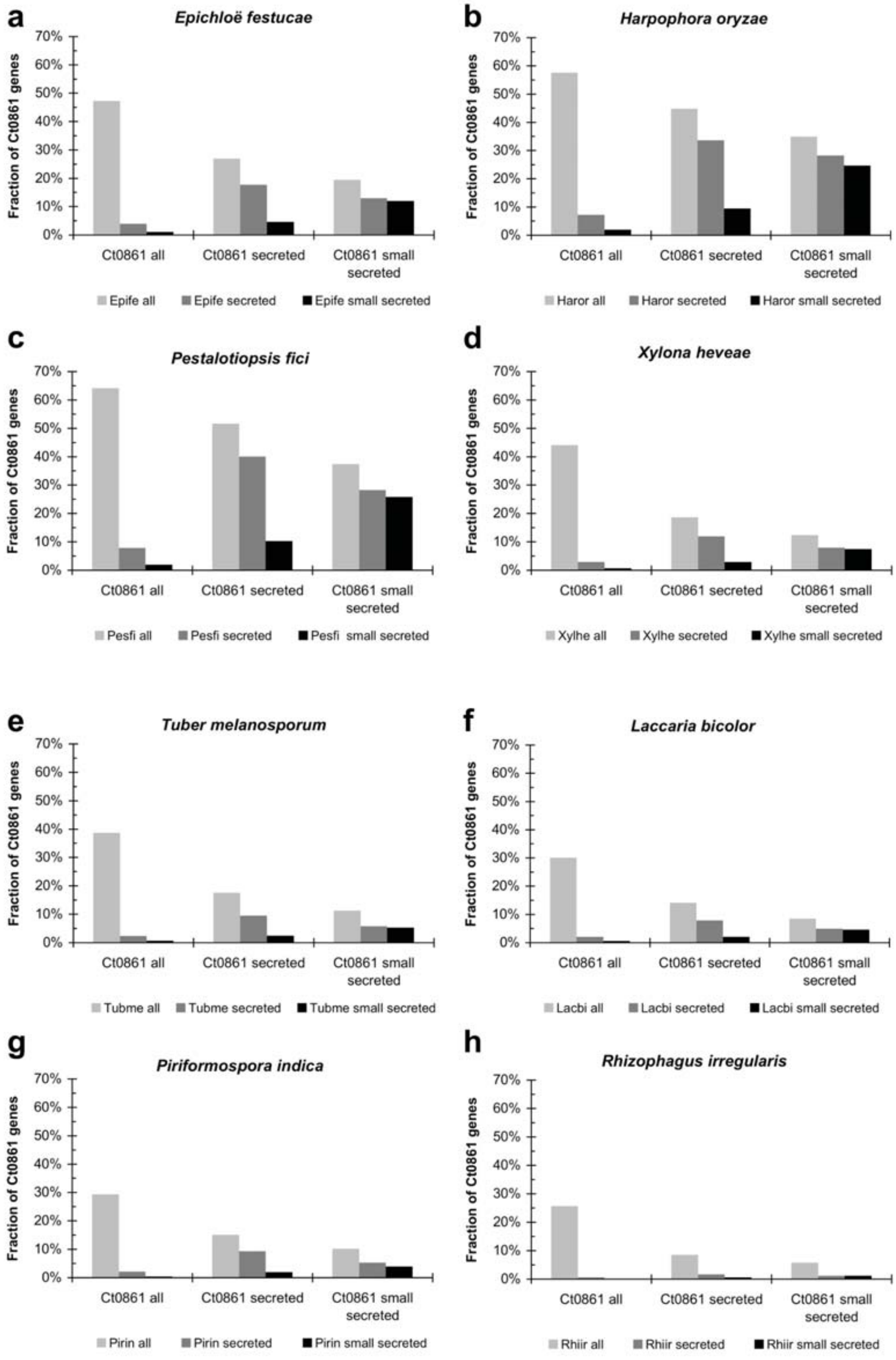

Summary of the fraction of *C. tofieldiae* isolate 0861 proteins with homologs in the proteomes of five endophytic/mutualistic Ascomycota species (a) *Epichloë festucae* (Sordariomycetes), (b) *Harpophora oryzae* (Sordariomycetes), (c) *Pestalotiopsis fici* (Sordariomycetes), (d) *Xylona heveae*

(Xylonomycetes) and (e) *Tuber melanosporum* (Pezizomycetes), as well as three further species of mutualistic root-associated fungi (f) *Laccaria bicolor* (Basidiomycota), (g) *Piriformospora indica* (Basidiomycota) and (h) *Rhizophagus irregularis* (Glomeromycota). Homologous proteins between species were identified as bi-directional best BLAST hits with an e-value threshold of 1e-6. The fraction of conserved proteins is evaluated for the whole proteome ('all') and additionally for the subsets of 'secreted' and 'small secreted' proteins. For each of these protein sets in *Ct*0861, the fraction of proteins with homologs within each set in the other endophytes is calculated. Secreted proteins were predicted using WoLF-PSORT (http://www.genscript.com/psort/wolf_psort.html) and secreted proteins with a length <300 amino acids were classified as 'small secreted' proteins.

Supplementary Fig. 9: Comparative analysis of genes encoding secreted proteins and secreted proteases in the genomes of *Colletotrichum* species, mycorrhizal fungi and plant-associated fungal endophytes

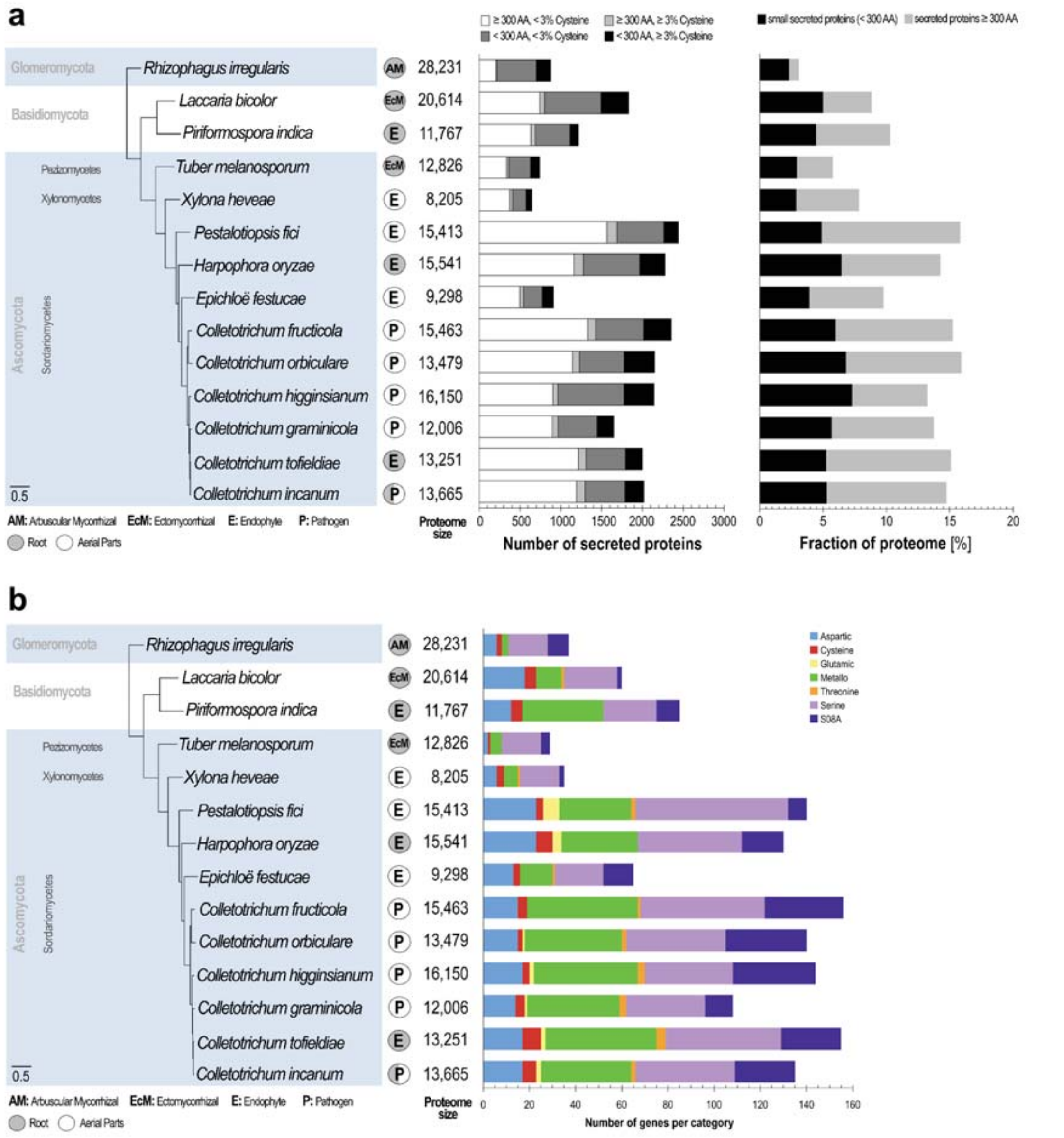

(a) Predicted secretome repertoires in six *Colletotrichum* species and eight endophytic/mutualistic fungi. The left barplot shows the number of secreted proteins as predicted by WoLF-PSORT (www.genscript.com/psort/wolf_psort.html), subdivided by protein length (<300 AA ⇒ 'small') and cysteine content (≥3% ⇒ 'high'). The right barplot shows the corresponding fractions of secreted proteins in the proteome of each species, again subdivided by protein length. (b) Classification of genes encoding putative secreted proteases. The protease homologs among the predicted secreted proteins were classified according to the MEROPS database (http://merops.sanger.ac.uk). S08A corresponds to the subtilisin group of serine proteases. Left of the barplots, a phylogeny of the analyzed species is included (see Supplementary Methods), together with information on fungal lifestyle, host niche and total proteome size.

Supplementary Fig. 10: Comparative analysis of genes encoding transporters in the genomes of *Colletotrichum* species, mycorrhizal fungi and plant-associated fungal endophytes

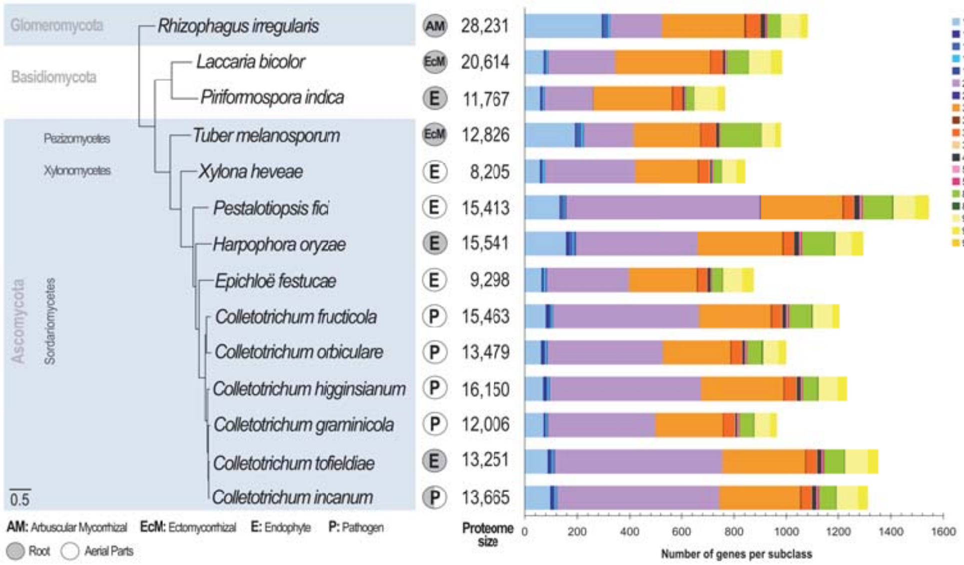

Classification of genes encoding putative transporters in *Colletotrichum* fungi compared to eight different endophytic or mutualistic fungi. Transporters were annotated and classified according to the Transporter Classification Database (TCDB) (http://www.tcdb.org/). The number of putative transporters and their classification into TCDB subclasses is highly similar between *C. tofieldiae* and *C. incanum*. Left of the barplots, a phylogeny of the analyzed species is included (see Supplementary Methods), together with information on fungal lifestyle, host niche and total proteome size.

Supplementary Fig. 11: Comparative analysis of secondary metabolism key genes and clusters in the genomes of *Colletotrichum* species, mycorrhizal fungi and plant-associated fungal endophytes.

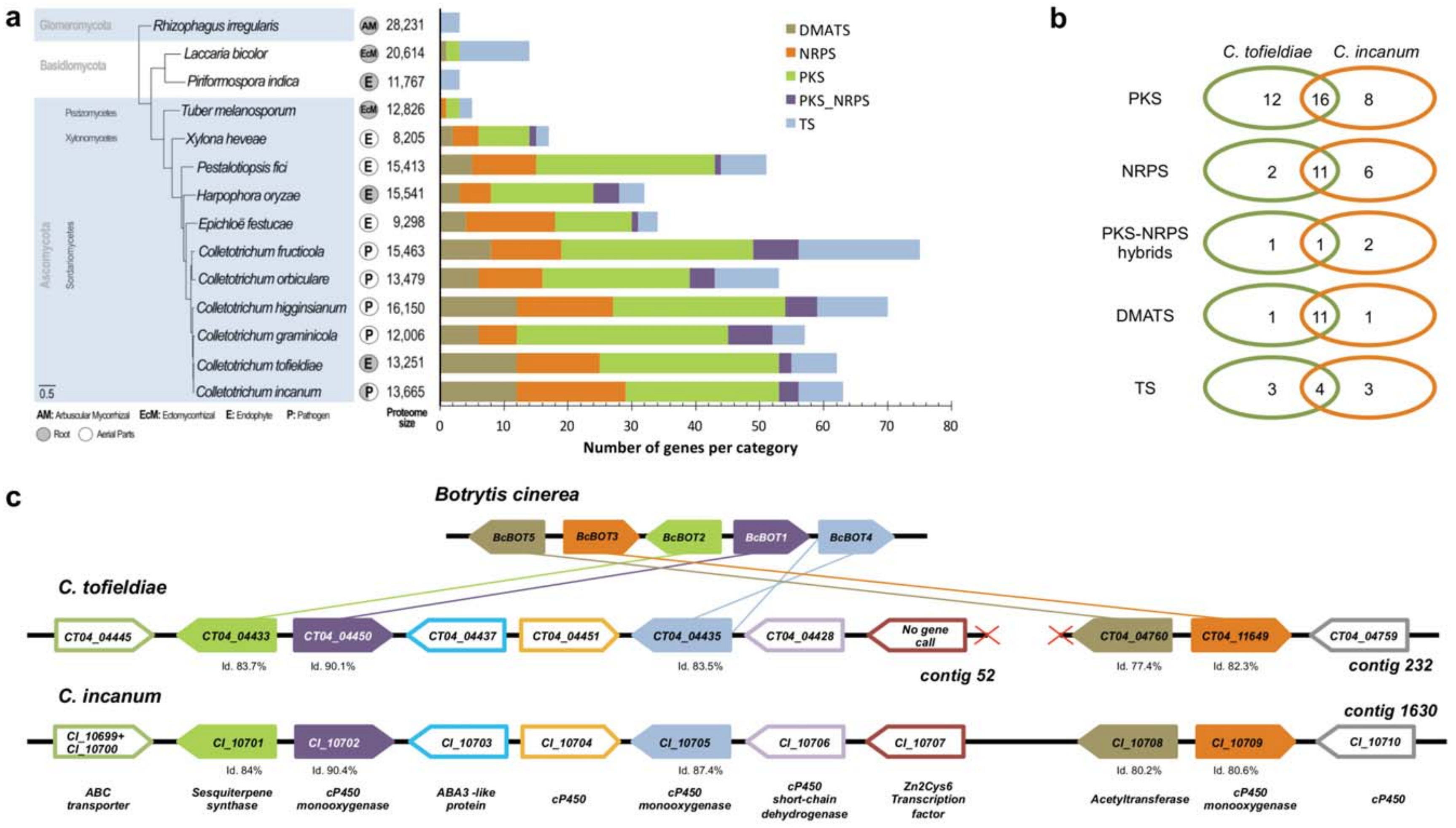

(**a**) Secondary metabolism key genes (SMKGs) predicted from fourteen fungal genomes using an in-house bioinformatics pipeline. (**b**) Venn diagram of SMKGs shared between *C. tofieldiae* and *C. incanum* for each key gene subclass. Left of the barplot, a phylogeny of the analyzed species is included (see Supplementary Methods), together with information on fungal lifestyle, host niche and total proteome size. (**c**) Occurrence of the botrydial gene cluster in *C. tofieldiae* and *C. incanum,* which was previously described only in *Botrytis cinerea.* Solid arrows correspond to genes originally described by Pinedo *et al.*[3], with percentage amino acid identity to the *Botrytis* proteins shown below. Empty arrows are secondary metabolism-related genes putatively part of the botrydial gene cluster. Synteny is indicated with solid lines. Note the cluster is split between two contigs in the *C. tofieldiae* assembly. DMATS: dimethylallyl tryptophan synthases. PKS: polyketide synthases. NRPS: nonribosomal peptide synthetases. PKS-NRPS: hybrid PKS-NRPS. TS: terpene synthases. The *C. tofieldiae* “No gene call” coordinates are contig_52:1971-3335.

Supplementary Fig. 12: Comparative analysis of CAZyme modules predicted in the genomes of *Colletotrichum* species, mycorrhizal fungi and plant-associated fungal endophytes

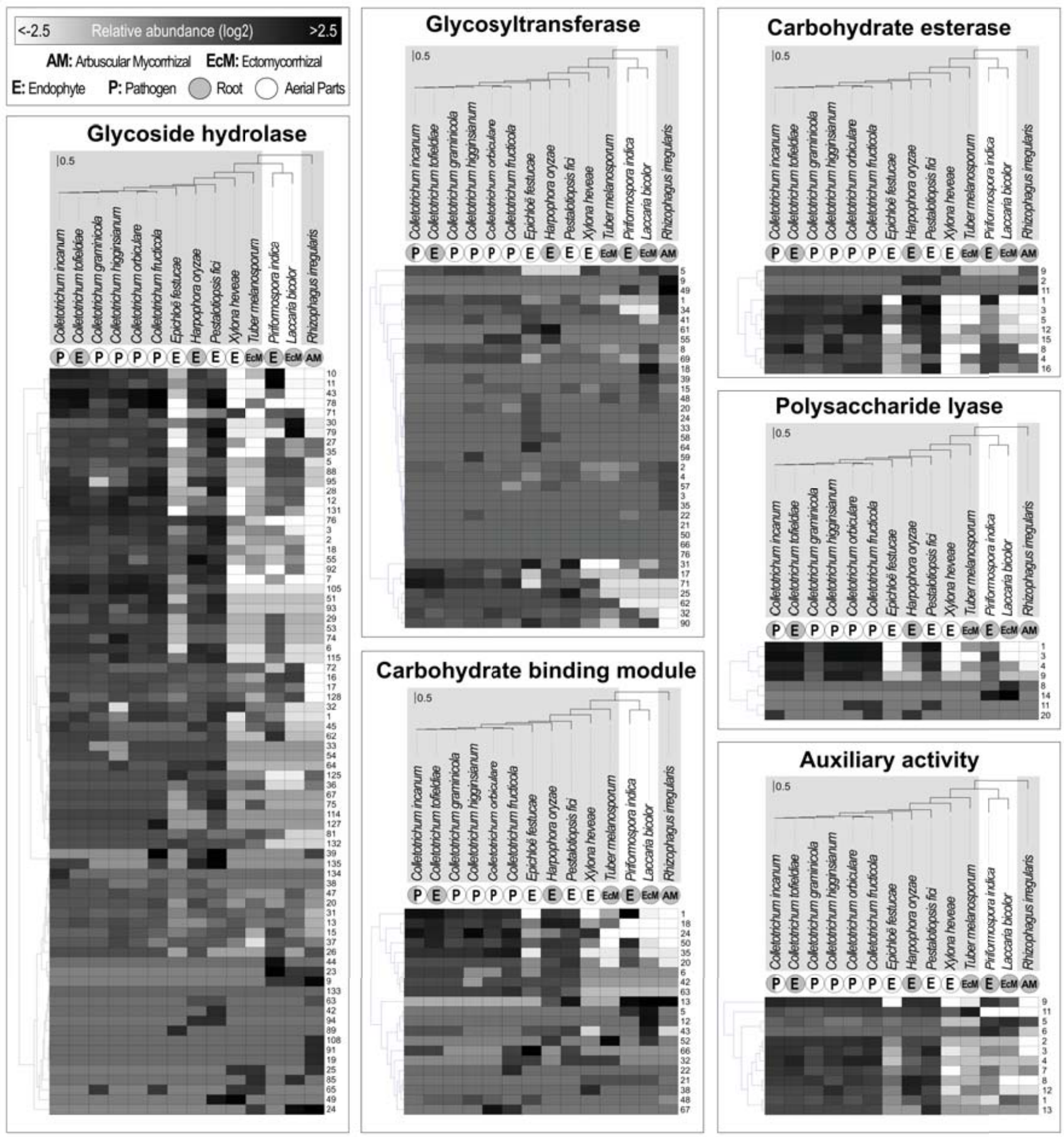

Hierarchical clustering of CAZyme families detected in the genomes of n six *Colletotrichum* species and eight endophytic/mutualistic fungi. The Relative Abundance Index (RAI) is depicted as log2-fold changes relative to the familly mean. For each CAZyme family, overrepresented modules are depicted in black whereas underrepresented are indicated in white. The numbers on the right side of the heatmaps indicates the corresponding CAZyme families. Above the heatmaps, a phylogeny of the analyzed species is included (see Supplementary Methods), together with information on fungal lifestyle and host niche.

Supplementary Fig. 13: Comparative analysis of CSEP repertoires among *C. tofieldiae* isolates

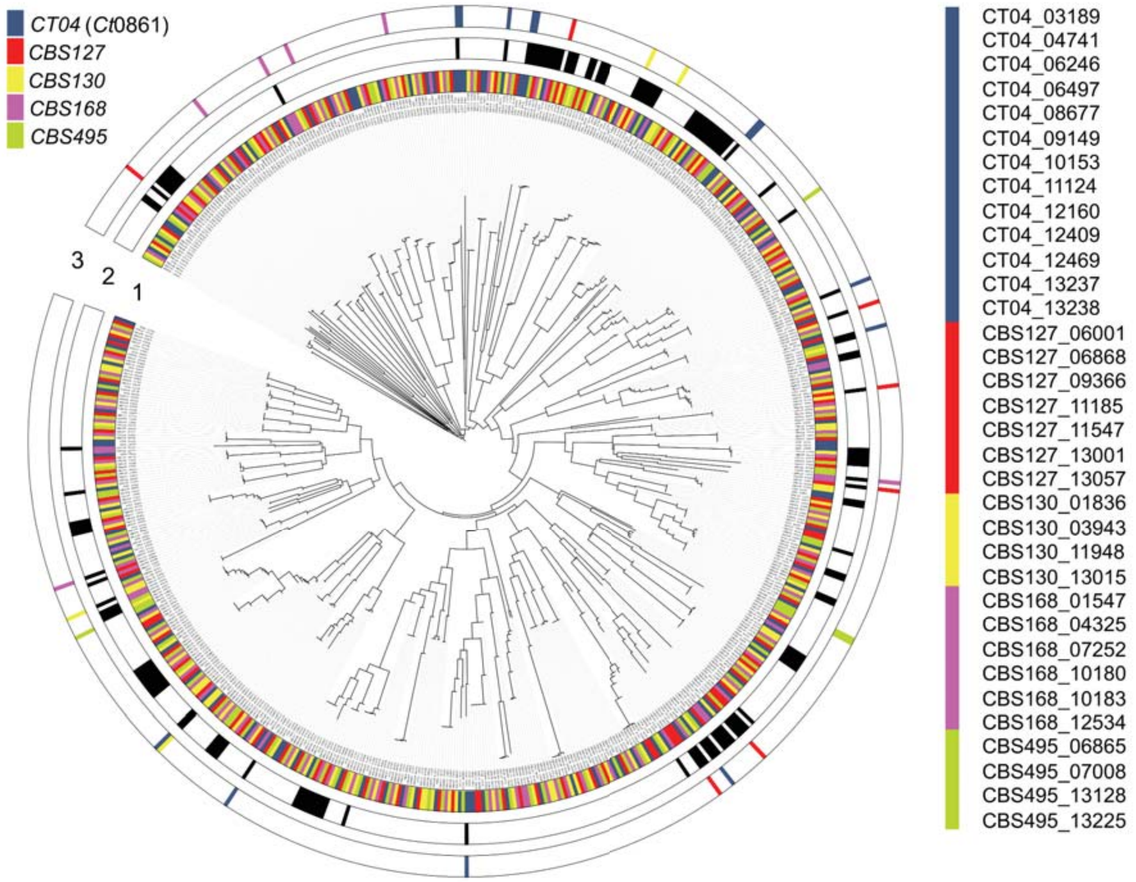

The Neighbor-joining tree was constructed in MEGA5[4] using the protein sequences of all candidate secreted effector proteins (CSEPs, 648 in total) identified in the genomes of *C. tofieldiae* isolates *Ct*0861, CBS127, CBS130, CBS168 and CBS495. The tree was edited using iTOL (interactive Tree Of Life, http://itol.embl.de/) in order to visualize the CSEPs repertoires of each isolates. 1: Color codes indicate which CSEP corresponds to which *C. tofiediae* isolate. 2: black lines indicate species-specific effectors. 3: isolate-specific effectors are highlighted in color, similar to the color code depicted in 1. For each isolate, gene identification numbers of isolate-specific CSEPs are indicated on the right part of the figure.

Supplementary Fig. 14: Expression profiling of *C. tofieldiae* genes encoding CBM domain-containing proteins

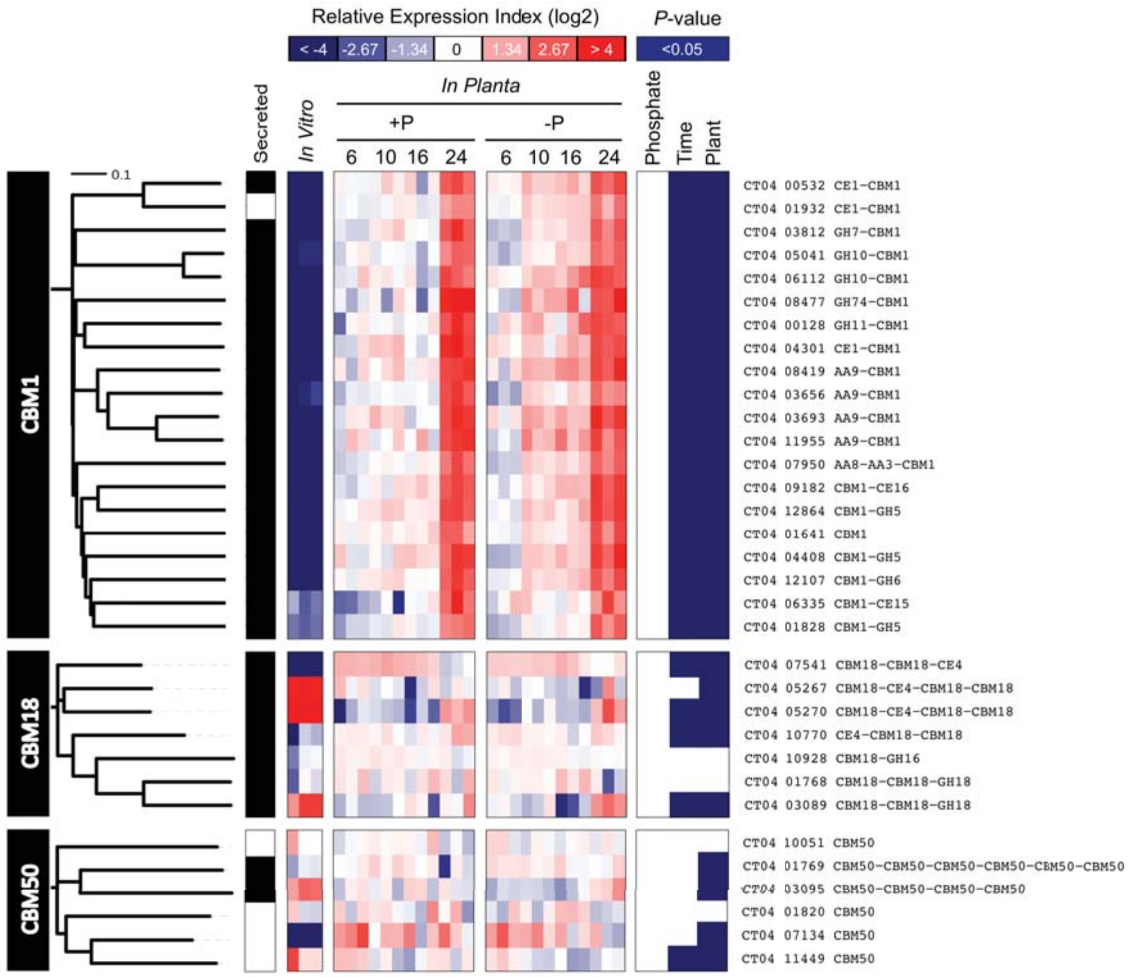

Heatmaps of gene expression are depicted for the genes encoding carbohydrate-binding module containing proteins (CBM1, CBM18 and CBM50). Expressed genes are represented, together with their phylogenetic relationships. Overrepresented (pale red to dark red) and underrepresented transcripts (pale blue to dark blue) are shown as log2 fold changes relative to the mean expression across all nine stages. Secreted proteins are highlighted in black on the left side of the heatmaps. Significantly regulated genes (|log2FC|≥1, FDR<0.05) between phosphate conditions, time points or *in vitro* vs. *in planta* are depicted in blue on the right side of the heatmap. Interactive Tree Of Life (iTOL, http://itol.embl.de/) has been used to assemble phylogenetic relationships with gene expression and *p*-value data. CBM1: cellulose binding domain. CBM18: chitin-binding domain. CBM50: LysM chitin-binding domain. Note that many CBM1 modules are appended to cellulases.

Supplementary Fig. 15: Experimental setup for dual RNAseq analysis of *Arabidopsis* and fungal transcriptomes

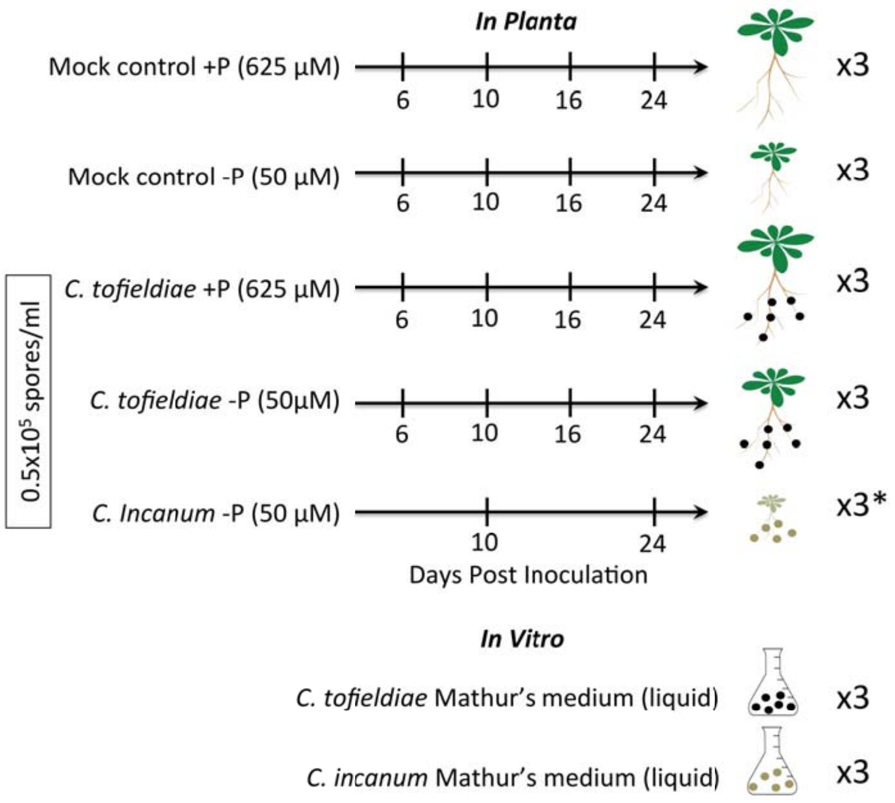

A time-course experiment was conducted using four *in planta* time points for the C*. tofieldiae*-*Arabidopsis* interaction (6, 10, 16 and 24 dpi) and two *in planta* time points for the *C. incanum*-*Arabidopsis* interaction (10, 24 dpi). For the C*. tofieldiae-Arabidopsis* interaction, both phosphate sufficient (625µM) and deficient (50µM) conditions were used in order to disentangle the impact of phosphate nutritional status on the outcome of the interaction (i.e. neutral at high [Pi] and beneficial at low [Pi] compared with mock treated plants). In contrast with *C. tofieldiae*, C*. incanum* has a clear deleterious impact on plant growth under phosphate-deficient conditions. Therefore we only focused on the phosphate-deficient condition (50µM) for the *C. incanum-Arabidopsis* interaction. Control plants (mock-treated) were also harvested at each time point to compare the gene expression patterns in colonized vs. mock-treated plants. For each fungal species, the *in vitro* fungal transcriptome corresponding to the fungal mycelium grown in a liquid culture has been included as well. For each sample, three biological experiments were performed. *This experiment includes three biological replicates plus three additional biological replicates for which the fungal read coverage was very low (see Supplementary Table 8).

Supplementary Fig. 16: Multi-dimensional scaling (MDS) plots of distances between gene expression profiles for each of the analyzed expression datasets

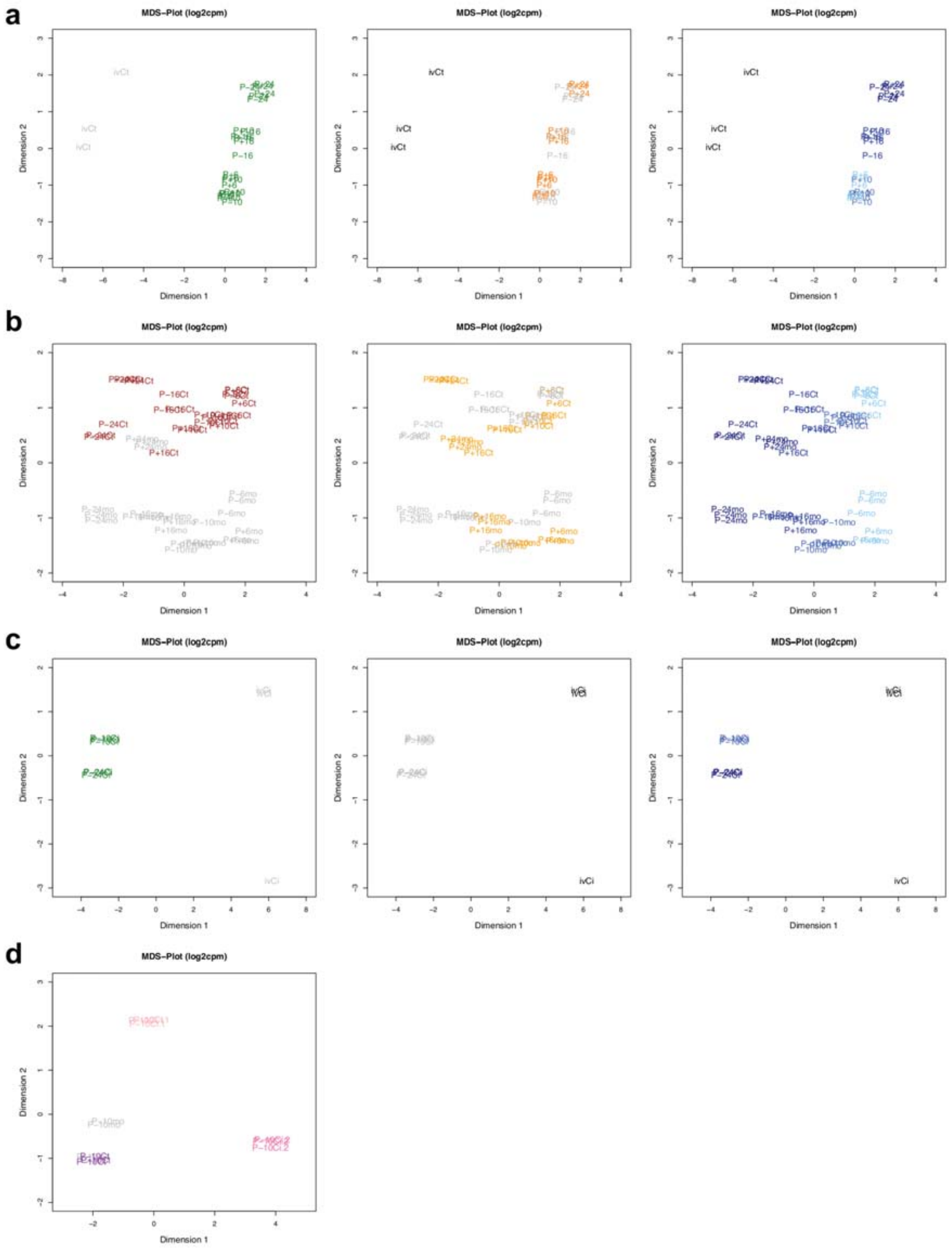

For all datasets, the Euclidean distance (root-mean-square deviation) is used as distance measure between pairs of samples, such that the distances in the plot can be interpreted as approximations of the typical log2 fold changes between the samples. For the datasets shown in (a)-(c), the same MDS

plot is shown three times with different color-coding to highlight different aspects of the dataset. (a) MDS plots for the *C. tofieldiae* expression data during *A. thaliana* infection. In the left panel, samples are colored by fungal culture condition: grey = *in vitro*, green = *in planta*. In the middle panel, samples are colored by nutrient status: grey = phosphate-deficient (*in planta*), orange = phosphate-sufficient (*in planta*), black = phosphate-sufficient (*in vitro*). In the right panel the samples are colored by time-point: light blue → dark blue = 6 dpi → 24 dpi, black = *in vitro*. (b) MDS plots for the *A. thaliana* expression data in response to infection with *C. tofieldiae*. In the left panel, samples are colored by infection status: grey = mock, brown = *Ct* infected. In the middle panel, samples are colored by nutrient status: grey = phosphate-deficient, orange = phosphate-sufficient. In the right panel the samples are colored by time-point: light blue → dark blue = 6 dpi → 24 dpi. (c) MDS plots for the *C. incanum* expression data during *A. thaliana* infection. In the left panel, samples are colored by fungal culture condition: grey = *in vitro*, green = *in planta*. In the middle panel, samples are colored by nutrient status: grey = phosphate-deficient (*in planta*), black = phosphate-sufficient (*in vitro*). In the right panel the samples are colored by time-point: light blue → dark blue = 6 dpi → 24 dpi, black = *in vitro*. (d) MDS plot for the *A. thaliana* expression data in response to infection with either *C. tofieldiae* or *C. incanum* at 10 dpi in phosphate-deficient conditions. Samples are colored by infection status: grey = mock, purple = *Ct* infected, pink = *Ci* infected (light pink: first experiment, dark pink: second experiment).

Supplementary Fig. 17: Normalization of *C. tofieldiae* gene expression data

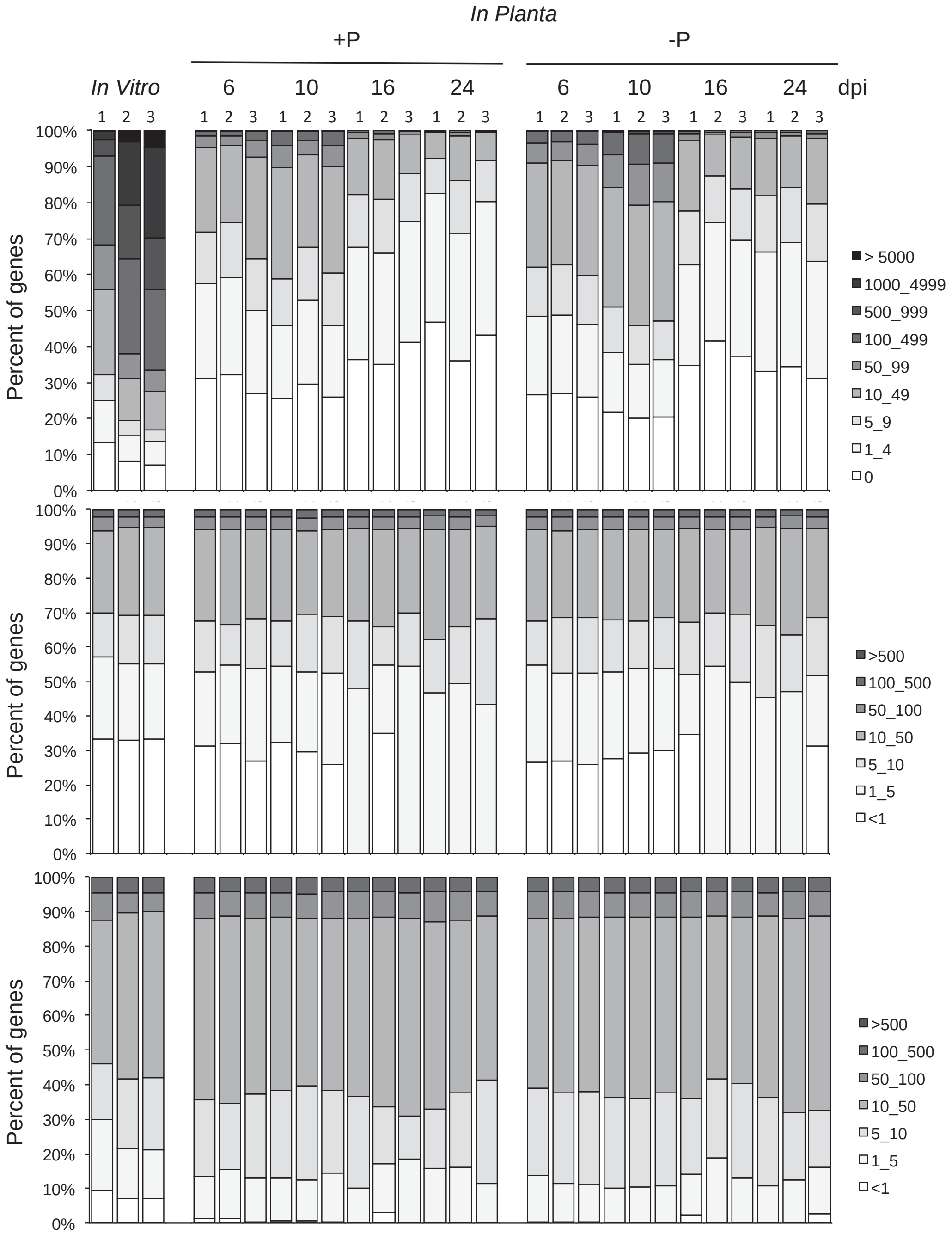

The upper panel corresponds to the percentage of genes having high (black) to low (white) read count coverage before normalization (i.e. raw read counts). As expected, a much lower read coverage was detected during *in planta* colonization of *Arabidopsis* roots by *C. tofieldiae* compared with the fungus grown in liquid culture. Similarly, the middle panel shows the percentage of genes with high and low read coverage but after library size normalization. The high number of genes with undetectable or low read counts introduces a bias during normalization, especially for the genes with <5 reads. By applying a stringent cuttoff (i.e. removal of the genes with <100 reads across all 24 *in planta* conditions, lower pannel), we significantly reduced this bias and therefore decided to focus our analysis on these 6,693 *in planta*-expressed genes.

Supplementary Figure 18: Validation of RNAseq data by RT-qPCR.

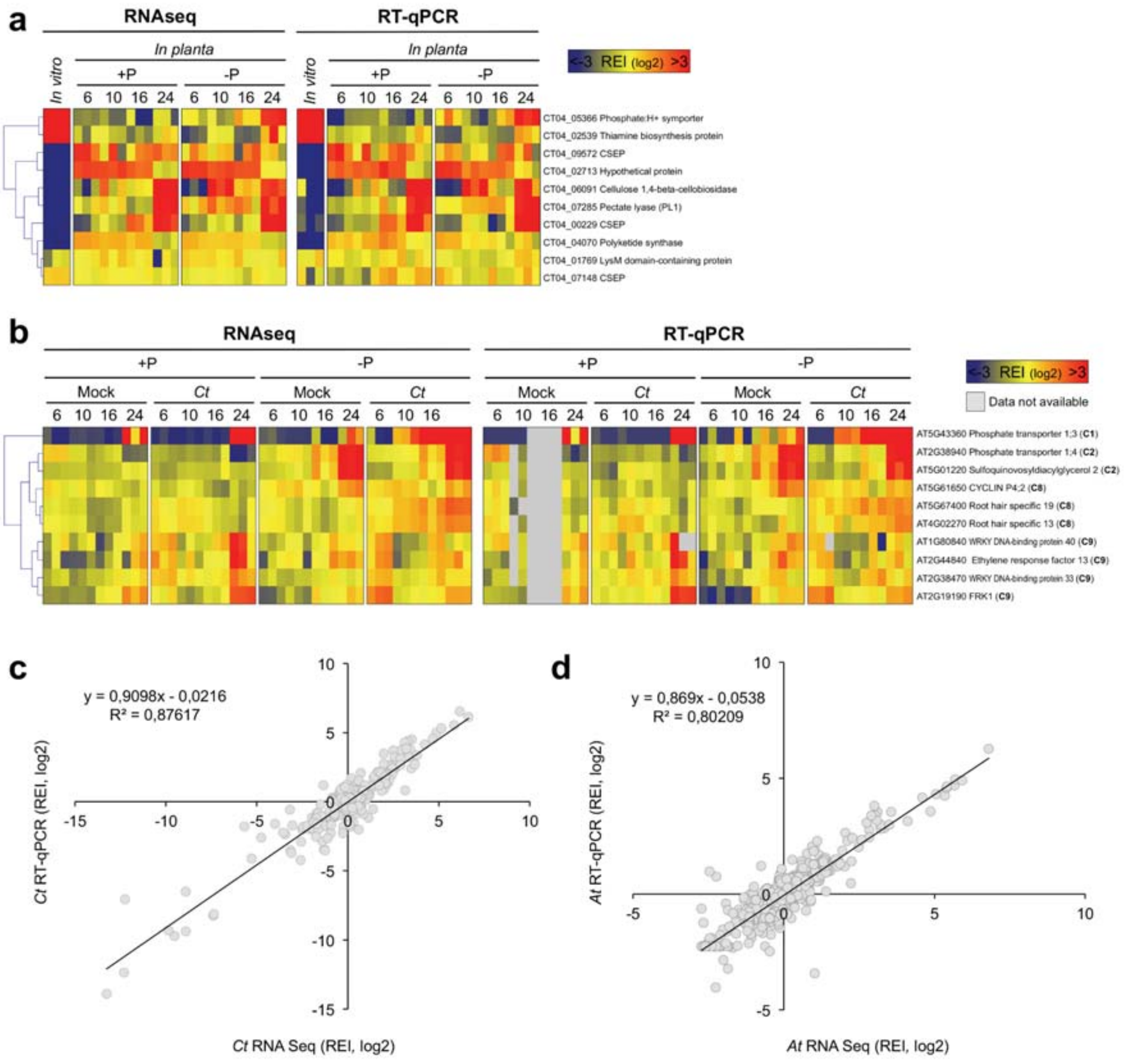

(**a**) Expression profiles of 10 *C. tofieldiae* genes during *Arabidopsis* root colonization using RNAseq (left) and RT-qPCR (right). RT-qPCR gene expression levels were normalized to the transcript levels of the *C. tofieldiae* reference gene tubulin beta 1 chain (CT04_12898). Overrepresented (light red to dark red) and underrepresented transcripts (light blue to dark blue) are shown as log2-fold changes relative to the mean expression measured across all samples (REI: Relative Expression Index). (**b**) Expression profiles of 10 *A. thaliana* genes in mock-treated and *Ct*-colonized roots using RNAseq (left) and RT-qPCR (right). RT-qPCR gene expression levels were normalized to the transcript levels of the *Arabidopsis* reference gene Actin 2 (AT3G18780). Overrepresented (light red to dark red) and underrepresented transcripts (light blue to dark blue) are shown as log2-fold changes relative to the mean expression measured across all samples. (**c**) Correlation between *C. tofieldiae* gene expression levels measured by RT-qPCR and RNAseq. (**d**) Correlation between *A. thaliana* gene expression levels measured by RT-qPCR and RNAseq. All selected genes as well as the primer pairs used in this study are presented in the Supplementary Table 9.

Supplementary Fig. 19: Number of differentially expressed *Arabidopsis* and *C. tofieldiae* genes identified per comparison

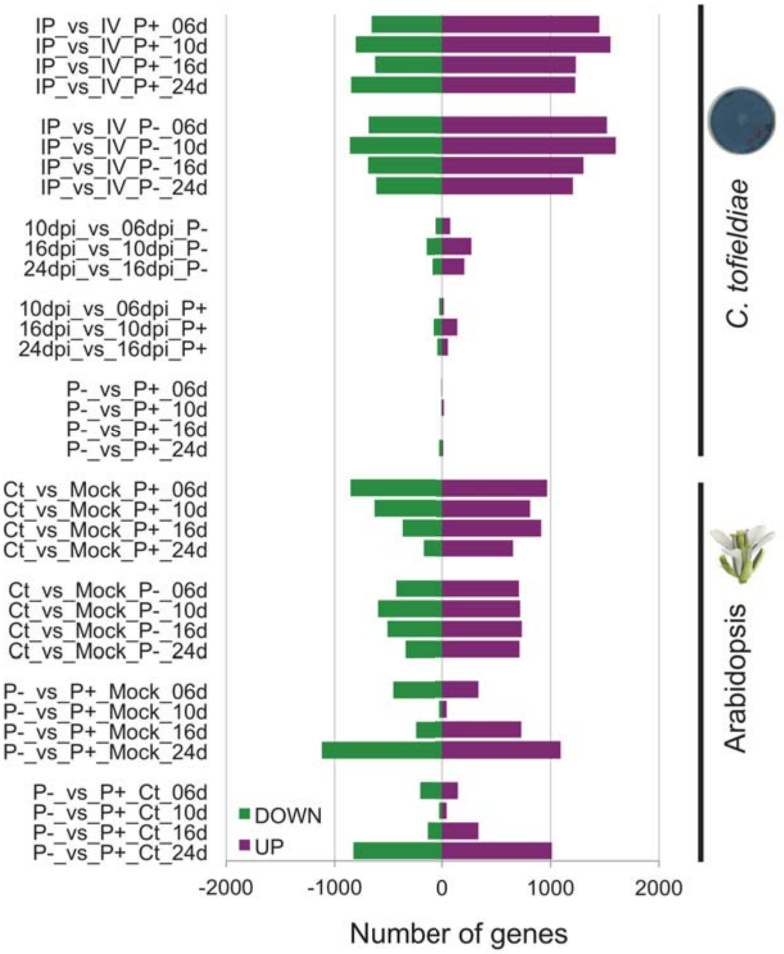

For both *C. tofieldiae* (upper part) and *Arabidopsis thaliana* (lower part), the number of genes identified as significantly regulated (|log2FC|≥1, FDR<0.05) between two conditions is represented (green: down regulated, violet: up-regulated). IP: *C. tofieldiae in planta*; IV: *C. tofieldiae in vitro*; P+: phosphate sufficient [625µM]; P-: phosphate deficient [50µM]; dpi: days post inoculation; *Ct*: *C. tofieldiae,* mock*:* control plants. Note: In contrast to the fungal transcriptome, the transcriptome of mock-treated plants is more responsive to the phosphate status. However, the host response to phosphate starvation is not visible at 24dpi in *C. tofieldiae* colonized roots, suggesting an active reduction of the phosphate stress by the fungus.

Supplementary Fig. 20: Expression profiling of 2,108 *C. tofieldiae* genes differentially regulated across any of the tested comparisons

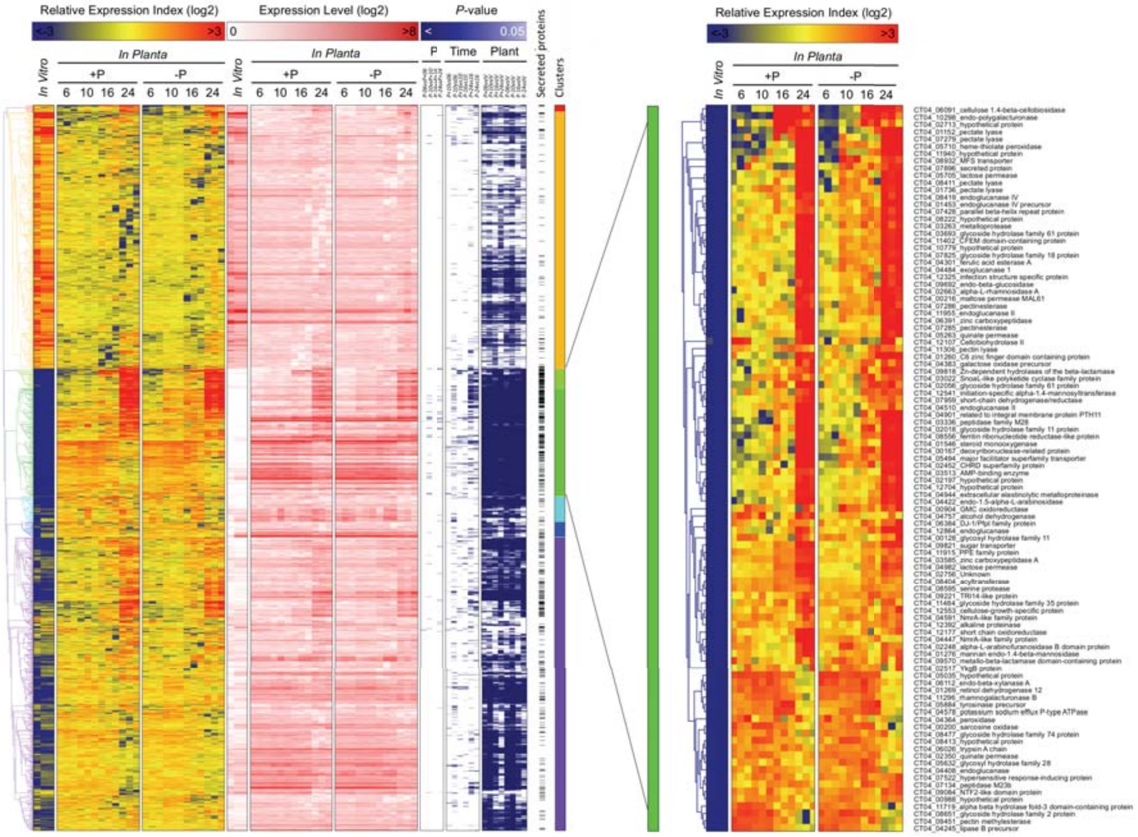

Heatmaps of gene expression showing the 2,108 most strongly and significantly regulated genes (|log2FC|≥2, FDR<0.05) identified across all tested conditions [phosphate conditions (P), across timepoints (Time), *in planta* vs. *in vitro* (Plant)]. Overrepresented (yellow to dark red) and underrepresented transcripts (yellow to dark blue) are shown as log2 fold changes relative to the mean expression measured across all nine stages (1 *in vitro* + 8 *in planta*). Additionally, gene expression levels (log2) are indicated by a white to red color gradient and FDR-adjusted *p*-values (FDR<0.05, blue color) are depicted for each comparison. On the right side of the heatmap, genes encoding secreted proteins are highlighted with black lines. Seven major clusters were identified after hierarchical clustering of gene expression data and the transcript profiles of the top 100 most highly induced genes *in planta* (green clusters) are marked on the right side, together with their corresponding annotation.

Supplementary Fig. 21: Expression profiling of *C. tofieldiae* genes encoding CAZymes, key secondary metabolism enzymes, CSEPs, secreted proteases and transporters

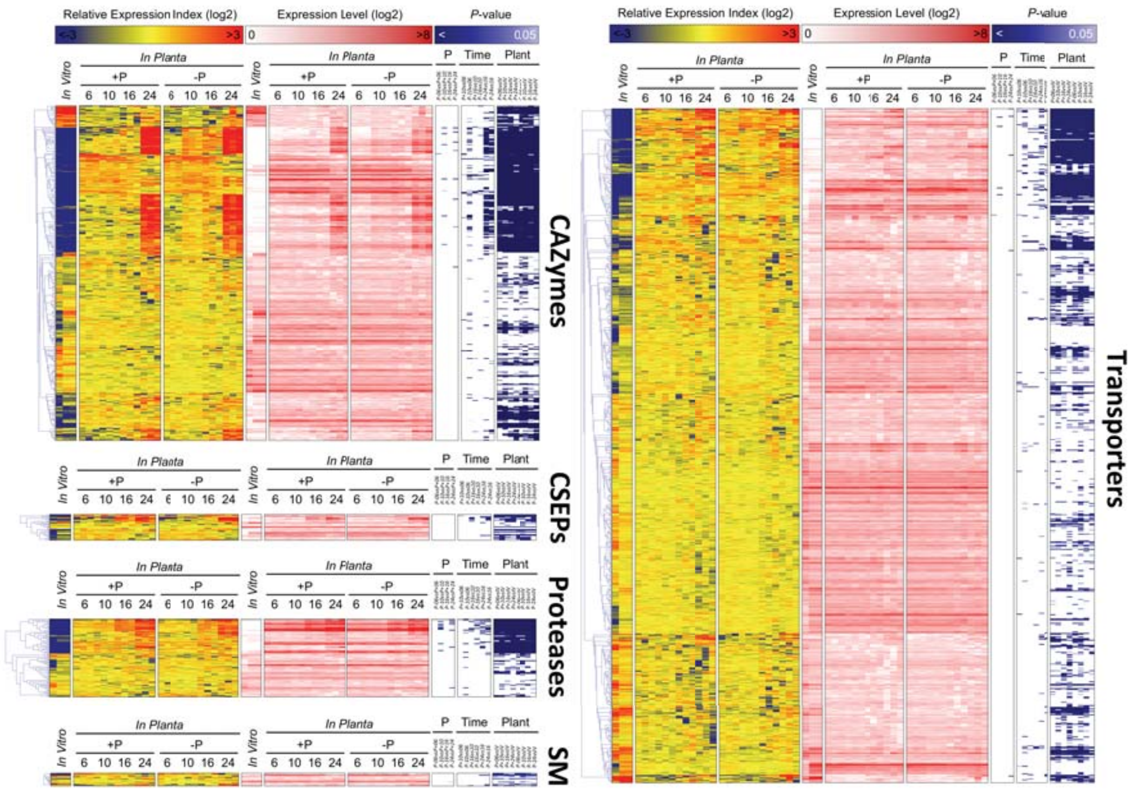

Heatmaps showing the expression profiles of *C. tofieldiae* genes belonging to five functional categories. Only genes with >100 reads in total across all *in planta* samples are presented. Overrepresented (yellow to dark red) and underrepresented transcripts (yellow to dark blue) are shown as log2 fold changes relative to the mean expression across all nine stages (one *in vitro* and eight *in planta*). Additionally, gene expression levels (log2) are indicated with a white to red color gradient and FDR-adjusted *p*-values (FDR<0.05, blue color) are shown for each tested comparison [phosphate conditions (P), across time points (Time), *in planta* vs. *in vitro* (Plant)].

Supplementary Fig. 22: Comparison of *Colletotrichum* genes encoding key secondary metabolism enzymes and their transcriptional regulation in *C. tofieldiae* and *C. incanum*

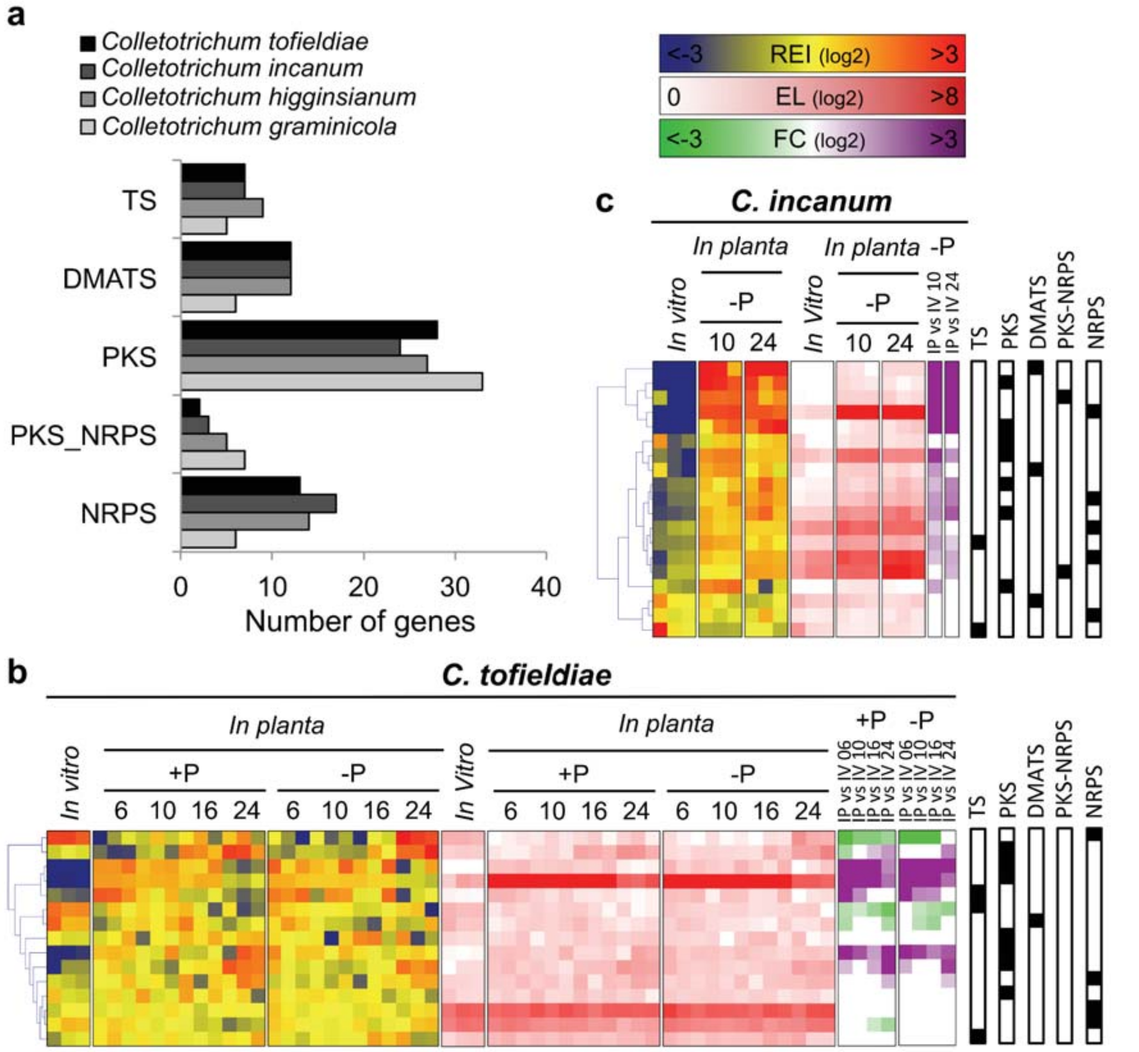

(a) Comparative analysis of the genes encoding key secondary metabolism enzymes in *Colletotrichum*. PKS: polyketide synthase; NRPS: nonribosomal peptide synthetase; DMATS: dimethylallyl tryptophan synthase; TS: terpene synthases ; PKS-NRPS: polyketide synthase-nonribosomal peptide synthase hybrids. Transcriptional regulation of the genes encoding key secondary metabolism enzymes are depicted in (b) for *C. tofieldiae* and (c) for *C. incanum.* Only the genes with >100 reads in total across all *in planta* conditions are presented (see methods). Overrepresented (yellow to dark red) and underrepresented transcripts (yellow to dark blue) are shown as log2 fold changes relative to the mean expression measured across all stages. Additionally, gene expression levels (log2) are indicated with a white to red color gradient and fold changes of significantly regulated genes (|log2FC|≥1, FDR<0.05); green: down-regulated; violet: up-regulated) measured between *in planta* (IP) and *in vitro* (IV) conditions in both phosphate sufficient (+P: 625μM) and deficient (-P: 50μM) conditions.

Supplementary Fig. 23: Transcript profiling of *Arabidopsis* genes annotated as chitin responsive and identification of key defense regulators through co-expression network analysis

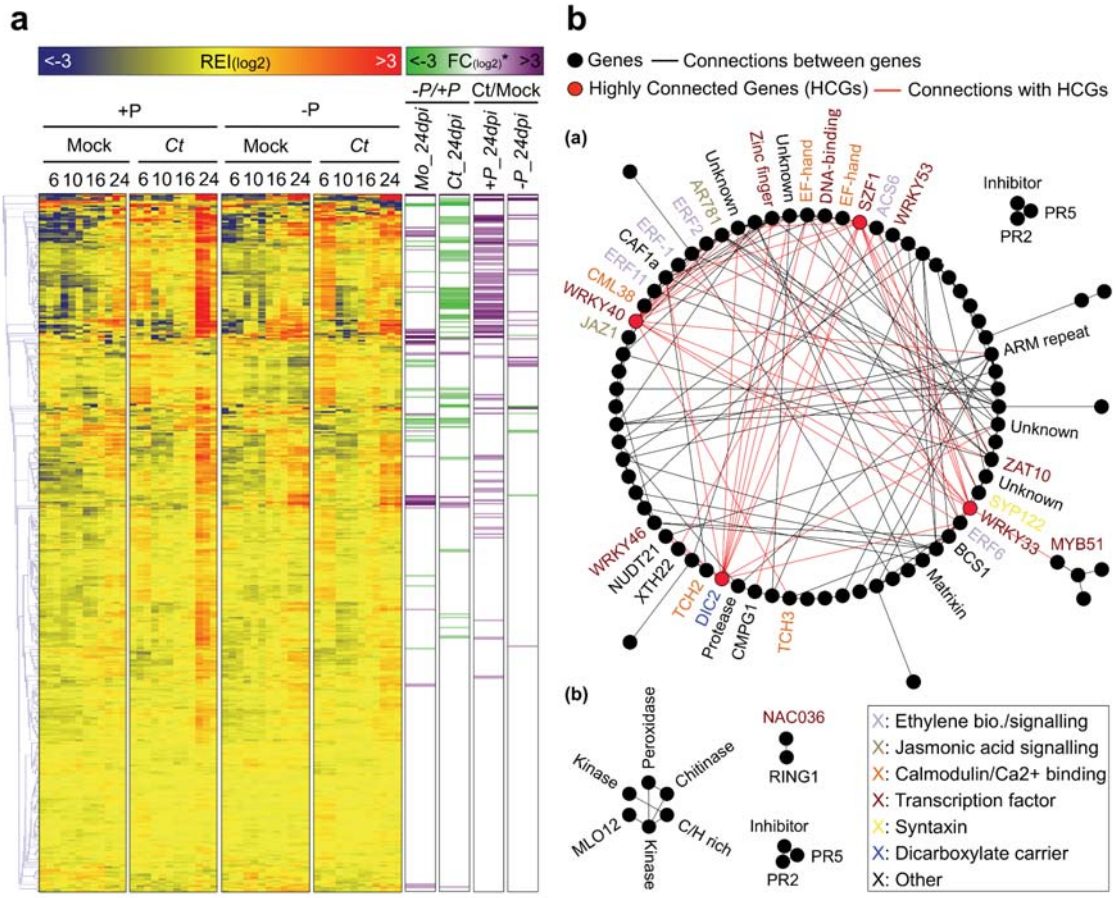

(a) Heatmap showing the expression profiles of 411 *Arabidopsis thaliana* genes annotated as chitin responsive based on the Gene Ontology Annotation database (http://www.arabidopsis.org/index.jsp). Overrepresented (yellow to dark red) and underrepresented transcripts (yellow to dark blue) are shown as log2 fold changes relative to the mean expression measured across all 16 stages. Fold changes of significantly regulated genes (|log2FC|≥1, FDR<0.05; green: down-regulated; violet: up-regulated) were measured between phosphate deficient (-P, [50μM]) vs. phosphate sufficient (+P, [625μM]) conditions in either mock control or *C. tofieldiae*-colonized plants as well as between *C. tofieldiae*-colonized vs. mock-treated plants in either phosphate-deficient (-P, [50μM]) or phosphate-sufficient (+P, [625μM]) conditions. (b) *A. thaliana* genes identified as significantly up-regulated in *C. tofieldiae*-colonized vs. mock-treated plants in phosphate-sufficient[(a)] and phosphate-deficient conditions[(b)] were selected for further gene co-expression network analysis and visualization. Genes that are also found to be co-regulated in other *Arabidopsis* expression datasets are likely to encode key regulators responsible for the remarkable transcriptional activation observed in *Ct*-colonized roots under phosphate-sufficient conditions. Four hub genes, showing a high degree of connectivity (>10 connections within the network) are represented with red circles[(a)]. The connections between these hub genes and the other genes within the network are depicted in red and the corresponding genes are indicated with different color codes corresponding to their respective functions. In contrast, only a few genes were identified as up-regulated in *Ct*-colonized roots under low phosphate conditions and these also do not show high connectivity among each other[(b)].

Supplementary Fig. 24: GO term enrichment analysis among significantly regulated *Arabidopsis* genes

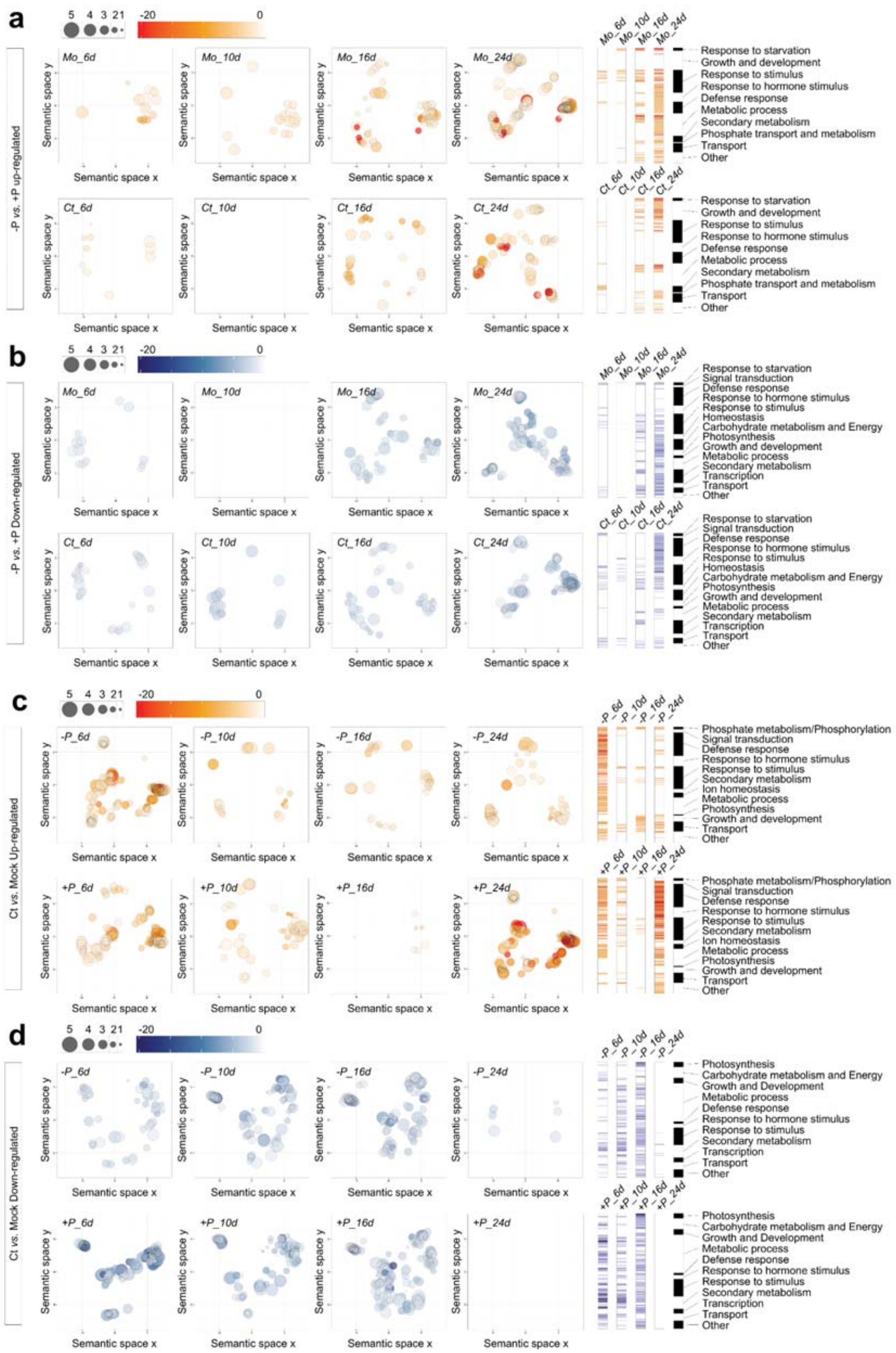

The GO term enrichment analysis was performed with AgriGO (http://bioinfo.cau.edu.cn/agriGO/) using the genes that were identified as significantly regulated ($|\log2FC| \geq 1$, FDR<0.05, see Supplementary Fig. 19) across the different tested conditions. The GO terms identified as significantly enriched (biological process, FDR<0.05) were visualized using ReviGO (http://revigo.irb.hr/). Enriched GO terms identified among significantly up- and down-regulated genes in phosphate deficient vs. sufficient conditions are depicted in (**a**) and (**b**), respectively. Enriched GO terms identified among significantly up- and down-regulated genes in *Ct*-colonized vs. mock-treated roots are depicted in (**c**) and (**d**), respectively. Each significantly enriched GO term is depicted as a circle. The size of the circle reflects the size of the GO term family (log2) and the color gradient indicates the log10-transformed *p*-values [white to red (up-regulated genes) or white to blue (down-regulated genes) color scales reflect significant (pale) to highly significant (dark) enrichments]. The GO terms are organized in a way that closely related GO terms cluster together in the semantic space[5]. On the right part of the figure, each enriched GO term identified using ReviGO is depicted as a line and is associated with a specific category. Note that GO terms associated with "defense response", "response to hormone stimulus" and "response to stimulus" are highly enriched in *Ct*-colonized roots under +P but not -P conditions at 24 dpi [see panel (**c**)].

Supplementary Fig. 25: Summary diagram illustrating the trade off between nutrition and immunity in roots in response to beneficial *C. tofieldiae* and pathogenic *C. incanum*.

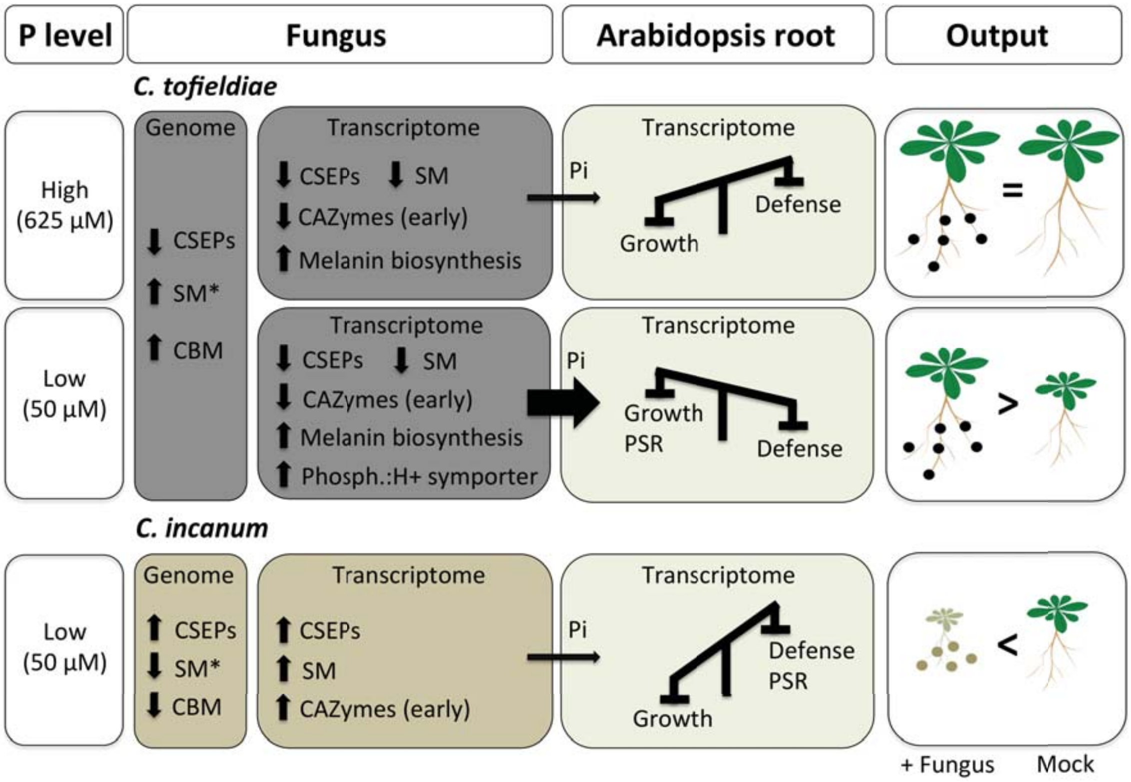

P level= phosphate level (KH_2PO_4), CSEPs= Candidate Secreted Effector Proteins, SM=Secondary Metabolism-related genes, CBM= Carbohydrate Binding Modules, CAZymes=Carbohydrate Active Enzymes, Pi=inorganic phosphate, PSR=Phosphate Starvation Response. * Based on genes families gained and lost during divergence from their common ancestor. However, secondary metabolism key gene repertories of *C. tofieldiae* and *C. incanum* are nearly identical in terms of total numbers and numbers in each category.

Supplementary Table 1: Background information regarding *Colletotrichum tofieldiae* and *Colletotrichum incanum* isolates selected in this study.

Isolate ID	Species	Accession Number[a)]	Location	Host plant	Publication
*Ct*0861	*C. tofieldiae*	Ct0861	Spain	*Arabidopsis thaliana*	Garcia et al. 2013
CBS127	*C. tofieldiae*	CBS 127615	Portugal	*Agapanthus* sp.	-
CBS130	*C. tofieldiae*	CBS 130851	Germany	*Semele gayae*	-
CBS495	*C. tofieldiae*	CBS 495.85	Switzerland	*Tofieldia calyculata*	-
CBS168	*C. tofieldiae*	CBS 168.49	Germany	*Lupinus polyphyllus*	-
Ci [b)]	*C. incanum*	MAFF 238704	Japan	*Raphanus sativus*	Sato et al. 2005

[a)] CBS: Culture Collection of the Centraalbureau voor Schimmelcultures, Fungal Biodiversity Centre, Utrecht, The Netherlands. MAFF: MAFF Genebank Project, Ministry of Agriculture, Forestry and Fisheries, Tsukuba, Japan;

[b)] *C. incanum* was originally identified as *C. dematium* by Sato *et al.*[6]. The correct identities was established in the present study (See Supplementary Fig. 2)

Supplementary Table 2: Genome sequencing and assembly statistics of *C. tofieldiae* isolates and *C. incanum.*

Input data & Assembly statistics	CBS495 *C.tofieldiae*	CBS130 *C.tofieldiae*	CBS127 *C.tofieldiae*	CBS168 *C.tofieldiae*	***Ct*0861** *C.tofieldiae*	***Ci*** *C. incanum*
Type of input data	Illumina	Illumina	Illumina	Illumina	Illumina (+454)	Illumina
Illumina read coverage	623x	287x	273x	305x	669x	231x
454 read coverage	-	-	-	-	14x	-
Assembly size	53.5 Mb	53.2 Mb	52.7 Mb	53.0 Mb	52.8 Mb	53.6 Mb
# of contigs	1,808	560	919	1,413	1,123	3,187
Largest contig	1.7 Mb	1.7 Mb	1.7 Mb	1.4 Mb	862 kb	1.1 Mb
Median length	1 kb	11 kb	0.4 kb	0.3 kb	1 kb	0.4 kb
Mean length	30 kb	95 kb	57 kb	37 kb	47 kb	17 kb
N50 length	245 kb	364 kb	377 kb	296 kb	209 kb	178 kb
N50	65	46	43	53	73	91
N75 length	114 kb	184 kb	210 kb	169 kb	121 kb	91 kb
N75	142	98	89	113	155	197
N90 length	40 kb	98 kb	94 kb	80 kb	62 kb	28 kb
N90	255	157	144	180	244	353
# of 'N's in assembly	15,073	321	298	395	538	6,251
Error free bases (Reapr)[a)]	95.22%	98.54%	97.67%	97.91%	96.74%	96.08%
mean read coverage by proper pairs (Reapr)[a)]	188x	266x	255x	282x	233x	205x
# of errors (Reapr)[a)]	3,778	228	229	238	7,127	458
# of warnings (Reapr)[a)]	135,548	1,311	1,892	2,443	1,431	5,566
# of Repeat Elements (PILER)[b)]	22	19	15	9	13	31
% of Genome Sequence (PILER)[b)]	0.57	0.79	0.74	0.65	0.64	1.42
% of Genome Sequence (Repbase)[b)]	0.68	0.58	0.87	0.80	0.79	1.68
# of predicted gene models[c)]	13,425	13,019	13,134	13,131	13,251	13,665
Gene space coverage (CEGMA)[d)]	97.18 - 98.79%	97.18 - 98.79%	97.18 - 98.79%	96.37 - 97.98%	97.58 - 98.79%	95.56 - 97.98%

[a] Assembly accuracy was evaluated based on mapped paired-end reads using Reapr (https://www.sanger.ac.uk/resources/software/reapr/). Listed in the table are the estimated fraction of error free bases, the underlying paired-end read coverage as well as the number of predicted assembly errors and other warnings.
[b] Repetitive DNA was identified in the genome assemblies using either a *de novo* approach (using PILER and PLAS[7]) or homology-based approaches (using the Repbase database[8])
[c] Gene models were predicted using the Maker pipeline (http://www.yandell-lab.org/software/maker.html).
[d] Gene space coverage was calculated using CEGMA (http://korflab.ucdavis.edu/datasets/cegma/). The two numbers indicate fully present and partially present genes.

Supplementary Table 3: Divergence date estimates.

Node[a)]	Calibration points[b)]	Age (Mya)
1	Pezizomycotina crown (*Paleopyrenomycites devonicus)*	400
2	Sordariomycetes crown (Secondary)	207 - 339
3	*Cordyceps - Metarhizium* divergence (Secondary)	146 - 206
	Estimated ages	
4	*Colletotrichum* crown	60.43
5	*C. incanum - C tofieldiae* divergence	8.80
6	*C. tofieldiae* crown	0.29

[a)] Correspond to the nodes presented in Fig. 1a: nodes 1-3 are calibration points (green in Fig. 1a) and nodes 4-6 represent divergence date estimates (red in Fig. 1a).

[b)] Node 1 was fixed to 400 Mya based on analysis of the Paleopyrenomycites fossil by Lucking *et al.*[9]. Node 2 was constrained to 339-207 Mya based on the phylogenetic analysis of Beimforde *et al.*[10] and Node 3 was constrained to 206-146 Mya based on the analysis of Sung *et al.*[11].

Supplementary Table 4: Summary statistics for whole genome assembly and short read alignments of *C. tofieldiae* isolates CBS495, CBS130, CBS127 and CBS168 to the reference genome assembly of *C. tofieldiae* 0861.

a) Summary statistics for MUMmerwhole genome alignments to the reference genome assembly of *C. tofieldiae* 0861.

	CBS495	CBS130	CBS127	CBS168
Aligned fraction of *Ct*0861 genome[a)]	93.4	94.2	95.3	95.2
Aligned fraction of other genome[b)]	94.0	94.9	97.3	96.5
Median length of aligned streches [bp][c)]	879	1,505	1,384	1,165
Average identity of aligned stretches[d)]	99.3	99.3	99.3	99.4

a) Fraction of the *Ct*0861genome assembly that was mapped in a MUMmer (nucmer) whole genome alignment to the assemblies of each of the other four isolates.
b) Fraction of each of the other four genome assemblies that was mapped in a MUMmer (nucmer) whole genome alignment to the reference genome assembly of *Ct*0861.
c) Median length of contiguous sequence stretches that could be aligned between the two assemblies by MUMmer (using default settings).
d) Average sequence identity between the two assemblies obtained from all aligned stretches (calculated as mean identity across all stretches, weighted by alignment length).

b) Summary statistics for short read alignments to the reference genome assembly of *C. tofieldiae* 0861.

Isolate	sequenced[a)]	aligned total[b)]		aligned concordant[c)]		aligned discordant[d)]		aligned orphan[e)]	
CBS495	387,374,222	350,202,494	90.4%	273,560,462	78.1%	63,474,106	18.1%	13,167,926	3.8%
CBS130	160,715,228	146,756,318	91.3%	118,391,844	80.7%	24,151,194	16.5%	4,213,280	2.9%
CBS127	152,026,788	143,440,974	94.4%	117,268,638	81.8%	21,875,930	15.3%	4,296,406	3.0%
CBS168	168,961,244	159,479,960	94.4%	130,264,044	81.7%	24,839,290	15.6%	4,376,626	2.7%

a) Number of 100bp paired-end Illumina reads that were sequenced (per isolate).
b) Total number and percentage of reads per isolate that aligned to the *Ct*0861 reference assembly.
c) Number and percentage of reads that aligned concordantly, i.e. both reads were aligned in the expected orientation and distance.
d) Number and percentage of reads that aligned discordantly, i.e. both reads were aligned, but not in the expected orientation or distance.
e) Number and percentage of reads that aligned as orphans, i.e. only one of the paired-end reads was aligned.

Supplementary Table 5: Overall number and density of single nucleotide polymorphisms (SNPs) in *C. tofieldiae* isolates CBS495, CBS130, CBS127 and CBS168 compared to the *Ct*0861 reference genome and average SNP density in low and high SNP density blocks.

Isolate	# of SNPs[a)]	SNP density per kb		
		Overall[a)]	High[b)]	Low[b)]
CBS495	144,602	2.74	4.88 ± 3.88	0.16 ± 0.14
CBS130	160,553	3.04	5.13 ± 4.02	0.32 ± 0.24
CBS127	140,799	2.66	5.04 ± 4.56	0.22 ± 0.17
CBS168	117,094	2.22	4.25 ± 3.68	0.25 ± 0.19

[a)] The overall number and density of SNPs was derived from the alignment of genome sequencing reads for each isolate to the *Ct*0861 reference assembly.
[b)] A two-component mixture model was used in combination with a hidden Markov model to predict blocks of high and low SNP density. Shown are the average SNP densities (± standard deviation) calculated for each isolate from all "high" and "low" density blocks, respectively.

Supplementary Table 6: Haplotype frequency and sequence conservation among *C. tofieldiae* isolates based on SNP density in 10 kb sliding windows.

Sequence conservation to other isolates[a)]	Haplotype[b)]	***Ct*0861** # Windows	%	CBS495 # Windows	%	CBS130 # Windows	%	CBS127 # Windows	%	CBS168 # Windows	%	Overall[c)] # Windows	%
Shared with all other isolates	H1	9,671	20.95	9,446	20.46	10,331	22.38	9,598	20.79	9,718	21.05	48,764	21.13
Shared with three other isolates	H2 - H6	8,016	17.37	10,850	23.51	9,778	21.18	9,365	20.29	6,923	15.00	44,932	19.47
Shared with two other isolates	H7 - H16	5,724	12.40	10,648	23.07	9,368	20.30	10,290	22.29	6,259	13.56	42,289	18.32
Shared with one other isolate	H17 - H26	9,068	19.65	8,304	17.99	6,705	14.53	6,873	14.89	8,786	19.04	39,736	17.22
Isolate-specific	H27 - H31	13,678	29.63	5,839	12.65	8,949	19.39	9,263	20.07	13,531	29.32	51,260	22.21
low coverage	LC	-	-	1,070	2.32	1,026	2.22	768	1.66	940	2.04	3,804	1.65

a) Sequence conservation in each 10 kb sliding window was assessed based on the SNP density in this window to each of the other isolates. The sequence in a window was assumed to be shared with another isolate, if the SNP density to the other isolate was classified as "low" in this window (using a two-component mixture model in combination with a hidden Markov model for prediction of low vs. high SNP density). If the SNP density to all of the other isolates was classified as high, the window was termed isolate-specific. If the average per nucleotide read coverage in this window was below 50 (CBS130, CBS127, CBS168) or 100 (CBS495) the window was termed low coverage (LC).

b) Based on the sequence conservation (low SNP density) to the other isolates, each window was assigend to one of 31 "Haplotypes" (H1-H31) in each of the isolates:

Sequence conserved among:	Haplotype	Sequence conserved among:	Haplotype
Ct0861, CBS495, CBS130, CBS127, CBS168	H1	Ct0861, CBS495	H17
Ct0861, CBS495, CBS130, CBS127	H2	Ct0861, CBS130	H18
Ct0861, CBS495, CBS130, CBS168	H3	Ct0861, CBS127	H19
Ct0861, CBS495, CBS127, CBS168	H4	Ct0861, CBS168	H20
Ct0861, CBS130, CBS127, CBS168	H5	CBS495, CBS130	H21
CBS495, CBS130, CBS127, CBS168	H6	CBS495, CBS127	H22
Ct0861, CBS495, CBS130	H7	CBS495, CBS168	H23
Ct0861, CBS495, CBS127	H8	CBS130, CBS127	H24
Ct0861, CBS495, CBS168	H9	CBS130, CBS168	H25
Ct0861, CBS130, CBS127	H10	CBS127, CBS168	H26
Ct0861, CBS130, CBS168	H11	Ct0861	H27
Ct0861, CBS127, CBS168	H12	CBS495	H28
CBS495, CBS130, CBS127	H13	CBS130	H29
CBS495, CBS130, CBS168	H14	CBS127	H30
CBS495, CBS127, CBS168	H15	CBS168	H31
CBS130, CBS127, CBS168	H16		

c) The overall number and percentage of windows per class were obtained by summing up the windows over all five isolates.

Supplementary Table 7: Number of genes that were annotated as secreted proteins and candidate secreted effectors (CSEPs) in the genomes of *C. tofieldiae* isolates, *C. incanum* and four other *Colletotrichum* species.

	C. tofieldiae					***C. incanum***	***C. higginsianum***	***C. graminicola***	***C. orbiculare***	***C. fructicola***
	CBS127	CBS130	CBS168	CBS495	*Ct*0861					
Proteome size	13,134	13,019	13,131	13,425	13,251	13,665	16,150	12,006	13,479	15,463
Secretome size[a)]	1,999	1,977	1,996	2,034	2,002	2,018	2,142	1,650	2,149	2,356
Percent of proteome	15.22	15.19	15.20	15.15	15.11	14.77	13.26	13.74	15.94	15.24
CSEPs[b)] genus-specific[c)]	105	105	101	101	99	117	136	77	169	134
CSEPs[b)] species-specific[c)]	28	24	27	24	34	72	196	69	171	101
CSEPs[b)] (total)	133	129	128	125	133	189	332	146	340	235
Percent of secretome	6.65	6.53	6.41	6.15	6.64	9.37	15.50	8.85	15.82	9.97

[a)] Secreted proteins were predicted using WoLF-PSORT (http://www.genscript.com/psort/wolf_psort.html). For the previously published species, if in the original publication a different method was used, to make datasets comparable we repeated the secretome prediction with WoLF-PSORT.

[b)] CSEPs were identified as predicted extracellular proteins with no significant BLAST homology to sequences outside the genus *Colletotrichum* in the UniProt database (both the SwissProt and TrEMBL components) using an e-value threshold of 1e-3.

[c)] The species-specific CSEPs lack homology to any proteins of other species. The genus-specific CSEPs have homology to other proteins only within the genus *Colletotrichum*. CSEPs were assumed to be species-specific only, if they had no Blast homology in UniProt and additionally also no orthologs were identified in the orthoMCL analysis.

Supplementary Table 8: Summary statistics of RNA-sequencing read alignment to *A. thaliana* (*At*) and *C. tofieldiae* (*Ct*) or *C. incanum* (*Ci*).

Library	Sample[a)]	Group[a)]	frags seq[b)]	algn At[c)]	algn At [%]	algn Ct/Ci[d)]	algn Ct/Ci [%]
917.A	plusP06mock_1	P+_d06_mock	21940671	17237772	78.6	2704	0.0
917.B	plusP06mock_2	P+_d06_mock	35571771	32178878	90.5	5436	0.0
917.C	plusP06mock_3	P+_d06_mock	33261068	29914479	89.9	4360	0.0
862.A	plusP10mock_1	P+_d10_mock	22428599	21067817	93.9	3813	0.0
862.B	plusP10mock_2	P+_d10_mock	21624627	20483777	94.7	3789	0.0
862.C	plusP10mock_3	P+_d10_mock	23741781	22559407	95.0	4613	0.0
1058.A	plusP16mock_1	P+_d16_mock	24173880	21921143	90.7	3077	0.0
1058.B	plusP16mock_2	P+_d16_mock	37232298	27465312	73.8	3395	0.0
1058.C	plusP16mock_3	P+_d16_mock	29759454	23782570	79.9	3014	0.0
862.AB	plusP24mock_1	P+_d24_mock	34042395	30401537	89.3	8364	0.0
862.AC	plusP24mock_2	P+_d24_mock	30427875	26817234	88.1	7658	0.0
862.AD	plusP24mock_3	P+_d24_mock	24078002	22106324	91.8	14340	0.1
917.D	plusP06Ct_1	P+_d06_Ct	26300725	25606857	97.4	187670	0.7
917.E	plusP06Ct_2	P+_d06_Ct	28021041	20869500	74.5	169479	0.6
917.F	plusP06Ct_3	P+_d06_Ct	33192134	30981251	93.3	274045	0.8
862.D	plusP10Ct_1	P+_d10_Ct	28915226	26880819	93.0	361107	1.2
862.E	plusP10Ct_2	P+_d10_Ct	25531103	23969668	93.9	250483	1.0
862.F	plusP10Ct_3	P+_d10_Ct	28344363	24542271	86.6	352261	1.2
862.M	plusP16Ct_1	P+_d16_Ct	29969823	23008276	76.8	112385	0.4
862.N	plusP16Ct_2	P+_d16_Ct	29011745	27424981	94.5	121170	0.4
862.O	plusP16Ct_3	P+_d16_Ct	28668453	24164996	84.3	81453	0.3
862.AE	plusP24Ct_1	P+_d24_Ct	27713094	25566990	92.3	81392	0.3
862.AF	plusP24Ct_2	P+_d24_Ct	32588965	30683785	94.2	135235	0.4
862.AG	plusP24Ct_3	P+_d24_Ct	34635716	31474402	90.9	85847	0.2
917.G	minusP06mock_1	P-_d06_mock	25823789	17854183	69.1	4106	0.0
917.H	minusP06mock_2	P-_d06_mock	23421116	15579066	66.5	3407	0.0
917.I	minusP06mock_3	P-_d06_mock	30519420	21516418	70.5	7455	0.0
862.G	minusP10mock_1	P-_d10_mock	24217016	22967251	94.8	4316	0.0
862.H	minusP10mock_2	P-_d10_mock	24323431	23020029	94.6	4718	0.0
862.I	minusP10mock_3	P-_d10_mock	24517904	23415529	95.5	4945	0.0
862.V	minusP16mock_1	P-_d16_mock	39593254	37374074	94.4	7325	0.0
862.W	minusP16mock_2	P-_d16_mock	35568678	26757722	75.2	4376	0.0
862.X	minusP16mock_3	P-_d16_mock	35600891	31024616	87.1	4820	0.0
862.AH	minusP24mock_1	P-_d24_mock	26794885	25324148	94.5	20557	0.1
862.AI	minusP24mock_2	P-_d24_mock	26083270	24792344	95.1	17739	0.1
862.AJ	minusP24mock_3	P-_d24_mock	22579234	20977953	92.9	13106	0.1
917.J	minusP06Ct_1	P-_d06_Ct	37535067	24953583	66.5	534717	1.4
917.K	minusP06Ct_2	P-_d06_Ct	29475369	25828848	87.6	295780	1.0
917.L	minusP06Ct_3	P-_d06_Ct	37184087	29737784	80.0	340950	0.9
862.J	minusP10Ct_1	P-_d10_Ct	27822765	25836934	92.9	517883	1.9

862.K	minusP10Ct_2	P-_d10_Ct	39856718	37007889	92.9	688094	1.7
862.L	minusP10Ct_3	P-_d10_Ct	36718156	34145265	93.0	652457	1.8
862.Y	minusP16Ct_1	P-_d16_Ct	44942979	36277723	80.7	142861	0.3
862.Z	minusP16Ct_2	P-_d16_Ct	31873227	23976387	75.2	82581	0.3
862.AA	minusP16Ct_3	P-_d16_Ct	34347126	20345284	59.2	105345	0.3
862.AK	minusP24Ct_1	P-_d24_Ct	40802776	38094205	93.4	144830	0.4
862.AL	minusP24Ct_2	P-_d24_Ct	42928988	39463283	91.9	147102	0.3
862.AM	minusP24Ct_3	P-_d24_Ct	33738271	31688813	93.9	193170	0.6
862.AN	Ct_invitro_2	Ct_invitro	20627375	114488	0.6	18818112	91.2
862.AO	Ct_invitro_3	Ct_invitro	29763764	116523	0.4	27201435	91.4
562.A	Ct_invitro_1	Ct_invitro	17717925	289562	1.6	17296627	97.6
1058.G	minusP10Ci_Exp1_1	P-_d10_Ci	36945485	9488153	25.7	150798	0.4
1058.H	minusP10Ci_Exp1_2	P-_d10_Ci	27173503	6641677	24.4	109420	0.4
1058.I	minusP10Ci_Exp1_3	P-_d10_Ci	28799602	8169516	28.4	144877	0.5
1292.A	minusP10Ci_Exp2_1	P-_d10_Ci	27570708	26276761	95.3	1119792	4.1
1292.B	minusP10Ci_Exp2_2	P-_d10_Ci	27522066	25723831	93.5	1625141	5.9
1292.C	minusP10Ci_Exp2_3	P-_d10_Ci	26410004	24881307	94.2	1329883	5.0
1292.D	minusP24Ci_Exp2_1	P-_d24_Ci	24940658	24515242	98.3	283925	1.1
1292.E	minusP24Ci_Exp2_2	P-_d24_Ci	23963661	23273119	97.1	526593	2.2
1292.F	minusP24Ci_Exp2_3	P-_d24_Ci	28482729	27848917	97.8	466185	1.6
1058.M	Ci_invitro_1	Ci_invitro	25158674	15450	0.1	23805425	94.6
1058.N	Ci_invitro_2	Ci_invitro	26007443	12994	0.0	24166058	92.9
1058.O	Ci_invitro_3	Ci_invitro	24889560	10686	0.0	23574242	94.7

[a)] For all *in planta* samples, *A. thaliana* Col-0 was used as host plant and grown under phosphate-sufficient (P-) or -deficient (P+) conditions. The plants were either mock-treated (mock) or inoculated with *C. tofieldiae* 0861 (*Ct*) or *C. incanum (Ci)* and samples were harvested at 6, 10, 16 or 24 days after infection. Additionally, for both Ct and Ci *in vitro* (in vitro) samples were collected. For each treatment group, three biological replicates were analyzed.

[b)] Samples were subjected to Illumina paired-end sequencing, where each read-pair represents one sequence fragment. All RNA-sequencing statistics in this table and all further analyses were based on the count of aligned fragments (frags), i.e. each mapped read-pair is counted once (independent of whether one or both reads in the pair were mapped).

[c)] RNA-sequencing reads were aligned to the *Arabidopsis* reference genome (http://www.arabidopsis.org).

[d)] RNA-sequencing reads were aligned to either the *C. tofieldiae Ct*0861 (*Ct*) or *C. incanum* (*Ci*) genome assemblies created in this study.

Supplementary Table 9: Selected genes and primers used for validation of RNAseq data by RT-qPCR. Reference genes that were used for RT-qPCR data normalization are highlighted in grey.

	Gene_ID	Gene_Name	Forward	Reverse	size (bp)	Cluster ID (Fig. 5a)
Arabidopsis thaliana	AT3G18780	Actin 2	ACCTTGCTGGACGTGACCTTACTGAT	GTTGTCTCGTGGATTCCAGCAGCTT	298	
	AT5G43360	Phosphate transporter 1;3	CATGATGATTCTCTGCTCAGTTGC	CAGATAGAGGGTAGTCACCT	128	1
	AT2G38940	Phosphate transporter 1;4	TCTCTTTCGGACATGAGCCA	GCGAAAATACCACCAGCCAT	199	2
	AT5G01220	Sulfoquinovosyldiacylglycerol 2	TGTTGAGCCTTCTCCCTTTGCC	TCAGGAACACCTTCATGTGTCGTC	123	2
	AT5G61650	CYCLIN P4;2	TGTTCGGAATTGGGTTTGAGT	TGCTTCTTGTTGTGGTGAGT	202	8
	AT5G67400	Root hair specific 19	CAGTTGACCCAAGAATCGCC	AAGCCTTGTTGAAAGCAACAGA	186	8
	AT4G02270	Root hair specific 13	GCAGGAAGGACAGTGACCAA	GTGCTCAATGGCTGTCCTCTA	110	8
	AT2G38470	WRKY DNA-binding protein 33	CTCAAGCACCATATACACTTCA	CCTTTGCTCTAGAGAATCCACC	144	9
	AT1G80840	WRKY DNA-binding protein 40	CTTGACTGTGCCGGTGACTA	GAAGAAGCCATTTGCTCCAC	117	9
	AT2G44840	Ethylene-responsive factor 13	CCGTCAGTCTCCGATCAGTT	GATCCACCGTGAAATCCAAC	103	9
	AT2G19190	FRK1	ATCTTCGCTTGGAGCTTCTC	TGCAGCGCAAGGACTAGAG	108	9
Colletotrichum tofieldiae	CT04_12898	Tubulin beta-1 chain	gcgggaaagtgtcaatgaag	gactctgcggaaactgttctg	191	
	CT04_05366	Phosphate:H+ symporter	ggacttcttccgccactaca	gcaaggacaaggatcaggttac	204	
	CT04_00229	Hypothetical protein (CSEPs)	gcatctgtgcctcgatctatt	atgttcgccacgttggtagt	245	
	CT04_09572	Hypothetical protein (CSEPs)	gccttttgggttctctgttg	tgatgtccatgtccgtatcc	176	
	CT04_02539	Thiamine biosynthesis protein	gcgtgactggaccaaggtta	ccacccttgcaagaaacctc	177	
	CT04_06091	Cellulose 1,4-beta-cellobiosidase	ggttgagtgcggcattaaca	gagcgtcgcagtaaccagta	119	
	CT04_02713	Hypothetical protein	ctcggcgagactgttgagat	tgctgattccgaggaccttg	121	
	CT04_07285	Pectate lyase (PL1)	gggaaggagatcaaggttgct	acggtgtctgcctggatac	185	
	CT04_04070	Beta-ketoacyl synthase (polyketide)	gctggattgagaaccctggata	tggccgtagaaggtaccaatg	187	
	CT04_01769	LysM domain-containing protein	gccgtactcaacccgctat	ctcgtcatccaggtgccata	180	
	CT04_07148	Hypothetical protein (CSEPs)	tgctctctcatatccagggaac	tgagaacaacacgccctgaa	183	

Supplementary Table 10a: GO term enrichment among *in planta* differentially expressed orthologous *C. tofieldiae* and *C. incanum* genes.

		GO Term[a)]	GO-ID	FDR[b)]	P-Value[b)]	# in test set[c)]	% of test set[c)]	# in ref set[d)]	% of ref set[d)]	# notAnnot in test set[e)]	# notAnnot in ref set[e)]
Up-regulated ***C. incanum***	10 dpi	oxidation-reduction process	GO:0055114	2.76E-07	2.12E-10	109	24.28	476	12.61	340	3300
	24 dpi	-	-	-	-	-	-	-	-	-	-
Up-regulated ***C. tofieldiae***	10 dpi	-	-	-	-	-	-	-	-	-	-
	24 dpi	melanin biosynthetic process	GO:0042438	4.65E-02	4.75E-05	3	1.95	0	0,00	151	4071
		phenol-containing compound biosynthetic process	GO:0046189	4.65E-02	4.75E-05	3	1.95	0	0,00	151	4071
		melanin metabolic process	GO:0006582	4.65E-02	4.75E-05	3	1.95	0	0,00	151	4071

a) Only results for GO terms belonging to the GO category 'Biological Process' (BP) are shown.
b) GO term enrichment analysis was performed using the Blast2GO java tool (with default settings) on the respective Blast2GO annotated *C. incanum* proteins in the orthologous gene pairs. To identify over-represented GO terms in a gene set, Fisher's exact test tis performed, and given here are the resulting uncorrected (P-Value) and the corresponding FDR-corrected (FDR) p-values obtained with this test.
c) Number (#) and percentage (%) of genes associated to each GO term in the test gene set (test set), i.e. in the set of significantly up-regulated genes.
d) Number (#) and percentage (%) of genes associated to each GO term in the reference gene set (ref set). As reference set, the set of all *C. incanum* genes with an unambiguous *C. tofieldiae* ortholog was used. To create a consistent contingency table for the statistical test, all genes included in the test set were removed from the corresponding reference set.
e) Number (#) of genes not associated to the respective GO term in the test and corresponding reference (ref) gene sets.

Supplementary Table 10b: Functional enrichment (based on GO terms and three additional pre-defined gene categories) among *in planta* expressed genes specific to *C. incanum* or *C. tofieldiae*

		GO Term[a)] or Gene category	GO-ID	FDR[b)]	P-Value[b)]	# in test set[c)]	% of test set[c)]	# in ref set[d)]	% of ref set[d)]	# notAnnot in test set[e)]	# notAnnot in ref set[e)]
Specific to *C. incanum*	GO terms	nucleic acid phosphodiester bond hydrolysis	GO:0090305	3.08E-02	2.94E-05	11	7.53	78	1.53	135	5036
		RNA-dependent DNA replication	GO:0006278	2.61E-02	1.87E-05	4	2.74	3	0.06	142	5111
	other categories	CSEP genes	-	4.68E-25	9.35E-29	36	5.94	19	0.24	570	7988
		secreted protein genes	-	ns	ns	98	16.17	1095	13.68	508	6912
		in planta induced genes	-	1,03E-05	2,06E-09	266	43.89	2557	31.93	340	5450
Specific to *C. tofieldiae*	GO terms	translation	GO:0006412	4.41E-02	1.38E-04	21	11.80	211	4.64	157	4335
		amide biosynthetic process	GO:0043604	4.21E-02	1.14E-04	22	12.36	224	4.93	156	4322
		respiratory electron transport chain	GO:0022904	4.21E-02	1.21E-04	4	2.25	4	0.09	174	4542
		peptide metabolic process	GO:0006518	4.02E-02	9.46E-05	22	12.36	221	4.86	156	4325
		peptide biosynthetic process	GO:0043043	2.72E-02	5.70E-05	22	12.36	213	4.69	156	4333
	other categories	CSEP genes	-	3.13E-06	6.27E-10	11	2.76	7	0.11	387	6288
		secreted protein genes	-	ns	ns	58	14.57	782	12.42	340	5513
		in planta induced genes	-	ns	ns	142	35.68	2170	34.47	256	4125

a) Only results for GO terms belonging to the GO category 'Biological Process' (BP) are shown.
b) GO term enrichment analysis was performed using the Blast2GO java tool (with default settings) on the respective Blast2GO annotated *C. incanum*/*C.tofieldiae* proteins. To identify over-represented GO terms/gene categories, Fisher's exact test is performed, and given here are the resulting uncorrected (P-Value) and the corresponding FDR-corrected (FDR) p-values obtained with this test.
c) Number (#) and percentage (%) of genes associated to each GO term/gene category in the test gene set (test set), i.e. in the set of C.incanum or C. tofieldiae specific (I.e. without ortholog in the other species) in planta expressed genes.
d) Number (#) and percentage (%) of genes associated to each GO term/gene category in the reference gene set (ref set). As reference set, the set of all in planta expressed C. incanum/C.tofieldiae genes with ortholog in the other species was used to create a consistent contingency table for the statistical test.
e) Number (#) of genes not associated to the respective GO term/gene category in the test and corresponding reference (ref) gene sets.

Supplementary Table 11: Levels of bioavailable phosphate in six Spanish soils where natural *Arabidopsis* populations colonized by *C. tofieldiae* were identified.

				C. tofieldiae **prevalence**[d)]		
SOIL [a)]	pH	SOM (%) [b)]	P (bioavailable, ppm) [c)]	2009-2010	2011-2012	Compiled
CDC	6.5	0.63	5.5	23%	24%	23%
MEN	5.2	2.45	7.9	63%	25%	52%
POL	6.6	1.85	3.8	31%	10%	26%
LRO	6.4	1.31	17.0	56%	65%	62%

a) The soils in MEN and POL sites were not deeper than 15 cm. Therefore, the same fraction was used for CDC and LRO to make the results comparable.
b) Soil Organic matter, Oxidation method.
c) Bioavailable Phosphorus (Olsen method[12]). <5 ppm, very poor; 5-15 ppm, poor; 15-30 ppm, normal.
d) Indicates the percentage of plants colonized by *C. tofieldiae* using qPCR as detection method[1].

Supplementary Note 1

Selection of isolates, colonization process and effect on plant growth

Colletotrichum tofieldiae isolate 0861 (*Ct*0861) was originally isolated from surface-sterilized *Arabidopsis* leaves collected from a natural population in Spain[13] (Supplementary Table 1). Despite being isolated from healthy leaves, repeated attempts to re-colonize *Arabidopsis* leaves under laboratory conditions were unsuccessful, even though *C. tofieldiae* differentiates black melanized appressoria that are well-known infection structures used by pathogenic *Colletotrichum* species to penetrate host epidermal cells[1]. In contrast, the fungus efficiently penetrates the rhizoderm of *Arabidopsis* roots using an appressorium-independent penetration strategy. Root penetration is followed by colonization of root tissues by both inter- and intracellular hyphae. Importantly, *C. tofieldiae* can be detected in *Arabidopsis* leaves 28 days after inoculation, indicating that the fungus can indeed colonize *Arabidopsis* leaves systemically without causing any disease symptoms[1]. Moreover, it was already shown that *C. tofieldiae* is a beneficial fungus that promotes *Arabidopsis* growth under phosphate-limiting conditions [50 µM] (Supplementary Fig. 1a, b), which was validated by ^{33}P-orthophosphate translocation experiments[1]. Notably, the plant growth-promoting activity is no longer detectable under phosphate-sufficient conditions [625 µM], indicating that beneficial effects on plant growth and development depend on host nutritional status[1]. Besides *C. tofieldiae* isolate 0861, we included four additional *C. tofieldiae* isolates in our analyses that were originally collected from widely separated geographical areas and different host plants (Supplementary Table 1). *C. tofieldiae* isolates CBS127 (full ID: CBS 127615) and CBS130 (full ID: CBS 130851) were isolated from the monocotyledonous plants *Agapanthus* in Portugal and *Semele gayae* in Germany, respectively. *C. tofieldiae* isolate CBS495 (full ID: CBS 495.85) was collected in Switzerland from the monocotyledonous plant *Tofieldia calyculata*, whereas isolate CBS168 (full ID: CBS 168.49) was obtained from the dicotyledonous plant *Lupinus polyphyllus* in Germany. To gain insight into the evolution from pathogenic to beneficial lifestyles in root-associated *Colletotrichum* fungi, we also selected *Colletotrichum incanum* (previously described as *C. dematium*[6] (see Supplementary Note 3), a closely related pathogenic species that was originally isolated from *Raphanus sativus* leaves in Japan (Supplementary Table 1). *C. incanum* has a very wide host range encompassing three plant families (Fabaceae, Brassicaceae, Solanaceae), and we showed that it can also actively colonize *Arabidopsis* roots under laboratory conditions. However, in contrast with its sister species *C. tofieldiae*, it has a deleterious impact on Pi-starved (Pi: inorganic phosphate) *Arabidopsis* plants[1] (Supplementary Fig. 1a, b). The close phylogenetic proximity between these two *Colletotrichum* species, together with their ability to colonize *Arabidopsis* roots but with

opposite outcomes for plant growth, provides a unique opportunity to unravel the molecular mechanisms underlying pathogenic and beneficial plant-fungal interactions.

Supplementary Note 2

Genome sequencing, assembly and annotation

High quality assemblies with high Illumina read coverage (231x-669x) were obtained for all isolates, as exemplified by the relatively small number of assembled contigs (560-3,187), their high N50 length (178-377 kb) and the fact that 97.98-98.79% of the core eukaryotic genes were detected by the CEGMA annotation pipeline (Supplementary Table 2 and Supplementary Methods). The assemblies have similar sizes, ranging between 52.7 and 53.6Mb, comparable to the genome size of other *Colletotrichum* species such as *C. fructicola* (previously named *gloeosporioides*) (55.6Mb), *C. graminicola* (50.9Mb) or *C. higginsianum* (49.3Mb) and also in the range of most other ascomycete fungal genomes sequenced so far[14-16]. Similar numbers of protein-coding genes were predicted in the five *C. tofieldiae* isolates (13,019-13,425) and in *C. incanum* (13,665) (Supplementary Table 2), suggesting that the transition from pathogenic to beneficial lifestyles is not associated with a major expansion or contraction of the total gene repertoire. With the exception of *C. incanum*, all of the genome assemblies contained less than 1% of repetitive DNA (Supplementary Table 2). These estimates are probably much lower than the true repetitive DNA content of the genomes, because the sequencing and assembly techniques used are likely to have excluded or collapsed highly repetitive sequences, resulting in their under-representation in the final assembled contigs. The *C. incancum* assembly contains nearly twice the percentage of repetitive DNA as the *C. tofieldiae* assemblies, suggesting an expansion of repetitive elements in this species. This is largely accounted for by a significant expansion of the Pyret element in *C. incanum* (459 elements, total length 400 Mb), a Ty3/Gypsy retrotransposon that was first identified in *Magnaporthe grisea*[17]. This suggests there has been a recent species-specific invasion of the *C. incanum* genome by this transposable element.

Supplementary Note 3

Phylogeny of ***Colletotrichum*** and divergence date estimates

Phylogeny of Colletotrichum. To position *C. tofieldiae* isolate 0861 and *C. incanum* within the context of the genus *Colletotrichum*, we first generated a phylogenetic tree based on whole genome sequencing data (Supplementary Fig. 2a and Supplementary Methods). The whole genome-based phylogenetic tree indicates a very short phylogenetic distance between *C. tofieldiae* isolate 0861 and *C. incanum* as well as their proximity to the maize pathogen *C. graminicola* (Supplementary Fig. 2a). Furthemore, to position the two species (including the four other *C. tofieldiae* isolates) studied here more precisely within the context of the genus *Colletotrichum*, we also generated a phylogeny based on sequences of six taxonomically informative loci (Supplementary Fig. 2b, c and Supplementary Methods). The analysis resulted in the detection of eight clades representing species within the *C. spaethianum* species complex (Supplementary Fig. 2b). Strain *Ct*0861 formed a clade with strains previously identified as *C. tofieldiae* that were congruent in morphology with the holotype of that species[18]. The isolates from Japanese radish (MAFF 238704, MAFF 238706, MAFF 238712, MAFF 238713) formed a clade together with *C. incanum* strains from *Solanum lycopersicum*, *Glycine max* and *Phaseolus vulgaris*, including the ex-holotype strain. The species *C. incanum* was recently described for the first time[19].

Divergence date estimates. Single-copy gene families, identified by clustering the predicted protein sequences from *C. incanum*, the five *C. tofieldieae* isolates and sixteen additional fungal species, were used to estimate (a) the age of the *Colletotrichum* crown, (b) the age of the common ancestor of *C. tofieldiae* and *C. incanum* and (c) the age of the *C. tofieldiae* crown (Fig. 1a and Supplementary Table 3 and Supplementary Methods). Based on this analysis, the age of the *Colletotrichum* crown was estimated to be 60.4 Mya, the age of the common ancestor of *C. incanum* and *C. tofieldiae* was estimated to be 8.8 Mya and the age of the common ancestor of the five C. *tofieldiae* isolates was estimated at 0.29 Mya (Fig. 1a and Supplementary Table 3). This divergence date between *C. tofieldiae* and *C. incanum* is comparable to the human-chimpanzee split that was estimated at 8 Mya[20]. Taken together, these results indicate that the beneficial lifestyle in *C. tofieldiae* is a rather recent adaptation arising from a more ancestral pathogenic lifestyle.

Supplementary Note 4

SNP distribution in *C. tofieldiae* isolates

The visualization of SNP locations and SNP densities revealed a strong heterogeneity in the SNP distribution, with regions of high SNP density alternating with regions of low density (Fig. 1b, Supplementary Table 5 and Supplementary Fig. 4a). Searching for possible causes underlying this heterogeneity, we calculated the GC content (%) and gene density (per 10 kb) in the *Ct*0861 genome based on the same sliding windows, but a comparison to the SNP density profiles did not reveal any striking correlations (Fig. 1c). We also checked the gene distribution between low and high SNP density regions based on pairwise comparisons between *C. tofieldiae* 0861 and each of the other four isolates. The proportion of genes in high (50.2%-58.8%) and low (41.2%-49.8%) SNP density regions does not deviate from the expected proportion based on the genome fractions (high SNP density regions: 49.9%-58.2%; low SNP density regions: 41.8%-50.1%) (chi-square *p*-values: 0.160-0.760). To determine whether low and high SNP density regions are enriched for specific genes, we additionally inspected the gene distributions in specific functional gene categories (secreted proteins, CSEPs, transporters, secreted proteases, see Supplementary Note 8). Although, we found a tendency that a larger proportion of CSEPs is located in high SNP density regions (Supplementary Fig. 4d), these regions account for such a large part of the genome (49.9%-58.2%) they most likely result from the exchange of genetic material between individuals rather than from 'two-speed' genome evolution[21]. Notably, the observed SNP density profiles overall are quite similar between the four analyzed isolates. Especially isolates CBS130 and CBS495 exhibit almost identical patterns, while isolate CBS168 and to a lesser extent isolate CBS127 show some distinct features (Fig. 1c and Supplementary Fig. 4b).

To further assess the sequence conservation among the five isolates, we extended our analysis and used the same sliding windows as before to extract for each *C. tofieldiae* isolate the SNP density relative to each of the other isolates (Supplementary Fig. 4c, Supplementary Methods). The SNPs between the 'non-reference' isolates were obtained based on comparisons of the respective SNP sets identified in comparison to *Ct*0861 using a custom R script. For each isolate the haplogroup in each window was then determined based on the SNP density with respect to the other isolates. In order to identify windows with a low SNP density (i.e. a common haplogroup) between isolates we classified the SNP density in each window as either 'low' or 'high' using a two-state hidden Markov model (HMM) (Supplementary Methods). Depending on which other isolates shared the haplogroup (i.e 'low' SNP density state) within a window, the window was then assigned to one of 31 possible haplogroups (H1-

H31) for each isolate (Supplementary Table 6). For example, if the SNP density to all other isolates was classified as ‘low’ in a window, the sequence was assumed to be conserved among all five isolates (H1). On the other hand if the SNP density to all other isolates was classified as ‘high’, the sequence was assumed to be isolate-specific (H27-H31). The other haplogroups correspond to cases where the sequence in a window was conserved among four isolates (H2-H6), three isolates (H7-H16) or two isolates (H17-H26). If the average read coverage per nucleotide in a window was below 50 (CBS130, CBS127, CBS168) or 100 (CBS495) the window was termed low coverage (LC) in the respective isolate. The detected haplogroup structure of each isolate was visualized in a heatmap, in which the color code represents with how many other isolates the haplogroup was shared within a window (Supplementary Fig. 4c). Based on this analysis, we also identified conserved regions on the *Ct*0861 contigs larger than 50 kb, where all five isolates are monomorphic (i.e., share one common haplogroup H1). For the five largest *Ct*0861 contigs these regions are highlighted in Fig. 1b.

Taken together, these data indicate that the *C. tofieldiae* isolates in our study show similar SNP density signatures. This pattern differs from the mosaic genome structure observed for the powdery mildew pathogen *Blumeria graminis*, where the analyzed isolates had more distinct signatures[22,23]. The alternation between low and high SNP density blocks likely reflects chromosome recombination events[22-25], suggesting that sexual reproduction can occur between different *C. tofieldiae* isolates (Fig. 1b and Supplementary Fig. 4a). However, the overall conserved SNP profiles, together with the large blocks of alternating low (median: 18-34 kb) and high (median: 11-44 kb) SNP density suggest *C. tofieldiae* reproduces almost exclusively asexually. If sexual reproduction would have been the major reproductive mode of *C. tofieldiae*, many more alternating blocks of smaller size should be visible and the SNP profiles would be more heterogeneous between isolates (see Fig. 1c and Supplementary Fig. 4b). Here, we showed that more than 20% of the haplogroups are conserved between all isolates and only 12-29% are isolate-specific (Supplementary Table 6 and Supplementary Fig. 4c). Given the fact that these isolates were collected from widely separated geographical areas, we conclude that *C. tofieldiae* isolates arose from only a small number of ancestral recombination events. These genomic rearrangements have been retained over evolutionary time and can still be detected in their genomes. Although mating type genes are present in the genomes of *C. tofieldiae* isolates, the striking conservation of SNP profiles suggests that *C. tofieldiae* reproduces mostly through clonal reproduction. Consistent with our results, *Colletotrichum* is traditionally recognized as an asexual genus and, with some notable exceptions (e.g. *C. graminicola* and *C. gloeosporioides*[26,27]), most *Colletotrichum* species reproduce asexually[28]. However, we cannot exclude the possibility that parasexual recombination and

exchange of genetic material, documented for some *Colletotrichum* species[29,30], can also contribute to the observed SNP signature.

Supplementary Note 5

Orthologous genes and multigene families

The gene repertoires of the five *C. tofieldiae* isolates and *C. incanum* were compared with those of four pathogenic *Colletotrichum* species for which genome sequences are available (i.e. *C. graminicola*, *C. higginsianum*, *C. orbiculare* and *C. fructicola*[14,15] (Fig. 2a). Gene families and clusters of orthologous genes were inferred using OrthoMCL v2.0[31] (Supplementary Methods). We observed a large 'core' genome with 7,297 gene families present in every analyzed *Colletotrichum* genome and a high similarity between all five *C. tofieldiae* isolates and C. *incanum*, with more than 10,300 gene families in common between these six genomes (Fig. 2a). When comparing the *C. tofieldiae* reference strain *Ct*0861 with *C. incanum*, we detected 10,519 gene families that were shared between both species, 1,486 *C. tofieldiae*-specific families and 618 *C. incanum*-specific gene families (Fig. 2b). Functional enrichment and overrepresentation analyses were performed among the *Ct*- and *Ci*-specific gene sets using a Fisher's exact test[32], adjusting for False Discovery Rate (FDR) using the Benjamini-Hochberg procedure with an alpha parameter of 0.05[33]. Interestingly, we found that protein-coding sequences related to biosynthesis of secondary metabolites were significantly enriched in the *Ct*-specific subset compared with the rest of the genome (p = 3.31e-08) (Fig. 2b). This result was validated using another approach based on ancestral genome reconstruction (see Supplementary Note 6).

Supplementary Note 6

Maximum likelihood ancestral gene family gain and loss inference

Ancestral genome reconstruction aims at predicting the DNA sequences of extinct ancestors in a given phylogeny according to a multiple sequence alignment of sequences at the leaves. Most often, the most likely estimate of the ancestral sequences is calculated, along with the most likely edge lengths[34]. In contrast to the general trend observed for the genus *Colletotrichum*, on the branch leading to *C. tofieldiae,* proportionally more gene families were gained (1,009 compared to only 198 lost gene families) than on other branches of the tree (*p*-value=3.98×10^{-136}; two-sided Fisher's exact test) (Supplementary Fig. 5a). In contrast, among gene families that were predicted to code for secreted proteins, there were significantly less gains on the branch leading to *C. tofieldiae* than on the other branches (p-value=9.12 × 10-5 two-sided Fisher's exact test after multiple testing correction, data not shown). More specifically, 19% (194/1,009) and 25% (49/198) of the families that were gained and lost, respectively, on the branch leading to *C. tofieldiae* encode secreted proteins. In comparison, on the branch leading towards *C. incanum*, 36% (118/331) of these gene families were gained and 28% (113/398) lost. We further investigated whether among the gene families gained or lost on the branch towards *C. tofieldiae* there was any enrichment of specific COG (Clusters of Orthologous Groups) functional categories. We found that significantly more gene families (75) were gained on this branch from the COG category 'Secondary metabolites biosynthesis, transport and catabolism' than on the other branches (p-value =5.89×10-3; two-sided Fisher's exact test after multiple testing correction) (Supplementary Fig. 5b). On the branch leading to *C. incanum* there were 30 gene families gained that were annotated with this COG category but no statistically significant functional enrichments were found. The significant enrichment of gene families related with secondary metabolite biosynthesis in the *Ct* genome is puzzling and suggests that acquisition of novel secondary metabolites or associated biosynthetic pathways may have facilitated the evolution toward beneficial *Ct*.

Supplementary Note 7

d_N/d_S analysis

The degree and direction of selective pressure acting on protein-coding genes can be assessed by comparing the rate of synonymous (silent) substitutions (d_S) - which are assumed to evolve neutrally - with the rate of non-synonymous substitutions (d_N)[35]. In the case of positive selection acting on protein-coding genes of a protein family, the ratio of d_N to d_S may be significantly larger than for protein families subject to purifying or no selection, indicating that diversification on the protein level provides a selective advantage to the organism, as commonly observed in evolutionary arms races between different organisms. This can be identified with statistical tests[36,37].

We calculated the ratio of the rate of mutations at non-synonymous sites to the rate of mutations at synonymous sites (d_N/d_S ratio) for each protein family identified from the five sequenced *C. tofieldieae* isolates and five pathogenic *Colletotrichum* species, namely C. *higginsianum*, *C. incanum*, *C. orbiculare*, *C. fructicola* and C. *graminicola* (Supplementary Data 2 and Supplementary Figs. 6 and 7). The d_N/d_S ratio for 331 putative candidate secreted effector proteins (CSEPs) of the ten *Colletotrichum* isolates was 0.35 ± 0.14, and these were significantly enriched in non-synonymous mutations relative to the non-CSEP families (mean d_N/d_S = 0.20 ± 0.13; one-sided Fisher's exact test, *p*-value < 2.2e-16) (Fig. 3b and Supplementary Fig. 6). Consistent with this result, we also found that *Colletotrichum* genes encoding small secreted proteins (SSPs) were significantly enriched in non-synonymous mutations (d_N/d_S = 0.22 ± 0.13) relative to the entire sample (one-sided Fisher's exact test, *p*-value < 2.2e-16) (Fig. 3b and Supplementary Fig. 6). This suggests that positive selection is acting on candidate effector genes in *Colletotrichum* and that these have diversified significantly more than other families, which supports the notion that members of these families are linked to pathogenicity of some of these species.

Further analysis of the selective pressure acting on the five pathogenic *Colletotrichum* species or on the five *C. tofieldiae* isolates revealed that protein families belonging to the functional categories “RNA processing and modification”, “defense mechanism” and “cell wall/membrane/envelope biosynthesis” were significantly enriched in non-synonymous mutations in the pathogenic species (Supplementary Data 2 and Supplementary Fig. 7a). In contrast, protein families of the functional categories “Signal transduction mechanisms”, “RNA processing and modification” and “Lipid transport and metabolism” had significantly larger numbers of nonsynonymous mutations in *C. tofieldiae* isolates (Supplementary

Data 2 and Supplementary Fig. 7b). This result indicates that proteins belonging to different gene categories show distinct evolutionary signatures in the pathogenic species compared to *C. tofieldiae*.

Analysis of d_N/d_S ratio also revealed that genes encoding proteins containing NACHT, HET, WD40 and NB-ARC domains are under positive selection in *Colletotrichum* pathogens, as well as in *C. tofieldiae* isolates (Supplementary Data 2). These proteins function as intracellular sensors that play a key role in fungal nonself recognition, and resemble the nucleotide-binding oligomerization domain (NOD)-like receptors (NLRs) controlling animal and plant innate immunity[38]. In fungi that are able to undergo spontaneous hyphal fusion with genetically distant genotypes, fusion can be inhibited by the heterokaryon incompatibility (HET) reaction that leads to programmed-cell death. This vegetative incompatibility correlates with the degree of pathogenicity of fungal species and their ability to form heterokaryons based on vegetative compatibility groups[39-42]. Fungal NLR proteins are highly polymorphic, with a repeat structure that can undergo combinatorial assortments[38]. The HET domain is an effector of the cell death reaction; the NACHT domain functions as a nucleotide binding domain, while the WD-repeat domain, comprising variable numbers of WD40 units, is required for protein-protein interactions with the antagonist partner[43].

Supplementary Note 8

Gene category descriptions

Secreted proteins. We identified 1,977-2,034 secreted proteins in the five *C. tofieldiae* isolates and 2,018 in the genome of *C. incanum*, accounting for 15.1-15.2% and 14.8% of the proteomes, respectively (Supplementary Table 7). To put these results into context, we compared the predicted secretomes of *C. tofieldiae* and *C. incanum* with those of the previously sequenced *Colletotrichum* species (*C. graminicola*, *C. higginsianum*, *C. orbiculare*, *C. fructicola*). Moreover, because *C. tofieldiae isolate* 0861 is endophytic on *A. thaliana*, colonizes plant roots and has plant growth-promoting activity, we additionally wanted to compare its secretome with those of other root-associated non-pathogenic or beneficial fungi. Due to the limited availability of proteome sequences of root-endophytic Ascomycota we chose to include the following species in our comparisons: *Laccaria bicolor* (Basidiomycota, ectomycorrhizal fungus)[44], *Piriformospora indica* (Basidiomycota, root endophyte)[45], *Rhizophagus irregularis* (Glomeromycota, arbuscular mycorrhizal fungus)[46], *Tuber melanosporum* (Ascomycota, ectomycorrhizal fungus)[47], *Harpophora oryzae* (Ascomycota, root endophyte)[48], *Epichloë festucae* (Ascomycota, leaf endophyte)[49], *Pestalotiopsis fici* (Ascomycota, branch endophyte)[50] and *Xylona heveae* (Ascomycota, sapwood endophyte)[51]. To ensure that the secretomes of all species were fully comparable we ran WoLF-PSORT on the proteomes of all 14 species. The analysis showed that the relative secretome sizes of the six *Colletotrichum* species are rather similar, ranging between 13.2% and 15.9% of the respective proteomes (Fig. 3a, Supplementary Table 7 and Supplementary Fig. 9a). The relative secretome sizes of the two endophytic ascomycete species *H. oryzae* and *P. fici* are also in that range, while the other endophytes and root mutualists generally have smaller secretomes, accounting only for 3.1% to 10.3% of their total proteomes (Supplementary Fig. 9a).

Candidate secreted effector proteins and small secreted proteins. Plant pathogens are known to secrete so-called effector proteins that promote virulence, eg. by interfering with host immune responses[52]. To compare the effector repertoires of beneficial *C. tofieldiae* with those of *C. incanum* and other pathogenic *Colletotrichum* species, we identified Candidate Secreted Effector Proteins (CSEPs) in all six species. CSEPs were defined as extracellular secreted proteins with no significant BLAST similarity (e value <1 × 10−3) to sequences outside the genus *Colletotrichum* in the UniProt database (SwissProt and TrEMBL components), as previously described[14]. We further distinguished two CSEP categories: the species-specific CSEPs that lack homology to any proteins of other species and the genus-specific CSEPs that have homology to other proteins only within the genus *Colletotrichum*. CSEPs were

assumed to be species-specific only, if they had no Blast homology in UniProt and additionally also no orthologs were identified in the orthoMCL analysis (see Supplementary Note 5). With this method we identified between 125 and 133 CSEPs in the five *C. tofieldiae* isolates and 189 CSEPs in *C. incanum* (Supplementary Table 7). Compared to the predicted CSEP repertoires of the other four *Colletotrichum* species, we noticed that, in spite of their comparable secretome sizes (see above), the *C. tofieldiae* isolates contain a markedly smaller fraction of CSEPs (Supplementary Table 7 and Supplementary Fig. 13). This difference is mostly due to a reduction of species-specific CSEPs, as in the *C. tofieldiae* isolates only 19-25% of the effectors are species-specific, compared to 38% in *C. incanum* and between 43% and 59% in the other four species. This contraction of the CSEP repertoire in *C. tofieldiae* is striking and suggests that the evolution of the beneficial from the ancestral pathogenic lifestyle was accompanied by a substential loss of species-specific CSEPs. A contraction of the predicted CSEP repertoire has also been reported in the genome of the symbiotic ascomycete fungus *T. melanosporum*, but not in the genomes of other mycorrhizal fungi sequenced to date[47,53]. This indicates that the loss of effector genes is one innovation among others that may favor the beneficial association. The contracted CSEP repertoire in *C. tofieldiae* could represent genomic signatures of the evolution from parasitism to endophytism in *Colletotrichum* and suggest *C. tofieldiae* does not require a diversified effector repertoire to invade its host. Alternatively, the fungus may have evolved alternative strategies to interfere with or evade the host immune system.

According to our CSEP definition, all predicted CSEPs are specific to the genus *Colletotrichum,* which makes a direct comparison to effectors in other genera very difficult. Yet, besides the genus-specificity, a small protein size and high cysteine content are assumed to be further typical characteristics of secreted effector proteins[54]. Such small-secreted proteins (SSPs) also are found in endophytic and mutualistic fungi[45,53] and are more comparable across genera. Therefore, to compare the predicted secretomes between the *Colletotrichum* species and the previously mentioned endophytic and mutualistic fungi in more detail, we separated the predicted secreted proteins according to their protein size (<300AA ⇒ 'small') and cysteine content (≥3% ⇒ 'high'). Generally, this analysis confirmed that the *C. tofieldiae* secretome is most similar to the secretomes of the pathogenic *Colletotrichum* species and of *H. oryzae* (Supplementary Fig. 9a). For the other endophytic and mutualistic fungi we observed that regardless of the smaller secretome size, the SSPs and cysteine-rich SSPs comprised a similar or in some cases even larger fraction of their secretomes compared to the *Colletotrichum* species. Consistent with the notion that a substantial fraction of the fungal secretomes comprises lineage-specific proteins, bi-directional BLAST searches (e-value $<1\times10^{-6}$) between *C. tofialdiae* isolate 0861 and the eight endophytic or mutualistic fungi identified a markedly smaller fraction of homologs among

the secreted and small secreted proteins than among the overall proteome (Supplementary Fig. 8). These BLAST searches also revealed that, both overall and among the (small) secreted proteins, the largest fraction of homologous proteins compared to *C. tofialdiae* isolate 0861 were found for the two endophytic Ascomycota *H. oryzae* (colonizes rice roots) and *P. fici* (colonizes tea branches) (Supplementary Fig. 8).

Secreted proteases. To identify *C. tofieldiae* and *C. incanum* genes encoding secreted proteases, sequences of predicted extracellular secreted proteins were subjected to MEROPS Batch BLAST analysis[55]. For comparison, we analyzed again the secretomes of four pathogenic *Colletotrichum* species (*C. graminicola*, *C. higginsianum*, *C. orbiculare*, *C. fructicola*) and eight endophytic or root mutualistic fungi (*L. bicolor*, *P. indica*, *R. irregularis*, *T. melanosporum, H. oryzae, E. festucae, P. fici, X. heveae*). As described previously[14], peptidase family S9 homologs were excluded from the final inventories because they are likely to be alpha/beta hydrolases (e.g. carboxylesterases and lipases) rather than proteases. We found that overall the repertoires of secreted proteases are relatively similar between *C. tofieldiae* 0861 (155) and *C. incanum* (135) but also among the other analyzed *Colletotrichum* species (108-156) (Supplementary Fig. 9b). The only major difference is a lower number of serine proteases in *C. graminicola*, which is mostly due to a marked reduction in the S08A family of subtilisin serine proteases. In contrast, the three mycorrhizal fungi (*T. melanosporum, L. bicolor, R. irregularis*) as well as three of the endophytic species (*P. indica*, *E. festuca*, *X. heveae*) display markedly smaller repertoires of nearly all classes of proteases (29-85), especially serine proteases, metallo proteases (with the exception of *P. indica*) and aspartic proteases (with the exception of *L. bicolor* and *P. indica*) (Supplementary Fig. 9b). The other two endophytes (*H. oryzae, P. fici*), in contrast, show relatively similar repertoires of secreted proteases compared to *C. tofieldiea* with only a slight reduction of metalloproteases and subtilisin serine proteases (especially in *P. fici*). The extensive repertoire of secreted proteases observed in *C. tofieldiae* indicates that contraction of proteolytic capability is not obligatory for the endophytic lifestyle and beneficial interaction with the plant host.

Transporters. To identify putative membrane transporter genes we performed BLAST searches against the Transporter Collection (TC) Database (http://www.tcdb.org) and extracted all genes with a sequence identity of at least 30% to their best hit in the database (e-value $<1\times10^{-3}$). These genes were subsequently assigned to the TC family of their best TCDB hit. In this way, we identified a total of 1,352 and 1,313 putative transporter genes in *C. tofieldiae* isolate 0861 and *C. incanum*, respectively (Supplementary Fig. 10). These numbers are comparable to those observed for *C. higginsianum* (1,233) and *C. fructicola* (1,204) with the same method, whereas in *C. graminicola* and *C. orbiculare* the number

of identified putative transporters was markedly smaller (963 and 999, respectively). In spite of this variation in overall numbers, the distribution of identified transporters into TC Sub-Classes and Families was very similar among all *Colletotrichum* species (including *C. tofieldiae* isolate 0861) but differed from that of other endophytes and mutualists (Supplementary Fig. 10). Generally, the three mycorrhizal fungi (*T. melanosporum, L. bicolor, R. irregularis*) and three of the endophytic species (*P. indica*, *E. festuca*, *X. heveae*) display relatively small transporter repertoires, while *P. fici* contains the largest repertoire of all analyzed species. Particularly, the number of genes belonging to the family 2.A (porters) is strikingly reduced in ectomycorrhizal fungi (*T. melanosporum* and *L. bicolor*), in the arbuscular mycorrhizal fungus *R. irregularis* and in the endophytic mutualist fungus *P. indica* but not in *C. tofieldiae* or the other endophytic species. In particular, the number of genes belonging to the Major Facilitator Superfamily (MFS, 2.A.1) ranged between 190 and 370 in the six *Colletotrichum* species and between 117 and 456 in the four endophytic Ascomycota, while only 34-58 were detected in the genomes of the three mycorrhizal fungi and *P. indica.* This marked disparity is largely explained by the reduced number of genes belonging to the Sugar Porter family (SP, 2.A.1.1), Drug:H+Antiporter families (DHA1: 2.A.1.2; DHA2: 2.A.1.3) and the Anion:Cation Symporter family (ACS: 2.A.1.14) in the three mycorrhizal fungi and *P. indica* (data not shown).

Secondary metabolism-related genes. The major classes of fungal secondary metabolites are synthesized by specialized, class-defining key enzymes, notably polyketide synthases (PKS), nonribosomal peptide synthetases (NRPS), hybrid PKS-NRPS enzymes, dimethylallyl tryptophan synthases (DMATS) producing alkaloids, and terpene synthases (TS). Using an in-house bioinformatic prediction pipeline (see Supplementary Methods), we compared the repertoires of SMKGs of *C. tofieldiae* isolate 0861 and *C. incanum* with those of four other *Colletotrichum* species pathogenic on leaves (*C. higginsianum*, *C. graminicola*, *C. orbiculare*, *C. fructicola*), three mycorrhizal fungi (*L. bicolor*, *T. melanosporum*, *R. irregularis*), a basidiomycete endophyte (*P. indica*) and four ascomycete endophytes (*H. oryzae*, *E. festucae*, *P. fici* and *X. heveae*).

The total numbers of SMKGs encoded by *C. tofieldiae* (62) and *C. incanum* (63), as well as the numbers in each major functional category, were nearly identical (Supplementary Fig. 11a). Overall, the large SMKG repertoires of *C. tofieldiae* and *C. incanum* resemble those of other *Colletotrichum* species, but DMATs are particularly well-represented in both *C. tofieldiae* (12) and *C. incanum* (12), as are PKS in *C. tofieldiae* (28) and NRPS in *C. incanum* (17). To assess the level of conservation of SMKGs between *C. tofieldiae* and *C. incanum*, we used orthoMCL protein clustering (see Supplementary Note 5) for the DMATS and TS, while for the other enzyme classes we built phylogenetic trees using

MEGA6[56] based on A domains (for NRPS) and KS-AT domains (for PKS and PKS-NRPS hybrids). Functionally characterised SMKGs from other ascomycetes were used as a reference dataset for protein alignments and tree-building[57,58]. In this way, we found that 20 and 19 SMKGs are specific to *C. incanum* and *C. tofieldiae*, respectively, while 43 SMKGs are shared by both species (Supplementary Fig. 11b).

In fungi, SM genes participating in the same pathway are typically located in clusters. One noteworthy secondary metabolism gene cluster that is present in both *C. incanum* and *C. tofieldiae*, but absent from any other *Colletotrichum* species sequenced so far, is homologous to the botrydial biosynthetic gene cluster. This was previously only reported in *Botrytis cinerea*, where it is responsible for the production of a non-specific phytotoxin belonging to the sesquiterpene class[3]. Homologs of all five genes of the *B. cinerea* botrydial cluster were identified in both *C. incanum* and *C. tofieldiae* using blastp (Blast+ v2.2.30 standalone program) with at least 77% amino acid identity (average 84%; Supplementary Fig. 11c). Additional genes encoding four putative tailoring enzymes, one ABC transporter and one transcription factor were also identified at this genomic location, which likely form part of the same SM gene cluster. Except for the ABC transporter, we also detected these additional genes in the genome of *B. cinerea* by blastp searches against the recently released gapless genome assembly of strain B05.10 (http://fungi.ensembl.org/Botrytis_cinerea/Info/Index). The high degree of conservation of this cluster in terms of gene content, sequence similarity and synteny is remarkable given the very large phylogenetic distance between *Colletotrichum* (class Sordariomycetes) and *Botrytis* (class Leotiomycetes), which diverged approximately 261.6 MYA (Fig. 1a). Although we cannot exclude the possibility that the botrydial cluster originated from a common ancestor and was subsequently lost from other ascomycetes, the most parsimonious conclusion is that the entire cluster was transferred horizontally from a putative donor and thereafter retained in certain *Botrytis* and *Colletotrichum* species through purifying selection[59].

Taking into account the wide variation in total proteome size among the ascomycete endophytes, *E. festucae* and *P. fici* encode relatively large SMKG repertoires similar to *Colletotrichum* species, whereas *H. oryzae* and *X. heveae* dedicate a much smaller proportion of their proteomes to SMKGs. Compared to the three mycorrhizal fungi and *P. indica*, which are also the most phylogenetically distant species, *C. tofieldiae* possesses a strikingly larger (4 to 21-fold) and more diverse SMKG repertoire (Supplementary Fig. 11a). The extremely reduced capacity for secondary metabolism in these four root-associated mutualists has been interpreted as an adaptation to avoid damage to the host plant by potentially toxic metabolites[45,46]. In the case of *C. tofieldiae*, damage avoidance may instead be achieved through the

transcriptional down-regulation of secondary metabolism genes. Accordingly, RNA-Seq transcriptome profiling revealed that only 15 of the SMKGs encoded by *C. tofieldiae* were actually expressed during root colonization, and only four of these were plant-induced genes (Supplementary Fig. 22). In contrast, *C. incanum* activated 19 SMKGs during infection and 14 of these were plant-induced. Among the pairs of orthologous SMKGs, three were differentially expressed between the two species, namely CT04_04263/CI_01566 encoding a TS, CT04_00232/CI_05288 encoding an NRPS and CT04_09174/CI_02165 encoding a PKS. The expression of the TS-encoding gene is plant-induced in *C. tofieldiae* and plant-repressed in *C. incanum*. Conversely, the PKS and NRPS pairs are plant-induced in *C. incanum* and plant-repressed in *C. tofieldiae*. In some fungi it has been shown that the presence of other microbes can activate numerous SM gene clusters and this is assumed for toxin producing endophytes to protect plants from other invaders[60,61]. However, in the gnotobiotic growth system used here for transcriptome analyses, such interactions were excluded. This could partially explain why the vast majority of SMKGs encoded by *C. tofieldiae* and *C. incanum* were silent during root colonization.

Carbohydrate active enzymes. To predict the repertoire of carbohydrate-active enzymes encoded by *C. tofieldiae* isolate 0861 and *C. incanum*, we scanned their genomes using the CAZy annotation pipeline[62,63] (http://www.cazy.org), which identifies putative Glycoside hydrolases (GH), glycosyltransferases (GT), polysaccharide lyases (PL), carbohydrate esterases (CE), redox enzymes with auxiliary activities (AA) and carbohydrate binding modules (CBM). We also used the same pipeline to annotate CAZymes in *C. higginsianum* and *C. graminicola*, the three mycorrhizal fungi *L. bicolor*, *T. melanosporum*, *R. irregularis* and the four plant-associated endophytes *P. indica, H. oryzae, E. festucae, P. fici, X. heveae* (Supplementary Fig. 12 and Supplementary Data 4).

Overall, the spectra of plant cell wall-degrading enzymes (PCWDEs) encoded by beneficial *C. tofieldiae* and pathogenic *C. incanum* are more similar to each other than to other pathogenic *Colletotrichum* species, reflecting their phylogenetic relatedness rather than their contrasting lifestyles. Both *C. tofieldiae* and *C. incanum* have extremely large arsenals of polysaccharide lyases, carbohydrate esterases and glycoside hydrolases acting on all the major plant cell wall components (pectin, hemicellulose and cellulose). Among root-associated mutualistic fungi, this pattern is more similar to orchid and ericoid mycorrhizae and some endophytes[53]. In contrast, the ectomycorrhizal fungi *T. melanosporum* and *L. bicolor* and the arbuscular mycorrhizal fungus *R. irregularis* all have extremely reduced sets of PCWDEs (Supplementary Fig. 12 and Supplementary Data 4), which appears to be a convergent evolutionary adaptation in mycorrhizal mutualists[44,46,47,53]. This contraction is also visible in

the genome of the fungal endophytes *X. heveae* and *E. festucae*, but not in the genomes of the root endophytes *C. tofieldiae*, *H. oryzae* and *P. indica*. The large degradative potential of *C. tofieldiae* is consistent with its observed capacity for necrotrophic growth on senescing plant tissues[1] and may also be required for saprotrophic growth on plant debris.

C. tofieldiae and *C. incanum* both encode more glycoside hydrolases (368 and 369, respectively) than *C. graminicola* (293), *C. orbiculare* (345) or *C. higginsianum* (357), with families of GH78 α-rhamnosidases (pectin degradation) and GH43 α-arabinosidases (hemicellulose and pectin degradation) notably expanded (Supplementary Fig. 12 and Supplementary Data 4). Those enzyme families targeting pectin (PL1, PL3, PL4, PL9, GH28, GH78, GH88, GH95, GH105, GH115, CE8 and CE12) are all inflated in the three Brassicaceae-infecting species (*C. tofieldiae*, *C. incanum* and *C. higginsianum*), in the Cucurbitaceae-infected species *C. orbiculare* and in the broad host range *C. fructicola* relative to the maize pathogen *C. graminicola* (Supplementary Data 4). This correlates well with the higher pectin content of dicot cell walls (~35%) compared to maize cell walls (~10%)[64]. Among those CAZymes having auxiliary activities, families of AA3_2 GMC oxidoreductases (involved in lignin degradation) are significantly larger in *C. tofieldiae* (49) and *C. incanum* (48) than in *C. higginsianum* (46), *C. orbiculare* (36) or *C. graminicola* (32) but comparable with the set predicted in *C. fructicola* (52). In contrast, the AA9 (formerly GH61) lytic polysaccharide monooxygenases are abundant in all six species, ranging from 32 in *C. incanum* to 25 in *C. higginsianum*.

The number of carbohydrate-binding modules encoded by *C. tofieldiae* (168) is strikingly higher than in the pathogenic *Colletotrichum* species *C. incanum* (143) *C. higginsianum* (117), *C. graminicola* (106), *C. orbiculare* (121) and *C. fructicola* (144). One common feature between *C. tofieldiae* and *C. incanum* is the high number of cellulose-binding CBM1 modules detected in both species (33) compared to the other *Colletotrichum* species (17-21). The high number of CBM modules detected in *C. tofieldiae* is mostly due to the abundance of chitin-binding CBM18 (48 vs. 28-40) and CBM50 (57 vs. 30-54) modules (Supplementary Data 4). In this respect *C. tofieldiae* resembles two other mutualistic root endophytes, *P. indica* and *H. oryzae*, which likewise encode numerous CBM50 proteins[45,48] (Supplementary Fig. 12 and Supplementary Data 4). Such proteins may be important for these fungi to conceal chitin in their cell walls from recognition by host immune receptors[65]. Despite their very similar CAZyme arsenals, *in planta* activation of these genes is strikingly different in *C. tofieldiae* and *C. incanum* (Fig. 4b, c). The more limited transcriptional reprogramming of CAZyme genes in *C. tofieldiae*

during early root colonization (<16 days) might be important for establishing and sustaining the beneficial relationship by avoiding cell wall-associated damage (see Supplementary Note 9).

Supplementary Note 9

Whole-genome transcriptome profiling

I- Analysis of the fungal transcriptomes

1- ***Most abundant fungal transcripts during in planta colonization.***

C. tofieldiae: In total, between 81,392 and 688,094 reads (*in planta* samples) could be mapped to predicted *C. tofieldiae* isolate 0861 gene models (Supplementary Table 8). This number of reads represents between 0.2 and 1.9% of the total number of sequenced Illumina reads per sample and reflects the relatively low fungal versus plant biomass, for which the number of reads represents >85% of the total (Supplementary Table 8). In order to avoid normalization biases due to undetectable transcripts and low expressed genes, we decided to apply a stringent cut-off by only selecting the genes that accumulate >100 reads across all 24 *in planta* conditions (Supplementary Fig. 17). Using this cut-off, we identified 6,693 *C. tofieldiae* genes expressed *in planta* that were used for further analyses. Among the top 100 most highly expressed genes across all *in planta* conditions, a large proportion (>33%) encodes secreted proteins, including 6 CAZymes acting on the fungal cell wall (GH16, GH17, GH18, GH55, GH72 and GH72-CBM43 families) and 4 acting on the plant cell wall (CE5, GH7, GH7-CBM1 and PL1_4 families). The number of *C. tofieldiae* CAZyme genes detected among the top 100 most highly expressed genes *in planta* is twice that detected for mycelium grown *in vitro* (35 vs. 18), suggesting those CAZymes are critical for fungal accommodation inside the root cell.

C. incanum: Between 283,925 and 1,625,141 reads (*in planta* samples) could be mapped to *C. incanum* gene models, representing between 1.1% and 5.9% of the total number of sequenced reads per sample (Supplementary Table 8). This higher percentage of detectable fungal reads compared to *C. tofieldiae* already suggests that root colonization by pathogenic *C. incanum* is faster than root colonization by beneficial C*. tofieldiae* (Supplementary Fig. 1c). Using *C. tofieldiae* and *C. incanum* transformants expressing GFP, we clearly demonstrated the much more extensive colonization of root tissues by *Ci* at this at 7 dpi (Supplementary Fig. 1d). Similar to *C. tofieldiae*, low expressed genes were removed, leading to a total number of 8,613 *in planta*-expressed genes. By looking at the 100 most highly expressed genes *in planta* (average expression level between 10 and 24 dpi), a similar signature was found as for *C. tofieldiae*. However, in contrast to the beneficial fungus, three *C. incanum CSEPs* ranked among the top 100 most highly expressed genes *in planta*, including two very highly expressed

CSEPs (3rd: CI_08930, 13th: CI_11610). This difference is even more pronounced when looking at the top 1,000 most highly expressed genes at 10 dpi, where only two *CSEPs* were detected for *C. tofieldiae* but 14 for *C. incanum* (data not shown). Notably, the most highly expressed *C. incanum* CSEP gene (10 dpi, CI_08930) showed a >160-fold transcript induction compared to its orthologous gene in *C. tofieldiae* (CT04_10683). Further inspection of all orthologous CSEP genes revealed a similar trend (9 out of 10 orthologous pairs), characterized by a significantly larger *in planta* accumulation of CSEP transcripts in *C. incanum* compared to its sister species *C. tofieldiae* (Supplementary Data 6)

2- *Significantly regulated fungal genes during colonization*

To unravel the genetic programs associated with each colonization stage of *C. tofieldiae* and *C. incanum* on *Arabidopsis* roots, pair-wise expression ratios were calculated (see Supplementary Data 5 for *C. tofieldiae* and Supplementary Data 7 for *C. incanum*).

C. tofieldiae: We first compared gene expression levels between *in planta* samples (6, 10, 16 and 24 dpi) and *in vitro* samples. For each comparison, >1,200 genes were significantly up-regulated (FDR<0.05; log2FC≥1) *in planta*, while fewer genes (615-861) were significantly down-regulated *in planta* (FDR<0.05; log2FC≤-1) (Supplementary Fig. 19). Although *in vitro* and *in planta* samples are not fully comparable due to the use of different nutritient media, it appears that >80% of the overall regulated genes are induced upon host contact (Supplementary Figs. 19 and 20). Interestingly, the number of significantly regulated genes drops considerably when looking at genes that are significantly activated/repressed over time (i.e 10 vs. 6 dpi, 16 vs. 10 dpi or 24 vs. 16 dpi). Indeed, the number of up- and down-regulated genes is lowest when comparing 10 vs. 6 dpi (+P conditions: 47 genes, -P condition: 137 genes), reaches a maximum for the comparison between 16 and 10 dpi (+P: 231 genes; -P: 422 genes) and decreases when comparing the last two time-points (i.e; 24 vs. 16 dpi; + P: 99 genes; -P: 302 genes). This result contrasts with the highly dynamic genetic program deployed by *C. higginsianum* during *Arabidopsis* leaf infection. Despite the shorter time period (22-60 hours post inoculation) and the more stringent cut-off used to define significantly regulated genes in *C. higginsianum* (FDR<0.05; |log2FC|≥2), more than five times as many genes were identified as differentially regulated between timepoints during colonization of *Arabidopsis* leaves by *C. higginsianum* compared with colonization of *Arabidopsis* roots by *C. tofieldiae*[14]. This result is striking and indicates that *C. tofieldiae* does not undergo a major transcriptional reprogramming during host invasion. Although *Arabidopsis* roots respond massively to phosphate deprivation with the transcriptional reprogramming of >2,000 genes at 24 dpi (Supplementary Fig. 19), the *C. tofieldiae* transcriptome

remains mainly unchanged with only 61 genes significantly regulated between -P and +P conditions during colonization of *Arabidopsis* roots. Among these, a gene encoding a phosphate H+ symporter (CT04_05366; FDR<0.05, log2FC=3.5) showed the strongest induction in phosphate deficient conditions at 24 dpi, indicating that the fungus can nonetheless activate dedicated transporters to promote phosphate uptake *in planta* (Supplementary Data 5). Orthologous genes were detected in the genomes of pathogenic *Colletotrichum* species including *C. higginsianum*, *C. graminicola* and *C. incanum*, indicating this phosphate H+ symporter is not a species-specific innovation. However, this transporter might play a critical role for the observed plant growth promoting activity since the increase of both root and shoot biomass is only detectable in phosphate deficient conditions[1].

C. incanum: We again compared gene expression levels between *in planta* (10 and 24 dpi) and *in vitro* samples. In total, twice as many genes were significantly up- (>2,100) and down-regulated (>1,500) *in planta* vs. *in vitro* (10 dpi or 24 dpi) than in *C. tofieldiae*. This is consistent with the fact that the genetic program deployed *in planta* by the pathogenic *C. incanum* is much more dynamic than the transcriptional reprogramming observed during root colonization by the beneficial fungus *C. tofieldiae*. The three genes showing the strongest *in planta* induction at 10 and 24 dpi in *C. incanum* (log2FC>13) encode a PPE family protein (CI_11017), an isoflavone reductase family protein (CI_10399) and a linoleate diol synthase (CI_04352) (Supplementary Data 7). Importantly, the corresponding orthologous genes in *C. tofieldiae* show a much weaker induction *in planta* (Supplementary Data 6). The top 10 *C. incanum* genes that are significantly up-regulated *in planta* at 24 dpi (FDR<0.05) also include six CAZymes acting on the plant cell wall, with four acting on pectin (PL3_2 and GH28 families) and two on cellulose (GH7 and AA9 families). The orthologous CAZyme genes were detected among the most highly induced genes in *C. tofieldiae* at 24 dpi under the same nutrient condition (Supplementary Data 5), suggesting that a similar cocktail of lytic enzymes are released by *C. tofieldiae* and *C. incanum* at late stages of root colonization.

3- *Gene expression profiling in C. tofieldiae*

To unravel the dynamics of *C. tofieldiae* transcriptional reprogramming during colonization of *Arabidopsis* roots, we scrutinized the transcripts profiles in gene categories required for fungal infection on plants including CSEPs, secreted proteases, transporters, CAZymes and SMKGs.

CSEPs: Impressively, among the 133 CSEP genes predicted in the genome of *C. tofieldiae*, only 18 were actually expressed *in planta* (Fig.3c and Supplementary Data 5). Although most of these were

induced at least at one time-point during root colonization at 6, 10, 16 or 24 dpi compared with *in vitro* samples, only 6 (CT04_00229, CT04_01748, CT04_04758, CT04_09572, CT04_10683, CT04_11158) show a strong *in planta* induction (log2FC>3), with only one species-specific *CSEP* (CT04_04758) (Supplementary Data 5). Furthemore, the dN/dS ratio for *in planta* expressed *CSEPs* was 0.40 ± 0.15 for *Ct* and 0.36 ± 0.14 for *Ci*, not significantly different from non-expressed *CSEPs* (0.35 ± 0.11 for *Ct* and 0.35 ± 0.14 for *Ci*) (*t*-test *p*=0,3555 for *Ct* and 0,644 for *Ci*). We can therefore exclude the possibility that plant-expressed *CSEPs* are more under diversifying selection than the other *CSEPs*. We also checked whether these *in planta* expressed *CSEPs* harbour similar number of cysteine residues and found that only for *Ct*, *in planta* expressed *CSEPs* encode proteins with less cysteine residues (cysteine content=2.11% ± 1.97) compared to non-expressed CSEPs (Cysteine content =3.49 ± 2.94 (*t*-test *p*=0,01572) (see Supplementary Data 3). Importantaly, none of these *in planta* expressed *CSEP* genes are differentially regulated according to phosphate status and only five are differentially regulated over time. In contrast to other plant-interacting fungi, this result indicates that *C. tofieldiae* does not sequentially deliver different suites of effectors during host colonization[14,54,66-69]. The very restricted set of CSEP genes expressed during root colonization, together with the very small number of these that are highly induced *in planta* indicates that *C. tofieldiae* requires extremely few effectors for host invasion and maintenance of the beneficial lifestyle. Alternatively, this could also reflect the very broad host range of *C. tofieldiae* (monocots and dicots), with different sets of CSEPs potentially activated during interaction with different hosts.

CAZymes: RNA-Seq expression profiling of *C. tofieldiae* genes encoding CAZymes revealed that 398 were expressed and two third of these were significantly regulated during colonization of *Arabidopsis* roots (Fig. 4b, Supplementary Figs. 20 and 21), with two distinct phases of activation. A relatively small number of genes encoding PCWDEs were induced at early stages of colonization (6-16 dpi) and repressed at 24 dpi (Fig. 4b). These include four genes encoding PCWDEs acting on hemicellulose (CT04_07791_GH11, CT04_03808_GH29, CT04_11299_GH10, CT04_12639_CE1), two acting on pectin (CT04_07564_GH28, CT04_09451_CE8) and two acting on hemicellulose or pectin (CT04_00209_GH43, CT04_04965_GH43). Interestingly, a similar induction of CAZyme genes acting on hemicellulose and cellulose was found during the first step of root cell colonization by the ectomycorrhizal fungus *L. bicolor*. These CAZymes are likely important for fungal penetration and remodeling of the plant cell wall through loosening of the cellulose chains[70]. Consistent with the less pronounced transcriptional reprogramming of CAZyme genes during early stages of colonization, ~5 times less CAZymes ranked among the most highly regulated genes (log2FC>10) at 6 dpi (seven CAZyme genes, -P conditions) than at 24 dpi (34 CAZyme genes, -P conditions) compared with the *in*

vitro stage (Supplementary Data 5). This indicates that *C. tofieldiae* secretes a narrower arsenal of PCWDEs during early colonization, probably to minimize damage to plant cells and prevent recognition by host immune receptors. A second wave of gene activation occurred between 16 and 24 dpi, associated with the induction of many more PCWDEs acting on all major wall polymers, including cellulose, hemicellulose and pectin (Fig. 4b). At 24 dpi, 84 of these genes were massively induced, including 26 encoding PCWDEs acting on cellulose, 16 acting on hemicellulose, 19 acting on hemicellulose or pectin and 23 acting on pectin. Consistent with this, more than 50% of the most highly up-regulated genes between 16 and 24 dpi (in both +P and -P conditions) encode CAZymes (Supplementary Data 5). A similar activation pattern was also observed at 24 dpi for the genes encoding secreted proteases (Supplementary Fig. 21). This suggests that a major shift in fungal lifestyle, involving increased degradation of plant cell walls, occurs at later stages of colonization (i.e. 24 dpi). Conceivably this is linked to the normal developmental process of cortical senescence that occurs in mature regions of the root system, enabling *C. tofieldiae* to grow saprotrophically on the dead or dying plant tissue. Importantly, *Ct*-colonized plants are still perfectly healthy at 24 dpi, suggesting this extensive activation of PCWDE genes does not impact host fitness.

Transporters: Among the 795 *C. tofieldiae* transporter genes considered as expressed, 471 were differentially regulated between at least one *in planta* sample and the *in vitro* samples, 119 were differentially regulated over time *in planta* and only 15 were differentially regulated between -P and +P conditions (Supplementary Data 5 and Supplementary Fig. 21). Among the top 100 most highly induced transporter genes *in planta* (average fold-change across all *in planta* conditions), those belonging to the Major Facilitator Superfamily (MFS) predominate (62/100), followed by those belonging to the Amino Acid-Polyamine-Organocation (APC) Superfamily (5/100), ATP-binding Cassette (ABC) Superfamily (4/100), Voltage-gated Ion Channel (VIC) Superfamily (3/100), P-type ATPase (P-ATPase) Superfamily (3/100) and Proposed Fatty Acid Group Translocation (FAT) Family (3/100). The massive *in planta* induction of MFS transporter genes and particularly those encoding sugar transporters among this set (> 25%) suggests that *C. tofieldiae* sugar uptake is dramatically enhanced *in planta*. This is consistent with the fact that the MS medium used in this study is not supplemented with any carbon source. Therefore the fungus relies entirely on the host plant for its survival and activates dedicated transporters to maximize sugar uptake. Notably, among the very few fungal transporter genes that are significantly up-regulated between -P and +P conditions, the gene that shows the strongest induction (log2FC = 3.5) encodes a phosphate:H+ symporter (CT04_05366) (Supplementary Data 5).

Secondary metabolism key genes (SMKGs): Similar to CSEP genes, only 15/71 SMKGs are actually expressed *in planta*, 11 are differentially regulated between at least one *in planta* sample and the *in vitro* condition, 4 are differentially regulated over time and none are differentially regulated between -P and +P conditions (Supplementary Data 5 and Supplementary Figs 21 and 22). The SMKGs showing the strongest *in planta* activation encode three polyketide synthases (CT04_04070, CT04_05283, CT04_08296) and a terpene synthase (CT04_04263) (Supplementary Data 5). This result indicates that, although *C. tofieldiae* is well equipped to produce a large array of potentially biologically active secondary metabolites, only a very restricted number of such molecules seems to be released during colonization of *Arabidopsis* roots by *C. tofieldiae*, consistent with its beneficial lifestyle.

4- *Comparative analysis of C. tofieldiae and C. incanum transcriptomes*

To compare the transcriptomes of beneficial *C. tofieldiae* and pathogenic *C. incanum* during colonization of *Arabidopsis* roots, we first compared the expression levels of 6,804 *C. incanum* and *C. tofieldiae* orthologous gene pairs at 10 and 24 dpi (Supplementary Data 6). After adjusting for library size and gene length, we identified 621 up- and 842 down-regulated genes between *C. tofieldiae* and *C. incanum* at 10 dpi. To get an impression of the functional relevance of the differentially expressed genes, GO term enrichment analysis was performed using the Blast2GO[71] java tool (Fisher's exact test; default settings) on the respective Blast2GO annotated *C. incanum* proteins in the orthologous gene pairs. This GO term enrichment analysis revealed that the *in planta* expressed genes up-regulated in *C. incanum* (vs. *C. tofieldiae*) at 10 dpi are significantly enriched for those encoding proteins involved in oxidoreduction processes (FDR < 0.05) (Supplementary Table 10a). In contrast, GO term enrichment analysis indicated that *C. tofieldiae* up-regulated genes (vs. *C. incanum*) at 24 dpi are significantly enriched for those encoding proteins involved in 'melanin metabolic process', 'phenol-containing compound biosynthetic process' and 'melanin biosynthetic process' (FDR < 0.05) (Supplementary Table 10a). This result is consistent with the formation of melanized microsclerotia in *C. tofieldiae* but not in *C. incanum*[1]. These microsclerotia are important resting structures for fungal long-term survival[72]. Similar GO term enrichment analysis among *Ct*- and *Ci*-specific gene sets that are expressed *in planta* indicated that terms related to 'nucleic acid phosphodiester bond hydrolysis' and 'RNA-dependent DNA replication' are significantly enriched among *Ci*-specific genes expressed *in planta* whereas terms associated with 'translation', 'amide/peptide biosynthetic/metabolic process' or 'respiratory electron transport chain' are significantly enriched among *Ct*-specific genes expressed *in planta* (Supplementary Table 10b).

By investigating the orthologous gene pairs showing the strongest differential expression between *C. tofieldiae* and *C. incanum* at 10 dpi, we observed that the expression of certain secondary metabolite-related genes is controlled in opposite directions in these two closely related species (Supplementary Data 6). For example, four genes encoding a TOXD related protein (CI_03439), an allergen-related protein (CI_02952), a berberine bridge enzyme (CI_10864) or an isoflavone reductase (CI_10399) were among the most highly induced genes in *C. incanum* while showing a much weaker expression in *C. tofieldiae* at 10 dpi (log2FC<-8). The top most differentially regulated orthologous gene pairs in *Ct* compared to *Ci* at 10 dpi (log2FC>7) encode a putative NmrA family transcriptional regulator protein (CT04_09092), a TATA-box-binding protein (CT04_06224), a vegetative cell wall protein gp1 (CT04_04287) and hypothetical proteins. Interestingly, one gene encoding a secreted protein with five predicted LysM domains ranked also among the top 10 differentially regulated genes between *C. tofieldiae* and *C. incanum* at 24 dpi (log2FC=6.4). This could indicate a preferential role for fungal chitin sequestration during root colonization by beneficial *C. tofieldiae*. Overall, >10% of *C. tofieldiae* and *C. incanum* orthologous genes are controlled in opposite directions during the early colonization stage, implying that *C. tofieldiae* and *C. incanum* differentially regulate distinct sets of genes during host infection.

To unravel the transcriptional footprints associated with evolution from a pathogenic to a beneficial lifestyle in *Colletotrichum* fungi, we also examined the expression profiles of *C. tofieldiae* and *C. incanum* genes (*in vitro* and *in planta* at 10 and 24 dpi) in functional categories often associated with fungal pathogenicity (i.e. CSEPs, CAZymes and SMKGs).

Analysis of CSEP gene expression profiles revealed that many more CSEP genes were expressed during root colonization by *C. incanum* (55) than by *C. tofieldiae* (18) (Fig. 3c, d). This three-fold difference cannot be solely explained by the lower read coverage in *C. tofieldiae* since the total number of *in planta* expressed genes is 6,693 for *C. tofieldiae* and 8,613 in *C. incanum* (1.3 fold difference). Indeed, by looking at the most highly expressed genes *in planta*, we observed seven times less *C. tofieldiae* CSEP genes among the top 1,000 expressed in *C. tofieldiae* than in *C. incanum*, confirming that *CSEP* expression is indeed dampened in *C. tofieldiae*-colonized roots. Interestingly, reduced CSEP gene activation was also reported for two other mutualistic fungi, including the ectomycorrhizal fungus *T. melanosporum*[47,73] and the mutualistic endophyte *P. indica,* for which only 20% of the effector candidate genes were identified as induced during *Arabidopsis* colonization[74]. This could indicate that loss and/or reduced transcript activation of candidate effector genes is a conserved adaptation to the mutualistic lifestyle in phylogenetically unrelated fungi.

By looking at the 398 *C. tofieldiae* and 532 *C. incanum* CAZyme-encoding genes that were expressed *in planta*, we also observed major differences in the respective expression profiles during root colonization (Fig. 4b, c). The CAZyme genes expression profile in *C. tofieldiae* is marked by the sequential activation of PCWDE genes in two successive waves, whereas for *C. incanum*, a stronger, earlier (10 dpi) and more uniform *in planta* activation was observed (Fig. 4b, c). Consistent with this, slightly more CAZyme genes were significantly up-regulated between 10 dpi and *in vitro* samples in *C. incanum* (313/532) than in *C. tofieldiae* (217/398) (Supplementary Data 5). Moreover, inspection of *C. tofieldiae* and *C. incanum* orthologous gene pairs encoding PCWDEs revealed that over twice as many were expressed higher in *C. incanum* than in *C. tofieldiae* during root colonization at 10 dpi (37 vs. 18) (Supplementary Data 6). In particular, transcripts encoding pectin-degrading enzymes showed the most striking induction, with 16 orthologous genes (two CE8, one CE8-PL1, four GH28, three PL1, two PL3, two PL4, two PL9) significantly induced in *C. incanum* vs. *C. tofieldiae* at 10 dpi and only 3 (one CE8, two PL1) significantly induced in *C. tofieldiae* vs. *C. incanum* (Supplementary Data 6). Using fluorescein diacetate as a marker for living plant cells, we could show that the majority of host cells in *Ct*-colonized roots are still alive at 10 dpi, whereas in *Ci*-colonized roots, most cells are not functional (Supplementary Fig. 1e). In addition, using Calcofluor staining we detected a massive depletion of cellulose from the cell walls of roots infected by *Ci* (10 dpi), likely due to the early and broad secretion of PCWDE by *Ci* during colonization of *Arabidopsis* roots (Fig. 4b, c and Supplementary Fig. 1f). In contrast, this removal of cellulose was not apparent in Ct-colonized roots (Supplementary Fig. 1f). Taken together, these results strongly suggest that Ci is a true pathogen on Arabidopsis. These results indicate that plant cell wall degradation, and particularly pectin and cellulose dissolution, occurs earlier during root colonization by pathogenic *C. incanum* while the later activation in *C. tofieldiae* might favor the beneficial relationship.

A relatively small, but similar number of SMKGs are expressed *in planta* in both *C. tofieldiae* (15/71) and *C. incanum* (19/70). For *C. incanum*, 14/19 (74%) and 12/19 (63%) were significantly up-regulated *in planta* at 10 dpi and 24 dpi (-P conditions), respectively, compared with the *in vitro* samples, whereas for *C. tofieldiae* only 4/15 (27%) and 6/15 (40%) are significantly up-regulated *in planta* at 10 dpi and 24 dpi, respectively, compared to *in vitro* hyphae (Supplementary 5 and 7). Six genes encoding PKS, four encoding NRPS, two encoding DMATS, one encoding PKS-NRPS and one encoding TS are significantly up-regulated at 6 dpi in *C. incanum* while only four PKS and one TS genes were significantly induced in *C. tofieldiae*. Although secretion of potentially bioactive and/or toxic compounds appears to be limited in both species, *C. tofieldiae* seems to further restrict their production through transcriptional regulation. The relative absence of activation of such genes in *C. tofieldiae* contrasts with

the massive induction of SMKG observed for *C. higginsianum* during penetration of *Arabidopsis* leaves[14]. These key enzymes, which potentially catalyze the biosynthesis of fungal toxins, generally are lacking in the genomes of obligate biotrophic and mutualistic fungi but are overrepresented in necrotrophic and saprotrophic fungi[75-77]. Although there is a large repertoire of these genes in the genome of beneficial *C. tofieldiae*, we hypothesize that their inactivation through transcriptional regulation is an essential adaptation for a life inside living host cells.

II- Analysis of the ***Arabidopsis*** root transcriptome

1- *Arabidopsis genes differentially regulated in response to C. tofieldiae colonization and phosphate starvation*

To identify genes that were differentially expressed in *A. thaliana* in the presence or absence of *C. tofieldiae* isolate 0861, we first compared for each phosphate condition the expression at each time point between the *C. tofieldiae*-infected and mock-treated samples (Supplementary Data 8). Depending on the selected time-point, 709 to 738 up-regulated genes and 343 to 597 down-regulated genes ($FDR<0.05$, $|log2FC|\geq 1$) were identified between *C. tofieldiae*-colonized and mock-treated roots (-P conditions). A similar signature was found under +P conditions between *C. tofieldiae*-colonized and mock-treated samples, but fewer genes (175 in total) were down-regulated at 24 dpi in this case (Supplementary Fig. 19). Overall, this indicates that *A. thaliana*, reacts to *C. tofieldiae* at each time point in both +P and -P conditions by differentially regulating ~5% of the expressed gene repertoire (22,087 in total, Supplementary Data 8). To identify differentially expressed genes between the two phosphate conditions, we then compared for each treatment (*C. tofieldiae*-colonized or mock-treated) the expression at each time point between -P and +P samples. The mock treated plants react strongly to phosphate starvation as early as 6 dpi (456 down-regulated genes, 337 up-regulated genes), then the transcriptional response to phosphate starvation decreases at 10 dpi (28 down-regulated genes, 44 up-regulated genes), increases again at 16 dpi and culminates at 24 dpi where 1,118 down-regulated and 1,096 up-regulated genes were identified (Supplementary Fig. 19). As previously reported, this indicates that phosphate starvation triggers major regulatory changes in the growth and development of *Arabidopsis* roots including inhibition of primary root growth, increase in lateral root formation and growth and production of root hairs[78-80]. The massive transcriptional reprogramming observed at 24 dpi between -P and +P conditions is also consistent with the morphology of the plants, which showed a marked growth retardation compared to those grown in +P conditions[1,81]. *C. tofieldiae* colonized roots also react to phosphate starvation but the number of regulated genes is slightly reduced compared to

mock-treated plants (Supplementary Fig. 19). The difference is particularly visible at 16 dpi where twice as many genes were identified as significantly regulated in -P vs. +P conditions in mock-treated plants compared to *C. tofieldiae*-colonized roots. This result suggests that *C. tofieldiae* might reduce phosphate stress and associated phosphate starvation responses under -P conditions. This is reminiscent of mycorrhizal fungi that are well known to reduce the impact of Pi depletion in the rhizosphere, thereby improving phosphate uptake and plant growth[82]. Moreover, this result is also consistent with P^{33} translocation experiments during the *C. tofieldiae*-*Arabidopsis* interaction, showing that orthophosphate is actively transferred to *Arabidopsis* leaves at 24 dpi under -P but not +P conditions and that the growth promoting activity is displayed only under -P conditions[1].

2- *Transcriptional reprogramming of Pi-starved and non-starved Arabidopsis roots in response to C. tofieldiae*

Based on the pairwise comparisons described above (16 in total), we identified 5,661 genes that were significantly regulated (FDR<0.05, |log2FC|≥1) in at least one of the tested comparisons (Supplementary Fig 19 and Supplementary Data 8). In order to extract the major transcriptional responses associated with each treatment over time (-P_Mock, +P_Mock, -P_Ct, +P_Ct), we generated heatmaps of gene expression profiles (see Supplementary Methods) and grouped the genes according to their expression profiles using k-means partitioning (k=20, Fig. 5a). We identified twenty major gene expression clusters among this gene set having non-redundant expression profiles (Fig. 5a). Ten clusters, showing a clear induction pattern associated with phosphate starvation response (Cluster 2 and 4), fungal colonization response (induction: Cluster 1, Cluster 3, Cluster 7, Cluster 10; repression: Cluster 5, Cluster 6) or more specific responses related to both fungal colonization and phosphate sufficient (Cluster 9) or phosphate deficient (Cluster 8) conditions were selected for further Gene Ontology (GO) term enrichment analysis (Fig. 5b and Supplementary Data 9).

In Fig. 5b, each GO term is depicted as a circle and the contribution (%) of each individual cluster to the overall GO term enrichment is represented by different colors. Notably, the most significantly enriched GO terms (hypergeometric test, Bonferroni step down correction) in clusters 2 and 4 are related to 'lipid metabolic processes', 'response to starvation' and 'homeostasis processes' (Fig. 5b and Supplementary Data 9). Lipid remodeling is one of the most striking metabolic responses observed during phosphate starvation[83], which validates that the phosphate conditions used in our study (50μM) were sufficient to provoke phosphate starvation. This response consists of a replacement of membrane phospholipids by glycolipids followed by the degradation of the phospholipids to liberate Pi in the cells[84].

GO terms related to root cell differentiation such as ‘trichoblast differentiation’, ‘epidermal cell differentiation’, ‘root morphogenesis’ or ‘root hair elongation’ are significantly enriched in the cluster 8 (Fig. 5b and Supplementary Data 9). The corresponding transcripts accumulate preferentially in *C. tofieldiae*-colonized roots (earlier and stronger induction) compared to mock-treated plants under low phosphate conditions (see cluster 8, Fig. 5a). This result indicates that the presence of *C. tofieldiae* and to a lesser extent phosphate starvation, induce a major transcriptional reprogramming to control root architecture. Particularly, numerous genes encoding root hair specific proteins are represented in this cluster and many are significantly up-regulated (*RHS8*, *RHS12*, *RHS13*, *RHS15*, *RHS19*) in *Ct*-colonized vs. mock treated roots (Fig. 5a and Supplementary Data 8). This result strongly supports the hypothesis that *C. tofieldiae* enhances root hair formation in *Arabidopsis* during a phosphate stress. Importantly, it was previously reported that root hair length has a major impact on phosphate uptake in *Arabidopsis*, as accessions with long and dense root hairs were found to be more efficient in phosphate acquisition[85]. Furthermore, it has been shown that the ectomycorrhizal fungi *L. bicolor* and *T. melanosporum* promote not only lateral root development but also root hair growth in *Arabidopsis* through the release of volatile compounds and/or auxin[86-88]. This fungus-dependent remodeling of root architecture might play a key role to enhance phosphate uptake by the plant. Root colonization by *C. tofieldiae* also induces early plant responses, particularly the expression of genes associated with ion transport and metabolism, which appears to be independent of the nutrient status of the plant (Cluster 3, Fig. 5b). In particular, GO terms associated with ‘iron ion transport’, ‘nitrate transport’, ‘metal ion transport’ or ‘peptide transport’ are markedly enriched in this cluster (Fig. 5b and Supplementary Data 9). This observation suggests that *C. tofieldiae* could influence ion homeostasis in *Arabidopsis* roots during the first weeks of the interaction in both phosphate-sufficient and phosphate-deficient conditions.

Dozens of GO terms associated with defense responses including the terms ‘indole glucosinolate metabolic process’, ‘response to chitin’, ‘response to mechanical stimulus’, ‘ethylene metabolic process’ and ‘defense response by callose deposition’ are massively enriched in *Ct*-colonized roots but specifically under +P conditions (Cluster 9, Fig. 5b and Supplementary Data 9). Importantly, 33% of the genes belonging to this cluster were indeed significantly up-regulated in +P vs. -P conditions in *Ct*-colonized roots at 24 dpi (log2FC≥1, FDR<0.05) (Supplementary Data 8). Consistent with the hypothesis that the activation of defense responses depends on the phosphate status of the plants, 8% of the genes significantly induced in +P vs. -P conditions at that stage encode chitin-responsive proteins compared to less than 1% of the repressed genes. Further inspection of all expressed genes annotated as ‘chitin responsive’ genes (411 in total) (Gene Ontology Annotation, https://www.arabidopsis.org/)

showed a similar pattern, marked by a strong transcript accumulation at 24 dpi under +P conditions only (Supplementary Fig. 23). Accordingly, GO term enrichment analysis among significantly up-regulated *Arabidopsis* genes also indicated a striking enrichment of GO terms associated with 'defense responses', 'response to hormone' and 'response to stimulus' among the genes induced in *C. tofieldiae*-colonized roots under +P conditions compared to -P conditions (Supplementary Fig. 24c). In contrast GO terms associated with 'photosynthesis', 'growth and development' and 'transport' were significantly enriched among the genes significantly up-regulated in *C. tofieldiae*-colonized roots under -P conditions compared to +P conditions (Supplementary Fig. 24c). Taken together, these results strongly support the idea that *C. tofieldiae*-colonized roots react differently to the fungus depending on the nutritional status of the plant. Whereas beneficial responses are prioritized under -P conditions, defense responses are activated under +P conditions. This remarkable difference in transcriptional reprogramming between starved and non-starved roots colonized by *C. tofieldiae* may be explained by the sessile nature of plants, which must balance their resource allocation strategically to maximize growth and survival. Combinations of stresses, including cold, high light, salt, heat, and bacterial flagellin have been tested on *Arabidopsis*, revealing that 61% of the transcriptome changes in response to double stresses were not predicted from the responses to single stress treatments[89]. This indicates that plants have evolved complex mechanisms to cope with combinations of stresses in order to maximize their survival. Consistent with our results, it has been shown that high phosphate application can impact arbuscule development and markedly decrease AM fungal biomass per plant[90]. Furthermore, it has been reported that reduced colonization of *Cucumis sativus* (cucumber) in high phosphate conditions resulted from slower growth of fungal infection units within roots as well as reduced penetration efficiency[91]. Under high phosphate conditions, *Medicago truncatula* roots are still able to respond to fungal signals based on calcium spiking profiles, suggesting that the plant roots are not blind to AM fungi[92]. The reasons underlying the reduced root colonization by AM fungi under high phosphate conditions have remained elusive. Here, we identified a clear trade-off between the phosphate nutritional status and host immunity, suggesting that there is a sensory node relaying information between the phosphate starvation response and the immune system. Our results also suggest a remarkable capacity of *Arabidopsis* roots to prioritize transcriptional response in order to favor defense responses under phosphate-sufficient conditions and beneficial responses (root cell differentiation, transport) under phosphate-deficient conditions. This is consistent with the fact that *C. tofieldiae* promotes plant growth only under phosphate deficient conditions, and that radiolabelled phosphate is specifically detected in the leaves of *C. tofieldiae*-colonized plants under low phosphate conditions[1]. Furthermore, the presence of *C. tofieldiae* significantly induces the expression of two plant phosphate transporter genes (*Pht1;2*, *Pht1;3*) in Pi-starved roots compared to mock-control plants[1]. The transcriptional reprogramming

observed in *C. tofieldiae*-colonized roots under phosphate-deficient conditions appears very similar to the genetic program deployed by plant roots during colonization by mycorrhizal fungi[87,93-95]. However, *Ct*-mediated growth promotion was not altered when knockout mutants of the pht1 transporter family were used (*pht1;1*, *pht1;2*, *pht1;9*, *pht1;1pht1;4)*, likely due to functional redundancy within this transporter family[1]. Nonetheless, *Ct*-mediated plant growth promotion (-P conditions) was significantly impaired in the *phf1* mutant, involved in Pi uptake by facilitating the trafficking of phosphate transporters from the endoplasmic reticulum/Golgi to the plasma membrane[96] and in the phl1phr1 double mutants that lack transcriptional regulators of the phosphate starvation response[97]. Overall, our results indicate that the phosphate starvation responses and defense responses are tightly connected and regulated in *Arabidopsis* roots to control fungal invasion and maintain the beneficial association. Consistent with this apparent link between phosphate starvation and immune responses, a phytoplasma effector was recently shown to alter both responses in *Arabidopsis*[98].

3- ***Key regulatory nodes mediating phosphate-status-dependent transcriptional reprogramming in C. tofieldiae-colonized roots***

To narrow down the number of candidate genes and identify those that are likely mediating the different transcriptional responses represented by the clusters 8 and 9 (Fig 5a, b)), we selected all the genes in each cluster and examined how many of these genes were also often found to be co-expressed in other *Arabidopsis* expression datasets. We used the ATTED-II gene co-expression database (http://atted.jp/) to identify the genes that are often co-regulated within each cluster and the corresponding co-expression networks for each cluster were visualized using Cytoscape (Fig. 5c). In these networks the genes are represented as colored nodes that are connected by edges if they are co-regulated. For each cluster the most highly connected hub genes (> 5 connections) are highlighted in black. Among the hub genes detected in cluster 8, many encode proteins involved in root hair development (RHSs, SHV2, PIP5K3, LRX1, IRE) and cell wall remodeling (XTR9, AGP3, EXT10, FLA6, EXPA18, ADF8, ADF11, EXPA7, XTH26, XTH13, EXT12), indicating that extensive transcriptional reprogramming occurs in roots under phosphate-deficient conditions (Fig. 5c). Importantly, these responses were enhanced in *C. tofieldiae*-colonized roots (see above), indicating that the beneficial fungus further enhances root hair development in *Arabidopsis*. Finally, twenty seven hub genes were identified in cluster 9 and these encode well-characterized defense-related proteins such as WRKY transcription factors (WRKY33, WRKY40, WRKY46), calcium binding proteins (CML38, CML 39, EDA39), Ethylene-responsive factors (ERF11, ERF13), the jasmonic acid biosynthesis regulator ORA47, the elicitor peptide precursor PROPEP3, indolic glucosinolate pathway transcription factors (MYB51, CYP81F2), but also more

general stress-related genes such as *CAF1a*, *ZAT10*, *CYP707A3*, *MYB15*, *SZF1* or *DIC2* (Fig. 5c and Supplementary Data 8). Notably, five genes involved in the indole glucosinolate biosynthesis pathway were detected in cluster 9, suggesting that glucosinolate-derived molecules are particularly important for the phosphate-dependent control of fungal colonization (see below).

The molecular mechanism through which fungi activate the plant immune system, as well as the pivotal role played by plant hormones, have been extensively described for leaf-infecting pathogens[52,99]. In leaves, the phytohormones salicylic acid (SA), jasmonic acid (JA), and ethylene (ET) are well-characterized components of MAMP triggered-immunity. In general, the activation of the SA pathway inhibits pathogens with a biotrophic lifestyle, whereas JA- and ET-dependent signaling inhibits pathogens with a necrotrophic lifestyle[100,101]. However, there is very little information about how the root immune system reacts and responds to infection by pathogenic and/or beneficial fungi. Using different microbe-associated molecular patterns (MAMPs), it has been shown that *Arabidopsis* roots leverage a strong tissue-specific response to three MAMPs including the flagellar peptide flg22, peptidoglycan, and chitin. These responses were dependent on ET-signaling, the 4-methoxy-indole-3-ylmethylglucosinolate biosynthetic pathway, and the PEN2 myrosinase, but not SA or JA signaling[102]. Our results point to the same transcriptional activation of predominantly ethylene- and PEN2-mediated defense responses in *Ct*-colonized roots under phosphate-sufficient conditions. Elsewhere we report that depletion of all tryptophan-derived metabolites (*cyp79B2/B3* double mutants) not only abolishes the beneficial interaction between *Arabidopsis* and *Ct* (-P), but also allows excessive *Ct* colonization, which ultimately kills host roots[1]. Similar results were also reported during root colonization by *P. indica*, indicating that *Arabidopsis* requires a non-compromized immune system to maintain the beneficial interaction with phylogenetically unrelated root endophytes[74]. A more systematic inspection of *A. thaliana* mutants impaired in the production of particular classes of tryptophan-derived compounds revealed the following key findings[1]. Firstly, the plant growth-promoting activity is abolished in *Arabidopsis* mutants impaired in indole-derived glucosinolate biosynthesis including *pen2* single, *pen2pad3* double and *myb34myb51myb122* triple mutants[103]. Secondly, the plant growth promoting activity was not reduced in *Arabidopsis cyp71A12* or *cyp71A13* mutants, which lack sequence-related P450 monooxygenases required for camalexin biosynthesis in roots[104]. Overall, these results indicate that tryptophan-derived compounds are critical for controlling fungal overgrowth *in planta* and that indole-derived glucosinolates, but not camalexin, are required for maintaining the beneficial interaction. Importantly, glucosinolate biosynthesis and the accumulation of the corresponding metabolites, has been recently shown to be controlled by the transcription factor PHR1 (PHOSPHATE STARVATION RESPONSE 1), providing a clear connection between the phosphate starvation response and synthesis of glucosinolate-derived

metabolites in *Arabidopsis* roots[105]. Taken together, these data indicate a functional link between innate immunity and the phosphate starvation response during beneficial interactions with *Ct*. Similar to our findings, it has been reported that ET signaling is also triggered in *Arabidopsis* roots during colonization by *P. indica* and is an important component of the mutualistic association[106]. Moreover, it was shown recently that the ectomycorrhizal fungus *L. bicolor* triggers ET and JA defense responses at late stages of root colonization, possibly to control fungal growth within the root tissues[107]. Our results indicate that ET-mediated defense responses are particularly enhanced at 24 dpi in *C. tofieldiae*-colonized roots grown under phosphate-sufficient conditions. In particular, several ET-responsive factors were identified among the 411 analyzed chitin-responsive genes induced at that stage compared to the phosphate-deficient condition. These genes include *ERF1*, *ERF2*, *ERF6*, *ERF11* and the 1-aminocyclopropane-l-carboxylic acid (ACC) synthase 6, involved in Ethylene biosynthesis (Supplementary Fig. 23a, b). Analysis of the connectivity between presumptive chitin-responsive genes that were significantly induced at 24 dpi in +P vs. -P conditions (Supplementary Fig. 23b) identified four regulatory hub genes encoding well-known transcription factors involved in defense responses against pathogens (WRKY40 and WRKY33[108]) or stress responses (SZF1) as well as the mitochondrial dicarboxylate carrier DIC2 (Supplementary Fig. 23b). Importantly, DIC2 can transport Pi across the inner mitochondrial membrane[109] and is regulated during phosphate starvation[80]. Our results suggest that DIC2 could be an important component of the phosphate-status-dependent activation of the immune response since it links the phosphate nutritional status to the plant immune system.

4- *Transcriptome analysis of Pi-starved Arabidopsis roots in response to beneficial C. tofieldiae and pathogenic C. incanum.*

In order to discriminate whether the reduced activation of plant defense responses observed during *C. tofieldiae* colonization under -P conditions reflects a defect in defense activation due to the phosphate deficiency or a more complex mechanism specifically triggered in the presence of beneficial *C. tofieldiae*, we compared the transcriptome of Pi-starved *Arabidopsis* roots (50µM) in response to either *C. incanum* or *C. tofieldiae* at 10 dpi (Supplementary Data 10). To identify differentially expressed genes, we compared the normalized expression levels between three different treatments, (namely '*C. tofieldiae*-colonized roots vs. mock-treated', '*C. incanum*-colonized roots vs. mock-treated' and '*C. incanum*-colonized roots vs *C. tofieldiae*-colonized roots'). In a first RNA-seq experiment performed for *C. incanum,* the alignment efficiency was very low (E1, see Supplementary Table 8), thus we could not rely on these data for analysis of the fungal transcriptome, but we could still make use of the data in our analysis of the plant transcriptome. Therefore, for the *C. incanum*-colonized roots, we included the data

obtained from two fully independent experiments in our analysis, representing 6 replicates in total (E1 and E2). In order to stringently identify differentially expressed genes between *C. incanum*-infected and *C. tofieldiae*-infected plants, we separately included the samples from the two *C. incanum*-treated batches in our analysis and only accepted a gene as significantly different to the *Ct*-infected samples if the expression difference was significant and consistent across both batches ($|\log2FC| \geq 1$, FDR<0.05). With this stringent procedure, we identified a total of 2,009 genes that were differentially expressed in *C. tofieldiae*-colonized roots compared to *C. incanum*-colonized roots (Fig. 6a and Supplementary Data 10). Remarkably, GO term enrichment analysis revealed that the genes with significantly higher expression in *C. tofieldiae*-colonized roots at 10 dpi were enriched for GO terms related to cellular response to iron ion starvation, ion/nitrate transport, cell maturation, root hair cell differentiation and root morphogenesis while the genes with significantly higher expression in *C. incanum*-colonized roots at 10 dpi were enriched for GO terms related to response to stimulus, response to stress, defense responses, sulfur metabolic processes, response to chitin and glycoside metabolic processes (Fig. 6b). Taken together, these results indicate that *Arabidopsis* remains able to activate defense responses under -P conditions. Our data also demonstrate that *Arabidopsis* activates contrasting transcriptional programs in response to these closely-related fungal species. Whereas massive defense responses are triggered in *C. incanum*-colonized roots under phosphate-deficient conditions at 10 dpi, beneficial responses dominate in *C. tofieldiae*-colonized roots at the same time point, including modulation of root architecture and enhanced ion transport activity. Consistent with this, genes encoding the plant phosphate transporters Pht1;4, Pht1;5, Pht2;1, Pht3;2 and Pht4;1 are significantly up-regulated in *C. tofieldiae*-colonized roots compared with *C. incanum*-colonized roots (Supplementary Data 10).

Supplementary Note 10

Validation of RNAseq data using RT-qPCR

To validate our RNA-Seq data, expression levels of 10 *Arabidopsis* genes were assessed using Reverse Transcription quantitative Polymerase Chain Reaction (RT-qPCR) (See Supplementary Methods). These genes encode the phosphate transporters Pht1;3 and Pht1;4 (AT5G43360, cluster 1 and AT2G38940, cluster 2), the FLG22-induced receptor-like kinase 1 (AT2G19190, cluster 9), the sulfoquinovosyldiacylglycerol 2 (AT5G01220, cluster 2) the cyclin protein P4;2 (AT5G61650, cluster 8), the root hair specific proteins 13 and 19 (AT4G02270 and AT5G67400, cluster 8), the WRKY DNA-binding proteins 33 and 40 (AT2G38470, AT1G80840, cluster 9) and the ethylene-responsive element binding factor 13 (AT2G44840, cluster 9). Gene expression levels were normalized using the reference gene actin (*ACT2*, AT3G18780). The primer pairs used in this study are presented in Supplementary Table 9.

Similarly, 10 *C. tofieldiae* genes were also selected for RT-qPCR analysis in order to validate fungal gene expression measured by RNAseq. The selected genes encode: a phosphate:H+ symporter (CT04_05366), three CSEPs (CT04_00229, CT04_09572, CT04_07148), a thiamine biosynthesis protein (CT04_02539), a cellulose 1,4-beta-cellobiosidase (CT04_06091), a pectate lyase (CT04_07285), a polyketide synthase (CT04_04070), a LysM domain-containing protein (CT04_01769) and a hypothetical protein (CT04_02713). Gene expression levels were normalized using the reference gene tubulin beta-1 chain (CT04_12898) for which stable expression levels were detected across all conditions based on our RNAseq data. The primer pairs used in this study are presented in Supplementary Table 9.

Similar to RNAseq data, Relative Expression Indexes (REI) were calculated. The overall expression profile and dynamics of gene activation were highly similar between the two methods for both *C. tofieldiae* (Supplementary Fig. 18a) and *Arabidopsis* genes (Fig. 5d and Supplementary Fig. 18b). Individual datapoints were then plotted to assess the correlation between gene expression profiles obtained by RNAseq and RT-qPCR. For both *C. tofieldiae* and *Arabidopsis* genes (Supplementary Fig. 18d and c, respectively), high R^2 values were observed for the fitted linear regression models (R^2 = 0.876 for *C. tofieldiae* and R^2 = 0.802 for *Arabidopsis*) and the slopes of the regression lines were close to unity (y = 0.91 for *C. tofieldiae* and y = 0.87 for *Arabidopsis*), indicating minimal variation in the expression data and very high consistency between the two techniques (Supplementary Fig. 18). This validation (Supplementary Fig. 18 and Fig. 5d) also confirms the expression pattern of some marker genes belonging to cluster 1, cluster 2, cluster 8 and cluster 9 defined using RNAseq (Fig. 5a).

Supplementary Note 11

Phylogeny and divergence date estimation

Phylogenetic analyses:

1. Phylogenetic tree based on whole genome sequencing: We retrieved sequences encoding all single-copy genes that are shared among *C. tofieldiae*, *C. incanum* and four previously sequenced species, namely *C. higginsianum*, *C. graminicola*, *C. fructicola* (previously reported as *C. gloeosporioides*[15]) and *C. orbiculare*. We then generated a concatenated alignment of these sequences (4,984 gene families in total) and used MrBayes to infer the tree, with the General Time Reversible, Invariant sites plus Gamma (GTR+I+G) model (See Supplementary Fig. 2a).

2. Phylogenetic tree based on six loci: Genomic DNA of isolates CBS 127615, CBS 130834, CBS 130835 and CBS 130851 was extracted using the method of Damm *et al.*[110]. The 5.8S nuclear ribosomal gene with the two flanking internal transcribed spacers (ITS), partial sequences of the actin (ACT), chitin synthase 1 (CHS-1), beta-tubulin (TUB2) and histone3 (HIS3) genes as well as a 200-bp intron of the glyceraldehyde-3-phosphate dehydrogenase gene (GAPDH) were amplified and sequenced using the primer pairs ITS-1F[111] + ITS-4[112], ACT-512F + ACT-783R[113], CHS-354R + CHS-79F[113], BT2Fd + BT4R[114], CYLH3F + CYLH3R[115] and GDF1 + GDR1[116], respectively (Supplementary Fig. 2c). The PCRs were performed as described by Damm *et al.*[18] and Woudenberg *et al.*[114]. The DNA sequences obtained from forward and reverse primers were assembled using Bionumerics v.4.60 (Applied Maths, St-Marthens-Lathem, Belgium). The respective sequences of strains MAFF 238704, MAFF 238706, MAFF 238712, MAFF 238713 were retrieved from NIAS Genbank (http://www.gene.affrc.go.jp), while those of strain *Ct*0861 were extracted from the genome assembly created in this study. Reference strains were selected on the basis of BLASTn searches against GenBank (http://www.ncbi.nlm.nih.gov/genbank) and multi-locus BLAST searches in Q-Bank (http://www.q-bank.eu/Fungi), which includes a dedicated *Colletotrichum* identification database, and were added to the outgroup (*C. dematium*, strain CBS 125.25). The alignment was created and manually adjusted using Sequence Alignment Editor v. 2.0a11[117]. A maximum parsimony analysis was performed on the multilocus alignment (ITS, TUB2, ACT, HIS3, CHS-1, GAPDH) with PAUP (Phylogenetic Analysis Using Parsimony) v.4.0b10[118] using the heuristic search option with 100 random sequence additions and tree bisection and reconstruction (TBR) as the branch-swapping algorithm (data not shown). Alignment gaps were treated as missing and all characters were unordered and of equal weight. The robustness of the trees was evaluated by 500 bootstrap replications with 10 random sequence additions[119]. Sequences derived in this study were submitted to GenBank. BLAST searches

against GenBank and Q-bank, as well as a preliminary phylogeny of all currently known *Colletotrichum* species with curved conidia from herbaceous hosts (not shown), indicated that the two species studied here belong to the *C. spaethianum* species complex[18,28]. In order to confirm the identity of the two species, a phylogeny of all currently known species in that species complex was constructed. In the multigene analyses of 28 isolates including the outgroup (Supplementary Fig. 2b, c), 2196 characters including the alignment gaps were processed, of which 260 characters were parsimony-informative, 166 parsimony-uninformative and 1770 constant. The heuristic search using PAUP resulted in 39 most parsimonious trees, one of which is shown in Supplementary Fig. 2b.

Species tree reconstruction and divergence date estimation. Single-copy gene families, identified by clustering the predicted protein sequences from *C. incanum*, the five *C. tofieldieae* isolates and sixteen additional fungal species, were used to estimate (a) the age of the *Colletotrichum* crown, (b) the age of the common ancestor of *C. tofieldiae* and *C. incanum* and (c) the age of the *C. tofieldiae* crown (Fig. 1a and Supplementary Table 3). The clustering was performed using the MCL program[120] with an inflation factor of 1.2, producing a total of 136 single-copy gene families. Each single-copy gene family was then aligned with MAFFT[121] and subjected to phylogenetic analysis with PhyML using the best substitution model determined by ProtTest[122]. PhyML was also used to compute SH-Like branch support values for each tree. To select the gene families to use for the multi-gene phylogeny, we followed the guidelines of Salichos and Rokas[123] and selected families with the strongest phylogenetic signal. The phylogenetic signal of each family was calculated by computing the average SH-like branch support value of all branches within each tree (Fig. 1a). We selected 20 families with the highest average support value to construct the multi-gene phylogeny. The clustering, family selection, and phylogenetic analyses were performed with scripts in the Mirlo package (https://github.com/mthon/mirlo). Aligned sequences from the 20 gene families with the strongest phylogenetic signals were concatenated and used to construct a phylogenetic tree with PhyML using the JTT substitution model. The alignment concatenation and phylogenetic analysis were performed with Geneious 8 (www.geneious.com). The phylogeny was calibrated using the penalized likelihood method implemented in r8s using one primary and two secondary calibration points[124]. Node 1 (Fig. 1a and Supplementary Table 3) was fixed to 400 Mya based on analysis of the *Paleopyrenomycites* fossil by Lucking *et al.*[9]. Node 2 was constrained to 339 - 207 Mya based on the phylogenetic analysis of Beimforde *et al.*[10] and Node 3 was constrained to 206 - 146 Mya based on the analysis of Sung *et al.*[11] (Fig. 1a and Supplementary Table 3). The species tree shown in Supplementary Figs. 9-12 was constructed using the same method, except that time calibration was not performed.

Supplementary Note 12

Annotation of genes encoding secondary metabolism key enzymes

For annotating the secondary metabolism key genes (SMKGs) in *Colletotrichum* species, we used an in-house bioinformatic pipeline that was developed as follows. Briefly, genes encoding each class of key secondary metabolism enzymes (DMATS, NRPS, PKS, PKS-NRPS, and TS) were identified from the genome of *Magnaporthe oryzae* (Broad version 5) by expert manual annotation (based on Collemare *et al.*[125]). These sequences were compared to the INTERPRO database[126] to identify domains specific for each key gene family, which were then aligned using MUSCLE v3.8.31[127]. Corresponding HMM profiles were generated using the HMMER 3.0 package[128] and the resulting database of HMM profiles was then used to scan the predicted proteomes of each *Colletotrichum* species to identify candidate secondary metabolism key enzymes having the required characteristic domains, or combinations of domains. To evaluate the accuracy of this pipeline, we compared the predictions for *Colletotrichum graminicola* to the manually annotated SMKGs of that species reported previously[14]. The numbers of SMKGs predicted were as follows (pipeline/manual): PKS (33/39), NRPS (6/7), hybrid PKS-NRPS (7/7), DMATS (6/7), TS (5/14). The differences result from the pipeline being more stringent than the manual annotation, because any SMKG missing even one of the mandatory domains was rejected.

Supplementary References

1. Hiruma, K. *et al*. Root endophyte *Colletotrichum tofieldiae* confers plant fitness benefits that are phosphate status-dependent. *Cell*. In press.
2. Ofir, C. & Pupko, T. Inference and characterization of horizontally transferred gene families using stochastic mapping. *Mol. Biol. Evol.* 27, 703–713 (2010).
3. Pinedo, C. *et al*. (2008) Sesquiterpene synthase from the botrydial biosynthetic gene cluster of the phytopathogen *Botrytis cinerea*. ACS Chemical Biology 3:791-801.
4. Tamura, K. *et al*. MEGA5: molecular evolutionary genetics analysis using maximum likelihood, evolutionary distance, and maximum parsimony methods. *Mol. Biol. Evol.* 28, 2731–279 (2011).
5. Supek, F., Bošnjak, M., Škunca, N. & Šmuc, T. REVIGO summarizes and visualizes long lists of gene ontology terms. *PLoS One* 6, e21800 (2011).
6. Sato, T. *et al*. Anthracnose of Japanese radish caused by *Colletotrichum dematium*. *J. Gen. Plant Pathol.* 71, 380–383 (2005).
7. Edgar, R. C. & Myers, E. W. PILER: identification and classification of genomic repeats. *Bioinformatics* 21, Supplement 1: i152–i158 (2005).
8. Jurka. J. *et al*. Repbase Update, a database of eukaryotic repetitive elements. Cytogenetic and Genome Research 110: 462–467 (2005).
9. Lucking, R., Huhndorf, S., Pfister, D. H., Plata, E. R. & Lumbsch, H. T. Fungi evolved right on track. *Mycologia* 101, 810–822 (2009).
10. Beimforde, C. *et al.* Estimating the Phanerozoic history of the Ascomycota lineages: Combining fossil and molecular data. *Mol. Phylogenet. Evol.* 78, 386–398 (2014).
11. Sung, G.-H., Poinar Jr., G. O. & Spatafora, J. W. The oldest fossil evidence of animal parasitism by fungi supports a Cretaceous diversification of fungal–arthropod symbioses. *Mol. Phylogenet. Evol.* 49, 495–502 (2008).
12. Olsen, S., Cole, C., Watanabe, F. & Dean, L. Estimation of available phosphorus in soils by extraction with sodium bicarbonate. USDA Circular Nr 939, US Gov. Print. Office, Washington, D.C. (1954).
13. García, E., Alonso, Á., Platas, G. & Sacristán, S. The endophytic mycobiota of *Arabidopsis thaliana*. *Fungal Divers.* 60, 71–89 (2013).
14. O'Connell, R. J. *et al*. Lifestyle transitions in plant pathogenic *Colletotrichum* fungi deciphered by genome and transcriptome analyses. *Nat. Genet.* 44, 1060–1065 (2012).
15. Gan, P. *et al*. Comparative genomic and transcriptomic analyses reveal the hemibiotrophic stage shift of *Colletotrichum* fungi. *New Phytol.* 197, 1236–1249 (2012).

16. Tavares, S. *et al*. Genome size analyses of Pucciniales reveal the largest fungal genomes. *Front Plant Sci.* 5:422 (2014).

17. Nakayashiki, H. *et al*. Pyret, a Ty3/Gypsy retrotransposon in *Magnaporthe grisea* contains an extra domain between the nucleocapsid and protease domains. *Nucleic Acids Res.* 29: 4106–4113 (2001).

18. Damm, U., Woudenberg, J. H. C., Cannon, P. F. & Crous, P. W. *Colletotrichum* species with curved conidia from herbaceous hosts. *Fungal Divers.* 39, 45-87 (2009).

19. Yang, H.-C., Haundenshield, J. S. & Hartman, G. L. *Colletotrichum incanum* sp. nov., a curved-conidial species causing soybean anthracnose in USA. *Mycologia* 106, 32-42 (2014).

20. Langergraber, K. E. *et al*. Generation times in wild chimpanzees and gorillas suggest earlier divergence times in great ape and human evolution. *Proc. Natl. Acad. Sci. USA* 109, 15716–15721 (2012).

21. Raffaele, S. *et al*. Genome evolution following host jumps in the Irish potato famine pathogen lineage. Science 330, 1540–1543 (2010).

22. Hacquard, S. *et al*. Mosaic genome structure of the barley powdery mildew pathogen and conservation of transcriptional programs in divergent hosts. *Proc Natl Acad Sci USA* 110, E2219–E2228 (2013).

23. Wicker, T. *et al*. The wheat powdery mildew genome shows the unique evolution of an obligate biotroph. *Nat. Genet.* 45, 1092–1096 (2013).

24. Stukenbrock, E.H., Christiansen, F.B., Hansen, T.T., Dutheil J.Y. & Schierup, M.H. Fusion of two divergent fungal individuals led to the recent emergence of a unique widespread pathogen species. *Proc. Natl. Acad. Sci. USA* 109, 10954–10959 (2012).

25. McMullan, M. et *al.* Evidence for suppression of immunity as a driver for genomic introgressions and host range expansion in races of *Albugo candida*, a generalist parasite. *eLife* 4, e04550 (2015).

26. Vaillancourt, L. J. & Hanau, R. M. A method for genetic analysis of *Glomerella graminicola* (*Colletotrichum graminicola*) from maize. *Phytopathol.* 81, 530–534 (1991).

27. Wheeler, H. E. Linkage groups in *Glomerella*. *Am. J. Bot.* 43, 1–6 (1956).

28. Cannon, P. F., Damm, U., Johnston, P. R. & Weir, B. S. Colletotrichum - current status and future directions. *Stud. Mycol.* 73, 181–213 (2012).

29. He, C., Rusu, A. G., Poplawski, A. M., Irwin, J. A. G. & Manners, J. M. Transfer of a supernumerary chromosome between vegetatively incompatible biotypes of the fungus *Colletotrichum gloeosporioides*. *Genetics* 150, 1459–1466 (1998).

30. da Silva Franco, C. C. *et al*. Vegetative compatibility groups and parasexual segregation in *Colletotrichum acutatum* isolates infecting different hosts. *Phytopathology* 101, 923–928 (2011).
31. Li, L., Stoeckert, C. J. & Roos, D. S. OrthoMCL: Identification of Ortholog Groups for Eukaryotic Genomes. *Genome Res.* 13, 2178–2189 (2013).
32. Fisher, R. A. On the interpretation of χ2 from contingency tables, and the calculation of P. *J. Roy. Stat. Soc.* 85, 87–94 (1922).
33. Benjamini, Y. & Hochberg, Y. Controlling the false discovery rate: a practical and powerful approach to multiple testing. *J. Roy. Stat. Soc. Ser. B* 57, 289–300 (1995).
34. Elias, I. & Tuller, T. Reconstruction of ancestral genomic sequences using likelihood. *J. Comput. Biol.* 14, 216–237 (2007).
35. Yang, Z. & Bielawski, J. P. Statistical methods for detecting molecular adaptation. *Trends Ecol. Evol.* 15, 496–503 (2000).
36. Pond, S. L. K. & Frost, S. D. W. Not So Different After All: A Comparison of Methods for Detecting Amino Acid Sites Under Selection. *Mol. Biol. Evol.* 22, 1208–1222 (2005).
37. Suzuki, Y. & Gojobori, T. A method for detecting positive selection at single amino acid sites. *Mol. Biol. Evol.* 16, 1315–1328 (1999).
38. Dyrka, W. *et al*. Diversity and variability of NOD-like receptors in fungi. *Genome Biol. Evol.* (2014).
39. Glass, N. L. & Kuldau, G. A. Mating Type and Vegetative Incompatibility in Filamentous Ascomycetes. *Annu. Rev. Phytopathol.* 30, 201–224 (1992).
40. Leslie, J. F. Fungal Vegetative Compatibility. *Annu. Rev. Phytopathol.* 31, 127–150 (1993).
41. Saupe, S. J. Molecular Genetics of Heterokaryon Incompatibility in Filamentous Ascomycetes. *Microbiol. Mol. Biol. Rev.* 64, 489–502 (2000).
42. Puhalla, J. E. Classification of strains of Fusarium oxysporum on the basis of vegetative compatibility. *Can. J. Bot.* 63, 179–183 (1985).
43. Paoletti, M. & Saupe, S. J., Fungal incompatibility: Evolutionary origin in pathogen defense? *BioEssays* 31, 1201–1210 (2009).
44. Martin, F. *et al*. The genome of *Laccaria bicolor* provides insights into mycorrhizal symbiosis. *Nature* 452, 88–92 (2008).
45. Zuccaro, A. *et al*. Endophytic life strategies decoded by genome and transcriptome analyses of the mutualistic root symbiont *Piriformospora indica*. *PLoS Pathog.* 7, e1002290 (2011).
46. Tisserant E. *et al.* Genome of an arbuscular mycorrhizal fungus provides insight into the oldest plant symbiosis. *Proc. Natl. Acad. Sci. USA* 110, 20117–20122 (2013).

47. Martin, F. *et al*. Périgord black truffle genome uncovers evolutionary origins and mechanisms of symbiosis. *Nature* 464, 1033–1038 (2010).

48. Xu et *al*. The rice endophyte *Harpophora oryzae* genome reveals evolution from a pathogen to a mutualistic endophyte. *Nat. Sc. Rep.* 4, 5783 (2014).

49. Schardl, C. L. *et al*. Plant-symbiotic fungi as chemical engineers: multi-genome analysis of the clavicipitaceae reveals dynamics of alkaloid loci. *PLoS Genet.* 9, e1003323 (2013).

50. Wang, X. *et al*. Genomic and transcriptomic analysis of the endophytic fungus *Pestalotiopsis fici* reveals its lifestyle and high potential for synthesis of natural products. *BMC Genomics* 27, 16–28 (2015).

51. Gazis, R. *et al*. The genome of *Xylona heveae* provides a window into fungal endophytism. *Fungal Biol.* 120, 26–42 (2016).

52. Jones, J. D. & Dangl, J. L. The plant immune system. *Nature* 444, 323–329 (2006).

53. Kohler, A. et *al*. Convergent losses of decay mechanisms and rapid turnover of symbiosis genes in mycorrhizal mutualists. *Nat. Genet.* 47, 410–415 (2015).

54. Hacquard, S. *et al*. A comprehensive analysis of genes encoding small secreted proteins identifies candidate effectors in *Melampsora larici-populina* (poplar leaf rust). *Mol. Plant Microbe Interact.* 25, 279–293 (2012).

55. Rawlings, N. D., Barrett, A. J. & Bateman, A. MEROPS: the peptidase database. *Nucleic Acids Res.* 38, D227–D233 (2010).

56. Tamura, K., Stecher, G., Peterson, D., Filipski, A. & Kumar, S. MEGA6: Molecular Evolutionary Genetics Analysis Version 6.0. *Mol. Biol. Evol.* 30, 2725–2729 (2013).

57. Kroken, S., Glass, N. L., Taylor, J. W., Yoder, O. C. & Turgeon, B. G. Phylogenomic analysis of type I polyketide synthase genes in pathogenic and saprobic ascomycetes. *Proc. Natl. Acad. Sci. USA* 100, 15670–15675 (2003).

58. Bushley, K. E. & Turgeon, B. G. Phylogenomics reveals subfamilies of fungal nonribosomal peptide synthetases and their evolutionary relationships. *BMC Evolutionary Biology* 10, 26 (2010).

59. Wisecaver, J. H. & Rokas, A. Fungal metabolic gene clusters – caravans travelling across genomes and environments. *Frontiers Microbiol.* 6, 161 (2015).

60. Bertrand, S., N. Bohni, S. Schnee, O. Schumpp, K. Gindro & Wolfender J.-L. Metabolite induction via microorganism co-culture: A potential way to enhance chemical diversity for drug discovery. *Biotechnology Advances* 32, 1180–1204 (2014)

61. Netzker, T. *et al.* Microbial communication leading to the activation of silent fungal secondary metabolite gene clusters. Frontiers *Microbiol.* 6, 299 (2015).

62. Cantarel, B. L. *et al*. The Carbohydrate-Active EnZymes database (CAZy): an expert resource for glycogenomics. *Nucl. Acids Res.* 37, D233–D238 (2009).

63. Lombard, V., Ramulu, H. G., Drula, E., Coutinho, P. M., & Henrissat, B. The carbohydrate-active enzymes database (CAZy) in 2013. *Nucl. Acids Res.* 42, D490–D495 (2014).

64. Vogel, J. Unique aspects of the grass cell wall. *Curr. Opin. Plant Biol.* 11, 301–307 (2008).

65. de Jonge, R. et *al*. Conserved fungal LysM effector Ecp6 prevents chitin-triggered immunity in plants. *Science* 329, 953–955 (2011).

66. Duplessis, S. *et al*. *Melampsora larici-populina* transcript profiling during germination and timecourse infection of poplar leaves reveals dynamic expression patterns associated with virulence and biotrophy. *Mol. Plant Microbe Interact.* 24, 808–818 (2011).

67. Kleemann, J. *et al*. Sequential delivery of host-induced virulence effectors by appressoria and intracellular hyphae of the phytopathogen *Colletotrichum higginsianum*. *PLoS Pathog.* 8, e1002643 (2012).

68. Guyon, K., Balagué, C., Roby, D. & Raffaele, S. Secretome analysis reveals effector candidates associated with broad host range necrotrophy in the fungal plant pathogen *Sclerotinia sclerotiorum*. *BMC Genomics* 15, 336 (2014).

69. Win, J. *et al*. Effector biology of plant-associated organisms: concepts and perspectives. *Cold Spring Harb. Symp. Quant. Biol.* 77, 235–247 (2012).

70. Veneault-Fourrey, C. *et al*. Genomic and transcriptomic analysis of Laccaria bicolor CAZome reveals insights into polysaccharides remodelling during symbiosis establishment. *Fungal Genet. Biol.* 72, 168–181 (2014).

71. Conesa, A. *et al*. Blast2GO: a universal tool for annotation, visualization and analysis in functional genomics research. *Bioinformatics* 21, 3674–3676 (2005).

72. Klimes, A., Amyotte, S. G., Grant, S., Kang, S. & Dobinson, K. F. Microsclerotia development in *Verticillium dahliae*: Regulation and differential expression of the hydrophobin gene *VDH1*. *Fungal Genet. Biol.* 45, 1525–1532 (2008).

73. Hacquard, S. *et al*. Laser microdissection and microarray analysis of *Tuber melanosporum* ectomycorrhizas reveal functional heterogeneity between mantle and Hartig net compartments. *Environ. Microbiol.* 15, 1853–1869 (2013).

74. Lahrmann, U. *et al*. Mutualistic root endophytism is not associated with the reduction of saprotrophic traits and requires a noncompromised plant innate immunity. *New Phytol.* 207, 841–857 (2015).

75. Zuccaro, A., Lahrmann, U. & Langen, G. Broad compatibility in fungal root symbioses. *Curr. Opin. Plant Biol.* 20, 135–145 (2014).

76. Duplessis, S. *et al*. Obligate biotrophy features unraveled by the genomic analysis of rust fungi. *Proc. Natl. Acad. Sci. USA* 108, 9166–9171.

77. Spanu, P. D. *et al*. Genome expansion and gene loss in powdery mildew fungi reveal functional trade-offs in extreme parasitism. *Science* 330, 1543–1546 (2010).

78. Wu, P. *et al*. Phosphate starvation triggers distinct alterations of genome expression in Arabidopsis roots and leaves. *Plant Physiol.* 132, 1260–1271 (2003).

79. Péret, B., Clément, M., Nussaume, L. & Desnos, T. Root developmental adaptation to phosphate starvation: better safe than sorry. *Trends Plant Sci.* 16, 442–450 (2011).

80. Lin, W. D. *et al*. Coexpression-based clustering of Arabidopsis root genes predicts functional modules in early phosphate deficiency signaling. *Plant Physiol.* 155, 1383–1402 (2011).

81. Gruber, B. D., Giehl, R. F., Friedel, S. & von Wirén, N. Plasticity of the Arabidopsis root system under nutrient deficiencies. *Plant Physiol.* 163, 161–179 (2013).

82. Smith, S. E. & Smith, F. A. Roles of arbuscular mycorrhizas in plant nutrition and growth: new paradigms from cellular to ecosystem scales. *Annu. Rev. Plant Biol.* 62, 227–250 (2011).

83. Pant, B. D *et al*. The transcription factor PHR1 regulates lipid remodeling and triacylglycerol accumulation in *Arabidopsis thaliana* during phosphorus starvation. *J. Exp. Bot.* 66, 1907–1918 (2015).

84. Tjellström, H., Andersson, M. X., Larsson, K. E. & Sandelius, A. S. Membrane phospholipids as a phosphate reserve: the dynamic nature of phospholipid-to-digalactosyl diacylglycerol exchange in higher plants. *Plant Cell Environ.* 31, 1388–1398 (2008).

85. Narang, R. A., Bruene, A. & Altmann, T. Analysis of phosphate acquisition efficiency in different Arabidopsis accessions. *Plant Physiol.* 124, 1786–1799 (2000).

86. Felten, J., *et al*. The ectomycorrhizal fungus *Laccaria bicolor* stimulates lateral root formation in poplar and Arabidopsis through auxin transport and signaling. *Plant Physiol.* 151, 1991–2005 (2009).

87. Ditengou, F.A. *et al*. Volatile signalling by sesquiterpenes from ectomycorrhizal fungi reprogrammes root architecture. *Nat. Commun.* 6, 6279 (2015).

88. Splivallo, R., Fischer, U., Göbel, C., Feussner, I. & Karlovsky, P. Truffles regulate plant root morphogenesis via the production of auxin and ethylene. *Plant Physiol.* 150, 2018–2029 (2009).

89. Rasmussen, S. *et al*. Transcriptome responses to combinations of stresses in Arabidopsis. *Plant Physiol.* 161, 1783–1794 (2013).

90. Smith, S. E. & Read, D. J. Mycorrhizal Symbiosis, Ed 3. Academic Press, New York (2008).

91. Bruce, A., Smith, S. E. & Tester, M. The development of mycorrhizal infection in cucumber: effects of P supply on root growth, formation of entry points and growth of infection units. *New Phytol.* 127, 507–514 (1994).

92. Balzergue, C., Chabaud, M., Barker, D. G., Bécard, G. & Rochange, S. F. High phosphate reduces host ability to develop arbuscular mycorrhizal symbiosis without affecting root calcium spiking responses to the fungus. *Front Plant Sci.* 4, 426 (2013).

93. Loth-Pereda, V. *et al*. Structure and expression profile of the phosphate Pht1 transporter gene family in mycorrhizal Populus trichocarpa. *Plant Physiol.* 156, 2141–2154 (2011).

94. Walder, F. *et al*. Plant phosphorus acquisition in a common mycorrhizal network: regulation of phosphate transporter genes of the Pht1 family in sorghum and flax. *New Phytol.* 205, 1632–1645 (2015).

95. Bücking, H. & Shachar-Hill, Y. Phosphate uptake, transport and transfer by the arbuscular mycorrhizal fungus *Glomus intraradices* is stimulated by increased carbohydrate availability. *New Phytol.* 165, 899–911 (2005).

96. González, E., Solano, R., Rubio, V., Leyva, A. & Paz-Ares, J. PHOSPHATE TRANSPORTER TRAFFIC FACILITATOR1 is a plant-specific SEC12-related protein that enables the endoplasmic reticulum exit of a high-affinity phosphate transporter in Arabidopsis. *Plant Cell* 17, 3500–3512 (2005).

97. Bustos, R. *et al*. A central regulatory system largely controls transcriptional activation and repression responses to phosphate starvation in Arabidopsis. *PLoS Genet.* 6, e1001102 (2010)

98. Lu, Y. T. et *al*. Transgenic plants that express the phytoplasma effector SAP11 show altered phosphate starvation and defense responses. *Plant Physiol.* 164, 1456–1469 (2014).

99. Pieterse, C. M., Van der Does, D., Zamioudis, C., Leon-Reyes, A. & Van Wees, S. C. Hormonal modulation of plant immunity. *Annu. Rev. Cell Dev. Biol.* 28, 489–521 (2012).

100. Glazebrook, J. Contrasting mechanisms of defense against biotrophic and necrotrophic pathogens. *Annu. Rev. Phytopathol.* 43, 205–227 (2005).

101. Pieterse, C. M., Leon-Reyes, A., Van der Ent, S. & Van Wees, S. C. Networking by small-molecule hormones in plant immunity. *Nat. Chem. Biol.* 5, 308–316 (2009).

102. Millet, Y. A., *et al*. Innate immune responses activated in Arabidopsis roots by microbe-associated molecular patterns. *Plant Cell* 22, 973–990 (2010).

103. Clay, N. K., Adio, A. M., Denoux, C., Jander, G. & Ausubel, F. M. Glucosinolate metabolites required for an Arabidopsis innate immune response. *Science* 323, 95–101 (2009).

104. Nafisi, M. *et al*. Arabidopsis cytochrome P450 monooxygenase 71A13 catalyzes the conversion of indole-3-acetaldoxime in camalexin synthesis. *Plant Cell* 19, 2039–2052 (2007).

105. Pant, B. D. *et al*. Identification of primary and secondary metabolites with phosphorus status-dependent abundance in *Arabidopsis*, and of the transcription factor PHR1 as a major regulator of metabolic changes during phosphorus limitation. *Plant cell environ.* 38, 172–187 (2015).

106. Khatabi, B. *et al*. Ethylene supports colonization of plant roots by the mutualistic fungus *Piriformospora indica*. *PLoS One* 7, e35502 (2012).

107. Plett, J.M. *et al*. Ethylene and jasmonic acid act as negative modulators during mutualistic symbiosis between Laccaria bicolor and Populus roots. *New Phytol.* 202, 270–286 (2014).

108. Pandey, S.P. & Somssich, I.E. The role of WRKY transcription factors in plant immunity. *Plant Physiol.* 150, 1648–1655 (2009).

109. Palmieri, L., *et al*. Molecular identification of three Arabidopsis thaliana mitochondrial dicarboxylate carrier isoforms: organ distribution, bacterial expression, reconstitution into liposomes and functional characterization. *Biochem. J.* 410, 621–629 (2008).

110. Damm, U., Mostert, L., Crous, P. W. & Fourie, P. H. Novel *Phaeoacremonium* species associated with necrotic wood of Prunus trees. *Persoonia* 20, 87-102 (2008).

111. Gardes, M. & Bruns, T. D. ITS primers with enhanced specificity for basidiomycetes - application to the identification of mycorrhizae and rusts. *Mol. Ecol.* 2, 113-118 (1993).

112. White, T. J., Bruns, T., Lee. S. & Taylor, J. Amplification and direct sequencing of fungal ribosomal RNA genes for phylogenetics. In *PCR Protocols: a guide to methods and applications* (eds Innis, M.A., Gelfand, D.H., Sninsky, J.J. & White, T.J.) 315-322 (Academic Press, San Diego, 1990).

113. Carbone, I. & Kohn, L. M. A method for designing primer sets for speciation studies in filamentous ascomycetes. *Mycologia* 91, 553-556 (1999).

114. Woudenberg, J. H. C., Aveskamp, M. M., de Gruyter, J., Spiers, A. G. & Crous, P. W. Multiple *Didymella* teleomorphs are linked to the *Phoma clematidina* morphotype. *Persoonia* 22, 56-62 (2009).

115. Crous, P. W., Groenewald, J. Z., Risede, J. M. & Hywel-Jones, N. L. *Calonectria* species and their *Cylindrocladium* anamorphs: species with sphaeropedunculate vesicles. *Stud. Mycol.* 50, 415-430 (2004).

116. Guerber, J. C., Liu, B., Correll, J. C. & Johnston, P. R. Characterization of diversity in *Colletotrichum acutatum* sensu lato by sequence analysis of two gene introns, mtDNA and intron RFLPs, and mating compatibility. *Mycologia* 95, 872-895 (2003).

117. Rambaut, A. Sequence Alignment Editor. Version 2.0. Department of Zoology. University of Oxford, Oxford (2002).

118. Swofford, D. L. PAUP*. Phylogenetic Analysis Using Parsimony (*and Other Methods). Version 4. (Sinauer Associates, Sunderland, Massachusetts, USA, 2003).

119. Hillis, D. M. & Bull, J. J. An empirical test of bootstrapping as a method for assessing confidence in phylogenetic analysis. *Syst. Biol.* 42, 182-192 (1993).

120. Van Dongen, S. Graph Clustering by Flow Simulation. (University of Utrecht, 2000).

121. Katoh, K., Misawa, K., Kuma, K. & Miyata, T. MAFFT: a novel method for rapid multiple sequence alignment based on fast Fourier transform. *Nucleic Acids Res.* 30, 3059–3066 (2002).

122. Darriba, D., Taboada, G. L., Doallo, R. & Posada, D. ProtTest 3: fast selection of best-fit models of protein evolution. *Bioinformatics* 27, 1164–1165 (2011).

123. Salichos, L. & Rokas, A. Inferring ancient divergences requires genes with strong phylogenetic signals. *Nature* 497, 327–331 (2013).

124. Sanderson, M. J. r8s: inferring absolute rates of molecular evolution and divergence times in the absence of a molecular clock. *Bioinformatics* 19, 301 –302 (2003).

125. Collemare, J., Billard, A., Böhnert, H. U. & Lebrun, M-H. Biosynthesis of secondary metabolites in the rice BLAST fungus *Magnaporthe grisea*: the role of hybrid PKS-NRPS in pathogenicity. *Mycol. Res.* 112, 207–215 (2008).

126. Mitchell, A. *et al*. The InterPro protein families database: the classification resource after 15 years. *Nucl. Acids Res.* 43, D213–221 (2015).

127. Edgar, R. C. MUSCLE: multiple sequence alignment with high accuracy and high throughput. *Nucl. Acids Res.* 32, 1792–1797 (2004).

128. Finn, R. D., Clements, J. & Eddy, S. R. HMMER web server: interactive sequence similarity searching. *Nucl. Acids Res.* 39, W29–W37 (2011).
